# Correlation analysis of secondary metabolites, metal elements, and anti-BSA denaturation activity in different parts of *Rhododendron tomentosum* from China

**DOI:** 10.3389/fpls.2026.1900896

**Published:** 2026-08-14

**Authors:** Suping Xiao, Meiyu Cao, Qiong Yin, Pengfei Liu, Bin Xie, Luodan Ouyang, Jiyong Wang, Pengfei Tu

**Affiliations:** 1 School of Pharmaceutical Sciences, Peking University, Beijing, China; 2 Technology Research and Development Department, China National Traditional Chinese Medicine Co., Ltd., Beijing, China; 3 State Key Laboratory for Quality Ensurance and Sustainable Use of Dao-Di Herbs, National Resource Center for Chinese Materia Medica, China Academy of Chinese Medical Sciences, Beijing, China

**Keywords:** coumarins, flavonoid glycosides, anti-BSA denaturation activity, metal elements, correlation analysis, ITS sequence, Rhododendron tomentosum

## Abstract

**Background:**

*Rhododendron tomentosum* (also known as *Ledum palustre* L.) is a traditional medicinal plant, yet the accumulation patterns of secondary metabolites and the distribution of metal elements across its different parts (roots, old branches, young branches, branches and leaves, leaves, flowers, and fruits) remain largely unclear.

**Methods:**

UPLC-MS, HPLC, ICP-MS, and chemometric tools were employed to analyze the secondary metabolites (predominantly flavonoids) and the contents of Al, Fe, Cu, Mg, K, Ca, Mn, and Zn in different parts of Chinese *R. tomentosum*. The anti-inflammatory activity was evaluated by the inhibition rate against bovine serum albumin (BSA) denaturation.

**Results:**

Three coumarin compounds—the glycosides aesculin and fraxin, and their aglycone fraxetin—were present in all parts. Aesculin and fraxin were abundant in young branches, old branches, and roots. Hyperoside, guaijaverin, isoquercitrin, and avicularin were mainly distributed in leaves and young branches. Quercitrin was abundant in flowers, followed by fruits, with the highest levels observed in samples rich in reproductive organs. The contents of Al and Fe were relatively high in roots, whereas the levels of Mg, K, Ca, and Mn were relatively low in roots and old branches. Correlation analysis revealed that Al and Fe showed significant positive correlations with coumarins (aesculin and fraxin), while Al exhibited significant negative correlations with flavonoid glycosides (hyperoside, guaijaverin, isoquercitrin, avicularin, and quercitrin). Mg, K, Ca, and Mn were significantly negatively correlated with coumarins but significantly positively correlated with flavonoid glycosides. Cu was present at relatively low levels and showed significant negative correlations with aesculin and fraxin. Significant positive correlations were also observed between Al and Fe, Al and Zn, and Fe and Zn, as well as among Mn, Mg, K, and Ca. K was positively correlated with Cu, and Mn was positively correlated with Zn. Conversely, Mg was negatively correlated with Al, and K was negatively correlated with both Al and Fe. Inhibition rates of roots, old branches, and young branches were lower than those of branches and leaves, leaves, fruits, and flowers. Flowers and fruits exhibited high inhibition rates. The inhibition rate was negatively correlated with aesculin and fraxin, and positively correlated with hyperoside, guaijaverin, isoquercitrin, avicularin, and quercitrin.

**Conclusion:**

This study provides new insights into the intrinsic relationships among “DNA molecular identification – different parts – chemical composition – function” in Chinese *R. tomentosum*, facilitating its artificial cultivation, selective breeding, and comprehensive utilization. Specifically, the findings suggest that: (1) *R. tomentosum* may serve as a candidate woody species for studying the co-accumulation of coumarins with Al/Fe under acidic soil conditions, although this hypothesis requires experimental validation; (2) Al and Fe are highly co-accumulated and may synergistically influence secondary metabolite accumulation in different parts together with Mg and K, also warranting further testing; (3) Hyperoside, avicularin, guaijaverin, isoquercitrin, and quercitrin may be the major potential anti-BSA denaturation components based on correlation and *in vitro* activity assays; and (4) Combining DNA barcoding (ITS sequences) and flavonoid profiling may help distinguish broad-leaved and fine-leaved types of Chinese *Ledum* species, pending further verification.

## 1 Introduction

Investigating the correlation between plant secondary metabolites and metal elements is of significant importance. Under metal stress, plants modulate their metabolic processes by synthesizing various secondary metabolites, some of which directly enhance stress tolerance through scavenging reactive oxygen species (Anjitha et al., 2021). Different plant parts may contain distinct bioactive compounds and exhibit diverse interaction mechanisms with metals (Nobahar et al., 2021). Certain flavonoids protect plants against heavy metal stress by acting as efficient chelators of metals such as Cu, Zn and Fe, thereby preventing the generation of hydroxyl radicals via the Fenton reaction (Williams et al., 2004). For instance, high levels of catechin and quercetin have been identified in the root exudates of maize (*Zea mays* L.) grown in aluminum (Al)-contaminated soil (Kidd et al., 2001).

The secondary metabolites of medicinal plants are closely linked to their bioactivities. In recent years, numerous studies have explored the relationship between phytochemical fingerprints and biological activities (Ayhan et al., 2025; Baky et al., 2025; Navarrete-Carriola et al., 2024; Sun et al., 2026). The *in vitro* protein denaturation method using bovine serum albumin (BSA) as a protein model is frequently employed to evaluate the anti-arthritic potential of various essential oils (Marrelli et al., 2020). Amodeo et al. (2019) and Musolino et al. (2023) used the anti-BSA denaturation assay to assess the anti-inflammatory and anti-denaturation properties of plants such as *Chenopodium album* L. and *Sisymbrium officinale* (L.) Scop. Spectrum-effect relationship research, a relatively popular approach, has provided a new strategy for the quality control of traditional Chinese medicine and has been widely applied (Cao et al., 2023; Xu et al., 2022). For example, Cui et al. (2018) investigated the dynamic changes of secondary metabolites (flavonoids) during the flowering period of *Malus halliana* Koehne and their spectrum-effect relationship with α-glucosidase inhibition, revealing both positive and negative correlations between component peaks and α-glucosidase inhibitory activity.

In the last century, Several scholars (Harborne and Williams, 1971; Griesbach, 1987), studied the flavonoids of *Rhododendron* species to explore the relationship between chemical composition and systematics. They found that quercetin monoglycosides are a common feature of the genus *Rhododendron*, while myricitrin, gossypitrin, and quercetin-3, 7-dimethyl ether are concentrated in specific taxa, possessing unique taxonomic value. Flavonoids and flavonoid glycosides have also been reported as key characteristic components of the phenolic profile of *Rhododendron groenlandicum* (Eid et al., 2016).

*Rhododendron tomentosum*(*R. tomentosum*, syn. *Ledum palustre L.*, *Ledum palustre* var. *dilatatum* Wahlenb., *Ledum palustre* var. *angustum* E. A. Busch, *Rhododendron palustre* Turcz. ex DC., *Ledum tomentosum* Stokes) (Wu et al., 2005)is an evergreen dwarf shrub belonging to the family Ericaceae. Formerly classified under the genus *Ledum*, it is currently placed within the subgenus *Rhododendron* (Harmaja, 1990). This species is rich in flavonoid glycosides, flavonoids, triterpenoids, and volatile oils, and exhibits anti-inflammatory, antioxidant, and anti-hyperuricemic activities (Singh et al., 2021). Other species within the same taxonomic group have also been reported to possess antibacterial, antitumor, hypoglycemic, hepatoprotective, neuroprotective, and cardioprotective effects (Vengrytė and Raudonė, 2024). In China, the native *Ledum* species primarily include *Ledum palustre* var. *palustre*, *L. palustre* var. *dilatatum* Wahlenb (Wu et al., 2005), and *R. tomentosum* subsp. *decumbens* (Aiton) Elven & D. F. Murray (syn. *L. decumbens*, *R. subarcticum, R. tomentosum* subsp. *subarcticum*, *L. palustre* var. *decumbens*). Among these, *R. tomentosum* subsp. *decumbens* is treated as a subspecies of *R. tomentosum* in the *Flora of China*. *R. tomentosum* is mainly distributed in Northeast China and eastern Inner Mongolia (Wu et al., 2005) and is considered a circum-Arctic species. However, morphological similarities among these taxa and the lack of clear diagnostic characters often complicate species authentication, particularly for samples collected from different geographical origins.

In this study, DNA barcoding based on ITS sequences was first employed to authenticate the origin of *L. palustre* var. *palustre* and *L. palustre* var. *dilatatum* collected from different regions of China. UPLC-Q-TOF-MS, HPLC, and chemometric tools were then used to comparatively analyze differential secondary metabolites (primarily flavonoid glycosides) in different parts of *R. tomentosum* from China. ICP-MS was applied to determine the contents of Al, Fe, Cu, Zn, Mg, K, Ca, and Mn in these plant parts, followed by correlation analysis between metal elements and secondary metabolites, aiming to elucidate potential drivers of secondary metabolite variation under the stress-prone, environmental conditions typical of its boreal and subarctic habitats. The anti-inflammatory potential of different plant parts was evaluated using the BSA denaturation inhibition assay, and a dose-effect relationship was analyzed to screen for potential bioactive components.

Overall, this study provides new insights into the intrinsic relationships among plant part, chemical composition (metal elements and secondary metabolites), and bioactivity in medicinal plants, which may facilitate the artificial cultivation, selective breeding, and comprehensive utilization of *R. tomentosum*.

## 2 Materials and methods

### 2.1 Plant material and sample collection

**Figure 1:**
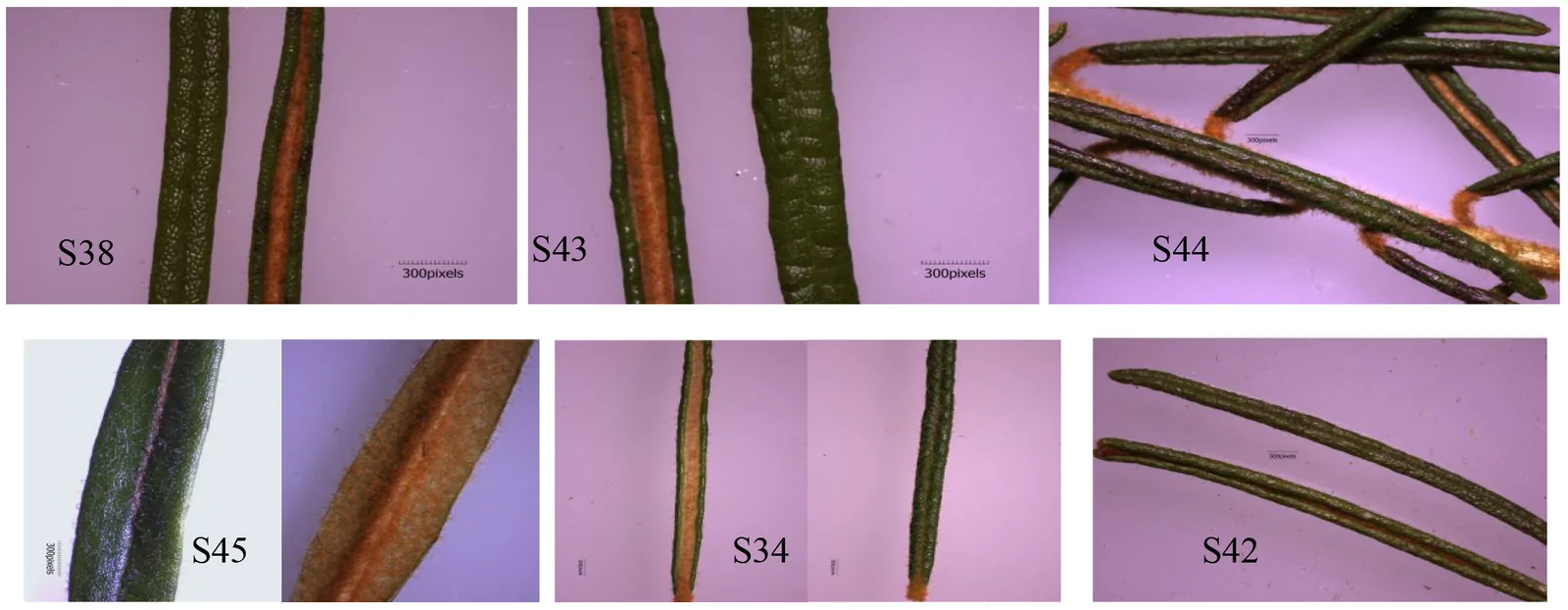
Stereomicroscopic images of fresh leaves of *R. tomentosum* (magnification: ×10). S34: fine-leaved type from Tahe County, Daxing’anling Prefecture, Heilongjiang; S38: fine-leaved type from Genhe City, Daxing’anling Prefecture, Inner Mongolia; S42: fine-leaved type from Mohe City, Daxing’anling Prefecture, Heilongjiang; S43: fine-leaved type from Shangganling, Yichun City, Lesser Khingan Mountains, Heilongjiang; S44: fine-leaved type from the northern slope of Changbai Mountain Nature Reserve, Jilin; S45: broad-leaved type from the northern slope of Changbai Mountain Nature Reserve, Jilin.

A total of 51 batches of *R. tomentosum* were collected from the Changbai Mountain region (Jilin Province) and Genhe City in the Greater Khingan Mountains (Inner Mongolia), China, between June 2024 and September 2025. All collected plant materials were naturally dried in a cool, well-ventilated area. Detailed source information for each batch is provided in **Table 1**. For DNA extraction, leaves were collected from 14 individuals of batch S34 and 10 individuals from each of batches S38, S42, S43, S44, and S45, as illustrated in **Figure 1** (sampling locations for DNA extraction). Voucher specimens were deposited in the Department of Science and Technology Research and Development, China National Traditional Chinese Medicine Co., Ltd., and were authenticated as *R. tomentosum* by Prof. Pengfei Tu.

**Table 1:** Source information of *R. tomentosum* samples.

Batch no.	Collection time	Origin code	Leaf characteristic	Part
S1	2025.09	D	Broad	Root
S2	2025.09	D	Fine	Root
S3	2025.09	B	Fine	Root
S4	2025.09	C	Fine	Root
S5	2025.09	A	Fine	Root
S6	2025.09	E	Fine	Root
S7	2025.09	D	Broad	Old branch
S8	2025.09	D	Fine	Old branch
S9	2025.09	B	Fine	Old branch
S10	2025.09	C	Fine	Old branch
S11	2025.09	A	Fine	Old branch
S12	2025.09	E	Fine	Old branch
S13	2025.09	D	Broad	Young branch
S14	2025.09	D	Fine	Young branch
S15	2025.09	B	Fine	Young branch
S16	2025.09	C	Fine	Young branch
S17	2025.09	A	Fine	Young branch
S18	2025.09	E	Fine	Young branch
S19	2025.09	D	Broad	Young branch and leaf
S20	2025.09	D	Fine	Young branch and leaf
S21	2025.09	B	Fine	Young branch and leaf
S22	2025.09	C	Fine	Young branch and leaf
S23	2025.09	A	Fine	Young branch and leaf
S24	2025.09	E	Fine	Young branch and leaf
S25	2024.06	A	Fine	Leaf
S26	2024.07	A	Fine	Leaf
S27	2024.08	A	Fine	Leaf
S28	2024.09	A	Fine	Leaf
S29	2024.09	B	Fine	Leaf
S30	2024.09	C	Fine	Leaf
S31	2025.06	A	Fine	Leaf
S32	2025.07	A	Fine	Leaf
S33	2025.08	A	Fine	Leaf
S34	2025.09	A	Fine	Leaf
S35	2025.06	B	Fine	Leaf
S36	2025.07	B	Fine	Leaf
S37	2025.08	B	Fine	Leaf
S38	2025.09	B	Fine	Leaf
S39	2025.06	C	Fine	Leaf
S40	2025.07	C	Fine	Leaf
S41	2025.08	C	Fine	Leaf
S42	2025.09	C	Fine	Leaf
S43	2025.09	E	Fine	Leaf
S44	2025.09	D	Fine	Leaf
S45	2025.09	D	Broad	Leaf
S46	2025.08	B	Fine	Fruit
S47	2025.08	C	Fine	Fruit
S48	2025.08	A	Fine	Fruit
S49	2025.08	B	Fine	Flower
S50	2025.08	C	Fine	Flower
S51	2025.08	A	Fine	Flower

Note: Origin codes correspond to the following locations:.

A = Tahe County, Daxing’anling Prefecture, Heilongjiang (124.68°E, 52.41°N, ca. 472 m, GCJ02);

B = Genhe City, Daxing’anling Prefecture, Inner Mongolia (121.53°E, 50.90°N, ca. 800 m, WGS84);

C = Mohe City, Daxing’anling Prefecture, Heilongjiang (122.46°E, 52.35°N, ca. 849 m, WGS84);

D = Northern slope of Changbai Mountain Nature Reserve, Jilin (128.14°E, 42.05°N, ca. 775 m, GPS);

E = Shangganling, Youhao District, Yichun City, Lesser Khingan Mountains, Heilongjiang (129.02°E, 47.97°N, ca. 845 m, WGS84).

### 2.2 DNA barcoding and ITS sequence analysis

#### 2.2.1 Total DNA extraction

Each plant sample was surface-sterilized with 75% ethanol and air-dried. After drying, 0.1 g of tissue was ground into powder using liquid nitrogen. Genomic DNA was extracted following the manufacturer’s protocol of the Plant Genomic DNA Extraction Kit. The extracted DNA was stored at −20 °C until further use.

#### 2.2.2 PCR amplification and sequencing

The internal transcribed spacer (ITS) region was amplified using universal plant primers ITS1F (5′-GCATATCAATAAGCGGAGGA-3′) and ITS4R (5′-CCTTGTTACGACTTTTACTTCC-3′), synthesized by Beijing Rui Biomedical Technology Co., Ltd. The polymerase chain reaction (PCR) was performed in a 25 μL reaction mixture containing 12.5 μL of 2× Taq PCR Master Mix II (including Taq DNA polymerase, dNTPs, and Mg²^+^), 1 μL each of forward and reverse primers (10 μmol/L), 1 μL of template DNA (approximately 20–50 ng), and ddH_2_O to a final volume of 25 μL. The amplification program was as follows: initial denaturation at 94 °C for 5 min; followed by 30 cycles of denaturation at 94 °C for 1 min, annealing at 50 °C for 1 min, and extension at 72 °C for 90 s (with an additional 3 s per cycle); and a final extension at 72 °C for 7 min. The PCR products were stored at 4 °C for short-term use or at −20 °C for long-term preservation. All amplified products were sent to Beijing Rui Biomedical Technology Co., Ltd. for electrophoresis detection and bidirectional sequencing.

#### 2.2.3 Sequence processing and analysis

Forward and reverse sequencing chromatograms were proofread, trimmed of primer regions and low-quality ends, and assembled using CodonCode Aligner software to obtain complete sequences for each primer. The obtained ITS sequences were submitted to the National Center of Biotechnology Information (NCBI) database to obtain GenBank accession numbers. To expand the comparison scope and enhance species identification reliability, additional ITS sequences of former *Ledum* species, *Rhododendron ferrugineum*, and representatives of sections *Ponticum* and *Vireya* were downloaded from the NCBI database (https://www.ncbi.nlm.nih.gov). Multiple sequence alignments were performed using CLC Sequence Viewer 8.0 and MEGA 12.0 software. Intraspecific genetic distances were calculated using the Kimura 2-parameter (K2P) model. A phylogenetic tree was constructed using the neighbor-joining (NJ) method with 1, 000 bootstrap replicates to evaluate branch support.

### 2.3 Methods for the identification and detection of secondary metabolites

#### 2.3.1 Preparation of sample solutions

Each dried powder of *R. tomentosum* (0.5 g, passed through a No. 4 sieve) was accurately weighed and placed into a 100 mL conical flask. Then, 20 mL of 50% methanol was added, and the mixture was weighed. After ultrasonic extraction for 30 min, the solution was cooled and centrifuged at 3, 000 rpm for 5 min. The supernatant was collected and filtered through a 0.45 μm membrane filter to obtain the sample solution.

#### 2.3.2 Preparation of reference standard solutions

Various reference standards were accurately weighed, transferred into 10 mL volumetric flasks, and diluted to volume with methanol. The flasks were ultrasonicated until the standards were completely dissolved, yielding reference standard solutions with known concentrations.

#### 2.3.3 UHPLC-Q-TOF-MS conditions

Chromatographic conditions: Chromatographic separation was performed on a Welch Ultimate AQ-C18 column (4.6 × 250 mm, 5 µm). The column temperature was maintained at 30 °C, the flow rate was set at 0.3 mL/min, and the injection volume was 10 μL. The detection wavelength was set to 360 nm, with a scanning range of 190–400 nm. The mobile phase consisted of acetonitrile (A) and 0.1% formic acid in water (B). The gradient elution program was as follows: 0–5 min, 5%–10% A; 5–95 min, 10%–24% A; 95–102 min, 24%–60% A; 102–105 min, 60%–95% A; 105–108 min, 95% A; 108–110 min, 95%–5% B; 110–120 min, 5% A (with B as the complementary solvent).

Mass spectrometric conditions: Mass spectrometry was performed using an electrospray ionization (ESI) source in both positive and negative ion modes. The mass range was set to *m/z* 50–1, 700. The following parameters were applied: nebulizer pressure, 35 psi; drying gas temperature, 320 °C; drying gas flow rate, 8 L/min; sheath gas temperature, 350 °C; sheath gas flow rate, 11 L/min; capillary voltage, 4, 000 V; nozzle voltage, 1, 000 V; fragmentor voltage, 175 V; cone voltage, 60 V. The MS/MS mass range was *m/z* 50–1, 700, with a collision energy of ±40 eV. Reference masses were used for calibration: positive ion mode, *m/z* 121.0509 and 922.0098; negative ion mode, *m/z* 112.9855 and 1033.9881.The chemical constituents in the samples were characterized by integrating MS fragmentation patterns (MSFP), literature data, and high-resolution mass spectrometry databases (HRMSD) for natural products.

#### 2.3.4 HPLC conditions

HPLC was performed on a Thermo BDS HYPERSIL C18 column (5 μm, 250 × 4.6 mm). The mobile phase consisted of acetonitrile (A) and 0.1% phosphoric acid in water (B). The detection wavelength was 360 nm, the column temperature was maintained at 30 °C, the flow rate was 1.0 mL/min, and the injection volume was 10 μL. The gradient elution program was similar to that used in the UHPLC-Q-TOF-MS analysis.

### 2.4 Identification of common peaks and similarity analysis

The AIA data of the chromatograms obtained from the 51 batches of *R. tomentosum* samples were imported into the *Similarity Evaluation System for Chromatographic Fingerprint of Traditional Chinese Medicine* (Version 2012). Using the chromatograms of samples S1, S7, S13, S19, S25, S46, and S49 (representing roots, old branches, young branches, branches with leaves, leaves, fruits, and flowers, respectively) as reference spectra, a mean-based reference fingerprint was generated with a time window width of 0.1 min. Multipoint calibration was performed using the designated reference peaks to generate the HPLC fingerprints and corresponding reference fingerprints for the 51 batches, and the similarity values were calculated.

Subsequently, the reference fingerprints generated for roots, old branches, young branches, branches with leaves, leaves, fruits, and flowers were imported into the same similarity evaluation system. Using the flower reference fingerprint as the reference spectrum, a mean-based reference fingerprint for all groups was generated with a time window width of 0.1 min. Similarity values and peak area percentages (calculated by the normalization method) were determined for each group.

### 2.5 Chemical pattern recognition

#### 2.5.1 Cluster analysis

The chromatograms of the 51 batches of *R. tomentosum* samples were integrated for peaks with an area greater than 10, 000 and retention times between 4.5 min and 95 min. A total of 86 peak areas were obtained for each batch (**Supplementary Table 1**). Missing values were replaced with zero. These data were imported into the Weishengxin online platform (https://www.bioinformatics.com.cn) to generate a cluster analysis dendrogram using the 86 chemical component contents as variables.

#### 2.5.2 PCA and OPLS-DA modeling and validation

The 86 peak areas per batch were then imported into SIMCA 14.1 software. Variables with only one or two peak areas were removed, resulting in 60 peak areas per batch for principal component analysis (PCA). Pareto scaling was applied, and a PCA model was established.

To further discriminate among samples, an orthogonal partial least squares discriminant analysis (OPLS-DA) model was constructed using the peak areas of the 60 components from the 51 batches as variables in SIMCA 14.1.

To evaluate the predictive performance and robustness of the 3-class OPLS-DA model, a customized 7-fold cross-validation strategy was employed. Considering the limited sample size of the flower group (n = 3), this group was permanently assigned to the training set in each iteration. For the remaining two groups—Group 2 (roots, old branches, and young branches, n = 18) and Group 3 (branches with leaves, leaves, and fruits, n = 30)—a stratified 7-fold split was applied to ensure that each fold maintained a class proportion approximating that of the combined dataset (Group 2:Group 3 ≈ 37.5:62.5). Specifically, the leaves (n = 21) were evenly distributed with three samples per fold; roots, old branches, young branches, and branches with leaves (n = 6 each) were distributed across 5–6 folds; and fruits (n = 3) were assigned to the first three folds. The resulting seven folds each contained approximately 3–5 samples from Group 2 and 3–5 samples from Group 3, with the overall group ratio preserved as closely as possible. The detailed assignment of each sample to specific cross-validation folds is provided in **Supplementary Table 2**.

Internal validation was further performed using 200 permutation tests. Model performance was evaluated based on cumulative R²X, R²Y, and Q² values (where R²X and R²Y represent the explained variation in X and Y, respectively, and Q² indicates the predictive ability). A Q² value > 0.5 was considered indicative of good predictive ability, and the intercept of the Q² regression line from the permutation test was used as the primary criterion to rule out overfitting (Eriksson et al., 2006).

Variable importance in projection (VIP) values were extracted, and 10 differential metabolites with VIP > 1.0 were identified. A VIP score plot was generated to visualize the discriminating markers.

### 2.6 Metal element analysis

The determination of Al, Fe, Cu, Mg, K, Ca, Mn and Zn contents was performed following the method described in the National Food Safety Standard GB 5009.268-2025: Determination of Multi-elements in Foods.

#### 2.6.1 Sample preparation

Each sample (0.5 g) was accurately weighed and placed into a WX-8000 microwave digestion vessel (Shanghai Yiyao Instrument Technology Development Co., Ltd.). Then, 5 mL of nitric acid was added, and the vessel was capped and left overnight. After tightening the cap, the samples were digested using a microwave digestion system according to the program described below. Following digestion, the vessel was cooled, and the cap was carefully opened to release any pressure. The inner cap was rinsed with a small amount of water, and the digestion vessel was degassed by ultrasonication. The digest was then diluted to 50 mL with water, mixed well, and kept for analysis. A blank test was performed simultaneously.

The microwave digestion program was set as follows: 80 °C for 3 min (15 atm); 120 °C for 3 min (20 atm); 150 °C for 3 min (25 atm); 170 °C for 3 min (30 atm); 190 °C for 3 min (35 atm); and 210 °C for 20 min (40 atm).

#### 2.6.2 Instrument conditions

An Agilent 7850 inductively coupled plasma mass spectrometer (ICP-MS) was used for metal element analysis. The following parameters were applied: points per peak, 3; replicates, 3; detection mode, manual; sample uptake rate, 0.3 rps; helium flow rate, 4.3 mL/min; RF power, 1, 550 W; sampling depth, 8 mm; nebulizer gas flow rate, 1.05 L/min; spray chamber temperature, 2 °C; dilution gas flow rate, 0.0 L/min; general mode, He collision mode; kinetic energy discrimination voltage, 5.0 V; sampling cone/skimmer cone, Ni-plated Cu/Ni; nebulizer, MicroMist concentric nebulizer; torch injector tube inner diameter, 2.5 mm.

#### 2.6.3 Standard curves

Single-element standard stock solutions of Al, Fe, Cu, Mg, K, Ca, Mn, and Zn, as well as a multi-element mixed internal standard stock solution containing Ge, Rh, Sc, and In, were diluted stepwise with a nitric acid solution (5 + 95) to prepare mixed standard solutions and an internal standard working solution of appropriate concentration (e.g., 0.5–200 µg/L for each element). Blank solutions and standard solutions were introduced via a peristaltic pump, and calibration curves were automatically generated by the instrument.

### 2.7 Method validation

The analytical method was validated in terms of linearity, limit of detection (LOD), limit of quantitation (LOQ), accuracy, precision, and stability. Linearity was evaluated by constructing calibration curves for each compound/element, and the correlation coefficients (R) were calculated. LOD and LOQ were determined based on the signal-to-noise ratio (S/N = 3:1 and 10:1, respectively). Accuracy was assessed through spiked recovery experiments at low, medium, and high concentration levels, and precision was expressed as relative standard deviation (RSD) of triplicate measurements. Sample stability was evaluated by analyzing the same sample at different time points (e.g., 0, 2, 4, 8, 12, and 24 h). All validation parameters met the acceptance criteria specified in the relevant guidelines.

### 2.8 Anti-bovine serum albumin denaturation assay

#### 2.8.1 Preparation of 2% BSA solution

Bovine serum albumin (BSA, 2 g) was dissolved in 100 mL of 0.2 mol/L acetate buffer (pH 5.6) to obtain a 2% BSA solution.

#### 2.8.2 Preparation of sample solutions

An aliquot (2 mL) of the sample solution was evaporated to dryness at 90 °C in a water bath to remove ethanol, and the residue was reconstituted with deionized water to a final volume of 2 mL. The solution was freshly prepared, stored at 4 °C, and used within 48 h.

#### 2.8.3 Preparation of reference standard solutions

Appropriate amounts of reference standards were accurately weighed and diluted to volume with deionized water. For reference standards not readily soluble in water, a small amount of methanol was added initially to dissolve the compound, followed by dilution with 0.2 mol/L acetate buffer (pH 5.6) and ultrasonication until a clear solution was obtained. The solution was then diluted to volume, mixed well, and stored at 4 °C for use within 48 h.

#### 2.8.4 Assay procedure

An aliquot (100 μL) of the sample solution was mixed with 400 μL of deionized water and 500 μL of 2% BSA solution. Separately, an aliquot (500 μL) of the reference standard solution was mixed with 500 μL of 2% BSA solution. The mixtures were incubated at 37 °C for 20 min, then heated to 70 °C for 20 min with shaking on a thermostatic mixing incubator, followed by immediate cooling on ice to room temperature. The turbidity of each sample was measured at 660 nm and recorded as A_1_. For the control, 500 μL of 2% BSA solution was mixed with 500 μL of the corresponding solvent (acetate buffer or deionized water). The same incubation and measurement procedure was followed, and the turbidity was recorded as A_2_. All measurements were performed in triplicate. The inhibition rate was calculated using the following formula:


Inhibition rate (%)=(A1-A2)/A1×100%

### 2.9 Correlation analysis

The inhibition rates were calculated using Origin 2021 software. Spearman correlation analysis among the differential marker peak areas, metal element contents, and inhibition rates against bovine serum albumin (BSA) denaturation in different parts of *R. tomentosum* was performed using the Weishengxin online platform (https://www.bioinformatics.com.cn).

## 3 Results

### 3.1 ITS sequence identification results

For the experimental leaf samples collected from six different geographical origins, the PCR amplification and sequencing success rate of the internal transcribed spacer (ITS) region was 100%. The obtained ITS sequences ranged from 696 to 717 bp in length, with GC contents ranging from 53.45% to 53.60%.

**Figure 2:**
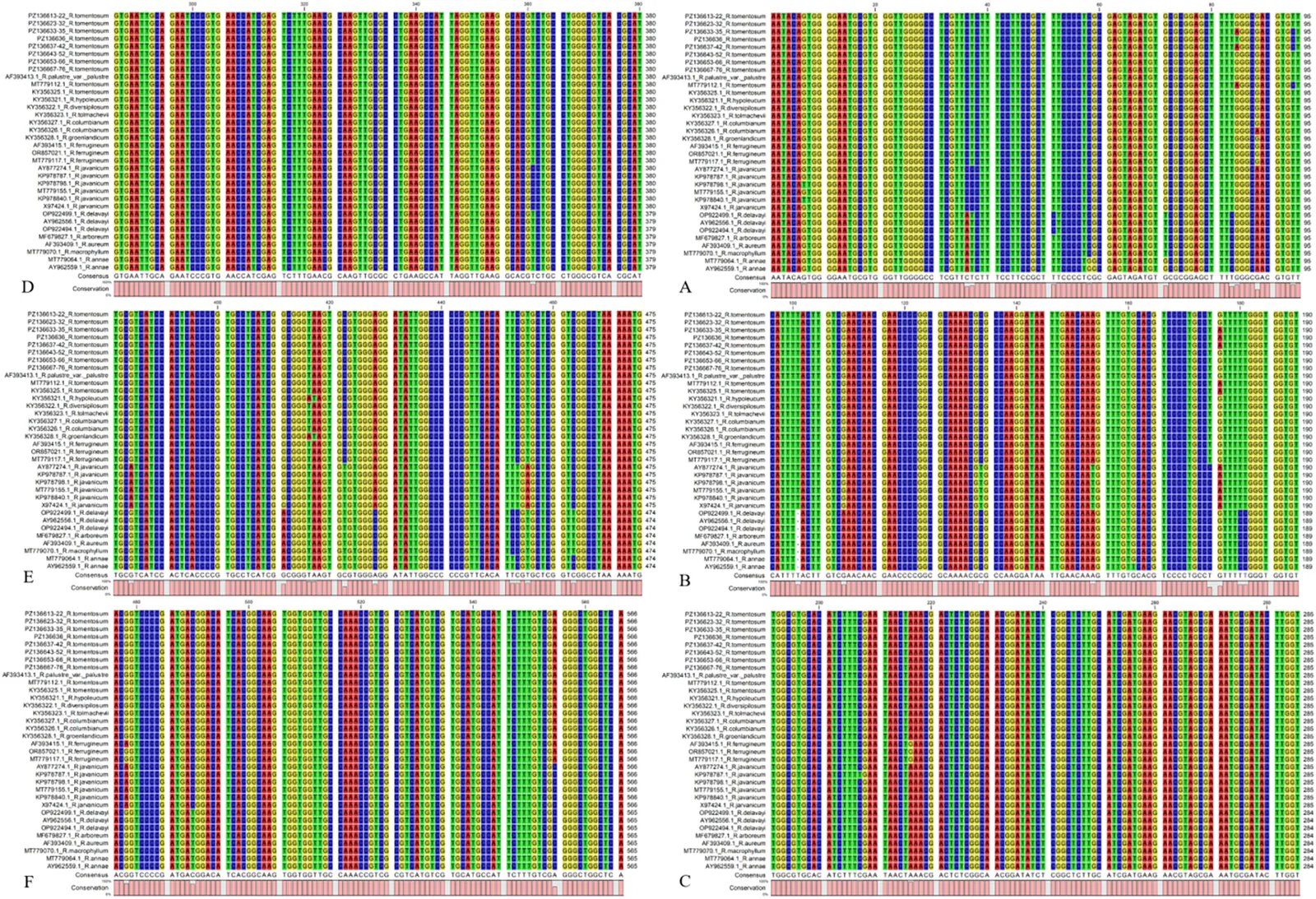
Multiple alignment of ITS sequences of the samples. **(A)** ITS sequences (positions 1–95 bp); **(B)** ITS sequences (positions 96–190 bp); **(C)** ITS sequences (positions 191–285 bp); **(D)** ITS sequences (positions 286–380 bp); **(E)** ITS sequences (positions 381–475 bp); **(F)** ITS sequences (positions 476–566 bp). A deletion at position 101 (relative to the reference sequence) is present in eight species of *Rhododendron* sect. *Ponticum*.The sequences PZ1336613–PZ1336622 (abbreviated as PZ1336613–22) correspond to the 10 individual plant sequences from batch S44. The sequences PZ1336623–PZ1336632 (abbreviated as PZ1336623–32) correspond to the 10 individual sequences from batch S43. The sequences PZ1336633–PZ1336642 (abbreviated as PZ1336633–42) correspond to the 10 individual sequences from batch S45. The sequences PZ1336643–PZ1336652 (abbreviated as PZ1336643–52) correspond to the 10 individual sequences from batch S42. The sequences PZ1336653–PZ1336666 (abbreviated as PZ1336653–66) correspond to the 14 individual sequences from batch S34. The sequences PZ1336667–PZ1336676 (abbreviated as PZ1336667–76) correspond to the 10 individual sequences from batch S38.

As shown in **Figure 2**, nucleotide substitutions, including both transitions (purine↔purine or pyrimidine↔pyrimidine) and transversions (purine↔pyrimidine), were observed between the ITS sequences of *R. tomentosum* and those of intraspecific and interspecific plants within the same subsection *Ledum*. Specifically, for *Ledum palustre* var. *palustre*, the nucleotides at positions 84, 94, and 176 were G, T, and G, respectively, when compared with AF393413.1 (*R. palustre* var. *palustre*), KY356322.1 (*R. diversipilosum*), KY356323.1 (*R. tolmachevii*), and KY356327.1 (*R. columbianum*). For KY356325.1 (*R. tomentosum*), the nucleotides at positions 84, 94, and 176 were G, T, and A, respectively, indicating a transition (G↔A) at position 176 relative to *L. palustre* var. *palustre*.

For *Ledum palustre* var. *dilatatum* (except for the sample PZ136636, which is an accession of *R. tomentosum*), the nucleotides at positions 84, 94, and 176 were A, C, and A, respectively, when compared with MT779112.1 (*R. tomentosum*). Compared with *L. palustre* var. *palustre*, these sequences exhibited transitions at positions 84 (G→A) and 94 (T→C). In contrast, PZ136636 (*R. tomentosum*) showed T, C, and A at positions 84, 94, and 176, respectively, with a transversion (T↔A) occurring at position 84 relative to *L. palustre* var. *palustre*.

For other species within the same subsection *Ledum*, namely KY356321.1 (*R. hypoleucum*), KY356328.1 (*R. groenlandicum*), and KY356326.1 (*R. columbianum*), the nucleotides at positions 84, 94, and 176 were G, T, and G, respectively, identical to those of *L. palustre* var. *palustre*. However, when compared with *L. palustre* var. *palustre*, transitions were observed in these species at positions 417 (T in *R. hypoleucum*), 88 (A in *R. groenlandicum*), and 417 (T in *R. columbianum*).

For the subsection *Rhododendron* species, including OR857021.1 (*Rhododendron ferrugineum*), AF393415.1 (*Rhododendron ferrugineum*), and KY356328.1 (*Rhododendron groenlandicum*), the nucleotides at positions 84, 94, and 176 were G, T, and G, respectively, consistent with L. palustre var. palustre. However, a transition (G) was observed at position 216 in these species. Additionally, AF393415.1 (*R. ferrugineum*) exhibited a transition (A) at position 478.

**Figure 3:**
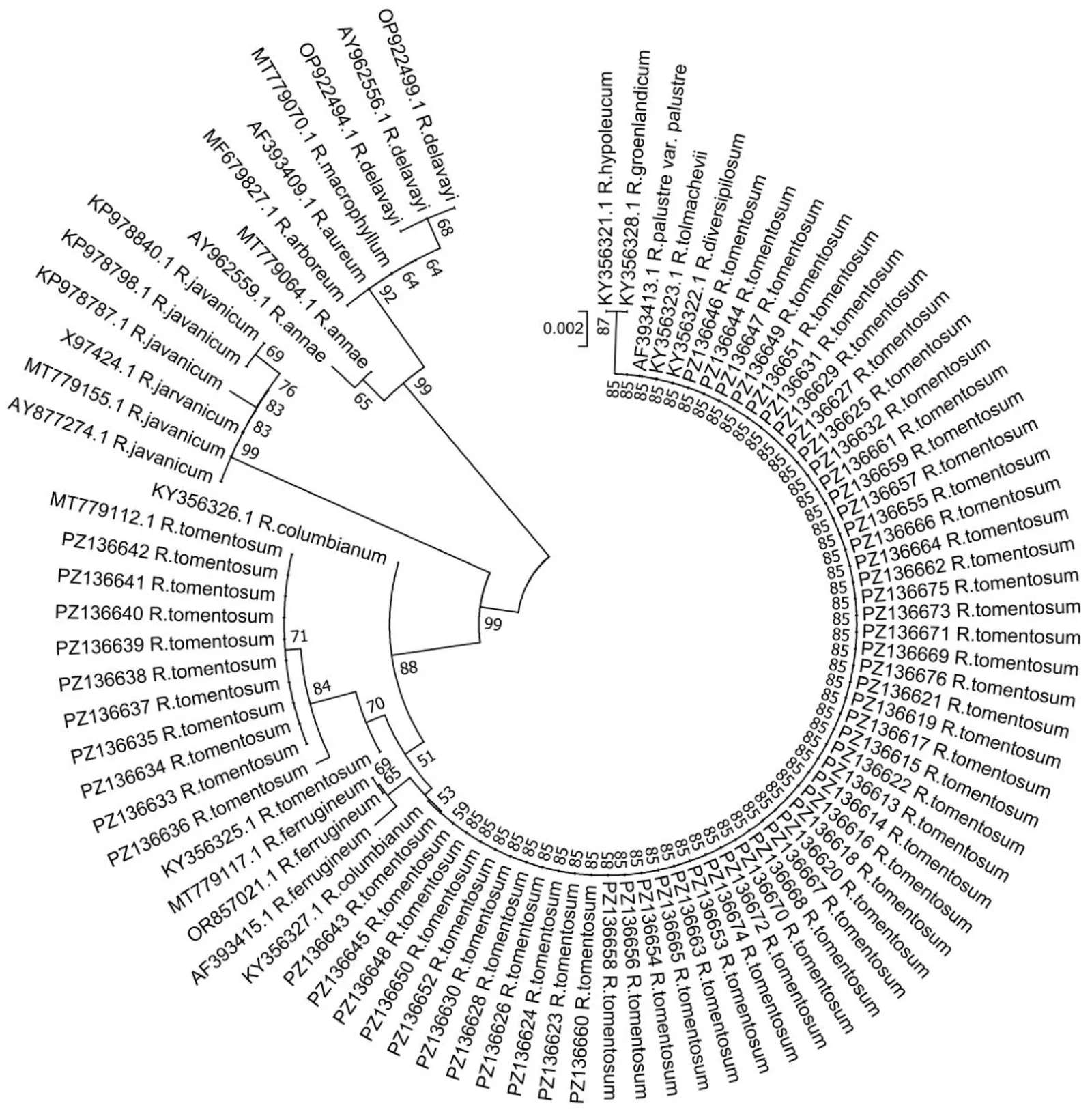
Neighbor-joining (NJ) phylogenetic tree of *R. tomentosum* and its congeneric species. Bootstrap values (1, 000 replicates) are shown at the nodes. Scale bar: genetic distance.

As shown in the NJ tree in **Figure 3**, *L. palustre* var. *palustre*, *L. palustre* var. *dilatatum*, other species of subsection *Ledum*, and *R. ferrugineum* (subsection *Rhododendron*), were clustered into one major clade. *R. javanicum* (subsection *Vireya*) formed a separate clade, and species of subsection *Ponticum* formed another clade, each with 99% bootstrap support. These phylogenetic results were further supported by the ITS sequence alignment analysis. Based on the combined evidence, the *R. tomentosum* samples collected from six different geographical origins were confidently identified as *R. tomentosum*.

### 3.2 Method validation results of fingerprint analysis

The precision, stability, and repeatability of the method were evaluated using *R. tomentosum* sample (batch S34). With hyperoside as the reference peak, the RSD values for relative retention times of the common peaks were below 0.11%, 0.15%, and 0.10% in the precision, stability, and repeatability tests, respectively, while the corresponding RSD values for the relative peak areas were below 1.66%, 1.65%, and 4.30%. These results indicate that the instrumental precision is good, the sample solution remains stable within 28 h, and the method exhibits satisfactory repeatability.

### 3.3 Establishment of HPLC fingerprints

The HPLC fingerprints and corresponding reference fingerprints of the 51 batches of *R. tomentosum* are shown in **Supplementary Figure 1**. The calculated similarity values are presented in **Supplementary Table 3**, **S4**.

For *Ledum palustre* var. *dilatatum* and *L. palustre* var. *palustre* from different geographical origins, the roots and old branches exhibited extremely high similarity, with values all above 0.994. The similarity values for young branches were above 0.97, and those for branch-leaf mixtures were above 0.979. In contrast, the similarity values for leaves showed considerable variation. Batch S40 (leaves collected from Mohe in July 2025) had a similarity value of 0.615, and batch S26 (leaves collected from Tahe in July 2024) had a similarity value of 0.889. Notably, the leaf color of these two batches was visibly darker compared to other batches, which may have been related to post-harvest handling or environmental factors. Except for these two batches, the similarity values for leaves from all other batches were above 0.926.A high similarity value was observed for fruits (0.999), while the similarity value for flowers exceeded 0.929.

When comparing different plant parts, roots, old branches, young branches, and branches with leaves showed relatively high similarity values, all above 0.967. However, leaves, fruits, and flowers exhibited greater variation, with similarity values of 0.662, 0.544, and 0.792, respectively. In addition to similarity values, the proportions of non-common peak areas in different plant parts—namely roots, old branches, young branches, branches with leaves, leaves, fruits, and flowers—showed considerable variation, displaying an increasing trend from roots to flowers.

### 3.4 Identification of compounds (primarily flavonoids)

Sample solutions of batches S45 and S44, which exhibited the most abundant chromatographic peaks, were selected for UHPLC-Q-TOF-MS analysis. The chromatograms are shown in **Supplementary Figure 2**, and the identification results of the major compounds of batch S45 are presented in **Supplementary Table 5**. At a detection wavelength of 360 nm, the compounds with relatively high peak areas included neochlorogenic acid, aesculin, fraxin, fraxetin, taxifolin 3-O-glucoside, hyperoside, isoquercitrin, quercetin 3-O-arabinoside, avicularin, guaijaverin, quercitrin, 3, 6’-di-O-feruloylsucrose, 2-(3, 4-dihydroxyphenyl)-5, 7-dihydroxy-3-[[6-O-(4-hydroxybenzoyl)-β-D-galactopyranosyl]oxy]-4H-1-benzopyran-4-one, quercetin 3-O-(6-O-acetyl-β-D-glucoside), quercetin 3-O-(6″-O-p-coumaroyl)-glucoside, quercetin 3-O-(6″-feruloyl)-β-D-galactopyranoside, and 3-[(3, 6-di-O-acetyl-β-D-galactopyranosyl)oxy]-2-(3, 4-dihydroxyphenyl)-5, 7-dihydroxy-4H-1-benzopyran-4-one, its mass spectrum of the last compound is shown in **Supplementary Figure 3**. The compound profile of batch S44 was similar to that of batch S45 (data not shown).

Reference standards of hyperoside, fraxetin, fraxin, avicularin, guaijaverin, aesculin, neochlorogenic acid, isoquercitrin, quercetin 3-O-arabinoside, quercitrin, chlorogenic acid, rutin, polydatin, and esculetin were selected for HPLC analysis, comparison, and confirmation. The retention times and UV spectra of the reference standards matched those of the corresponding peaks in the samples, confirming their identities (**Supplementary Figure 4**).

### 3.5 Chemical pattern recognition analysis results

#### 3.5.1 Cluster analysis

**Figure 4:**
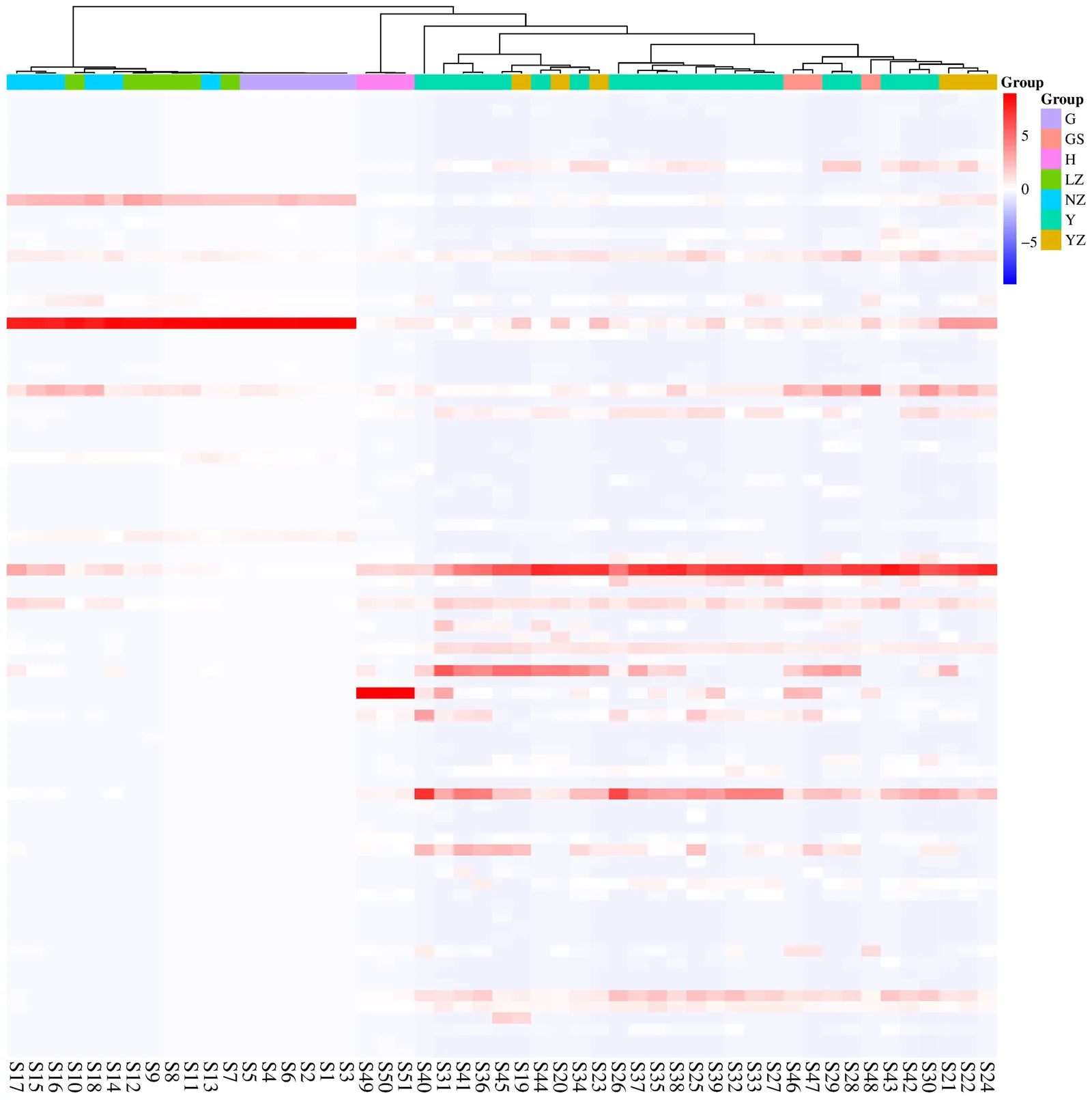
Cluster analysis dendrogram of the 51 batches of *R. tomentosum* samples based on HPLC fingerprint data. Samples are color-coded by plant part: G: root, GS: fruit, H:flower, LZ: old branch, NZ: young branch, Y: leaf, YZ: branch and leaf.

The results of the cluster analysis are shown in **Figure 4**. The 51 batches of *R. tomentosum* were classified into two major groups. Roots, old branches, and young branches were clustered into one group (S1–S18), while flowers, fruits, branches with leaves, and leaves were clustered into another group (S19–S51). Within the second group, flowers formed a distinct subcluster (S49–S51), and fruits, branches with leaves, and leaves were clustered together (S19–S48).

#### 3.5.2 Principal component analysis

**Figure 5:**
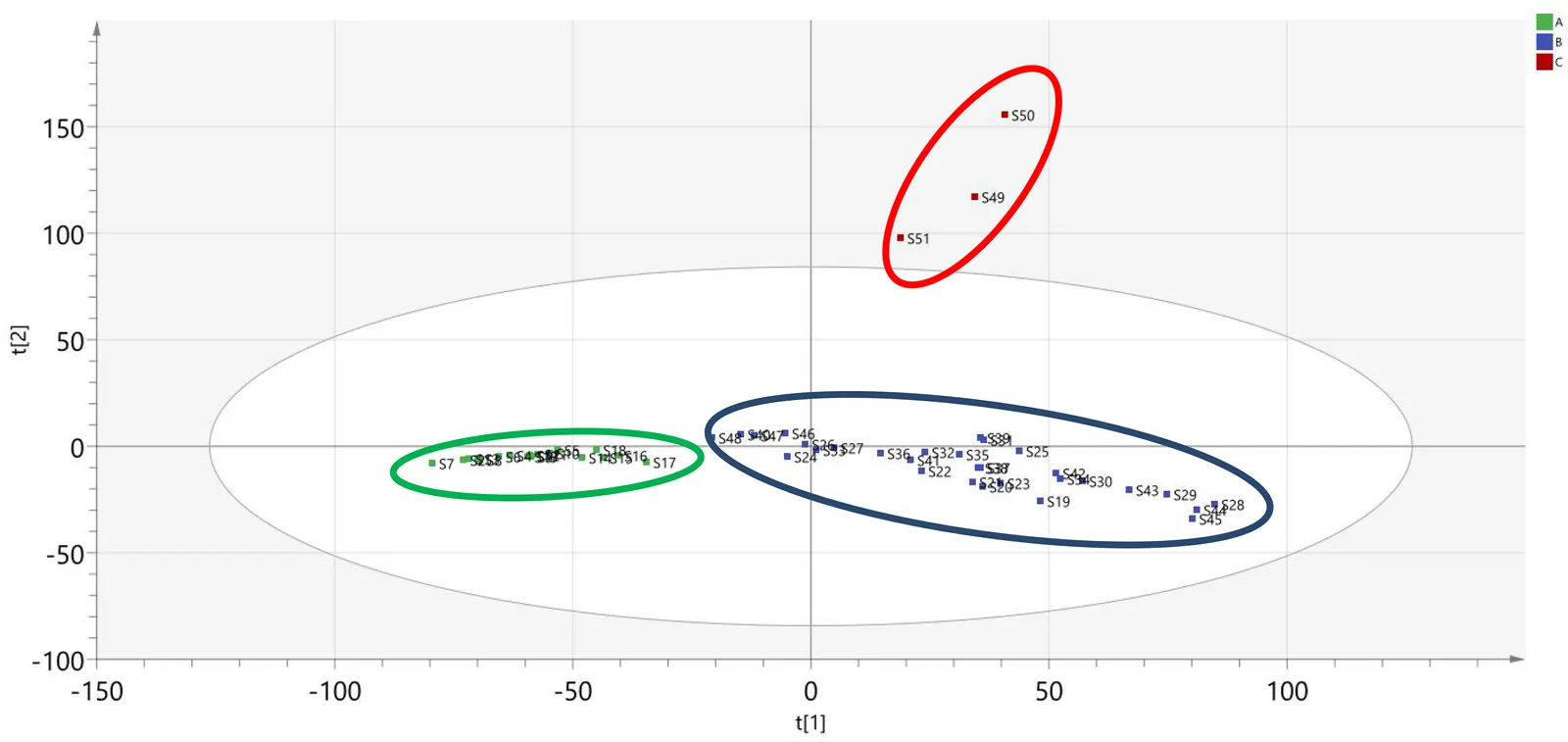
PCA score plot of 51 batches of R. tomentosum samples. showing three distribution (A–C), A: roots, old branches, and young branches (S1–S18); B: branches with leaves, leaves, and fruits (S19–S48); C: flowers (S49–S51). Ellipses represent the 95% confidence intervals.

The PCA score plot for the 51 batches of *R. tomentosum* is shown in **Figure 5**. The model explained 88.4% of the original variance (R²X = 0.884), and the predictive ability parameter Q² was 0.504. The results showed that the 51 batches could be classified into three groups: (A) roots, old branches, and young branches (S1–S18); (B) branches with leaves, leaves, and fruits (S19–S48); and (C) flowers (S49–S51), with the flower group falling outside the 95% confidence interval, indicating distinct chemical profiles. These findings were broadly consistent with the cluster analysis results, confirming significant differences in the 60 component peak areas among batches from different origins and plant parts.

#### 3.5.3 Orthogonal partial least squares discriminant analysis

**Figure 6:**
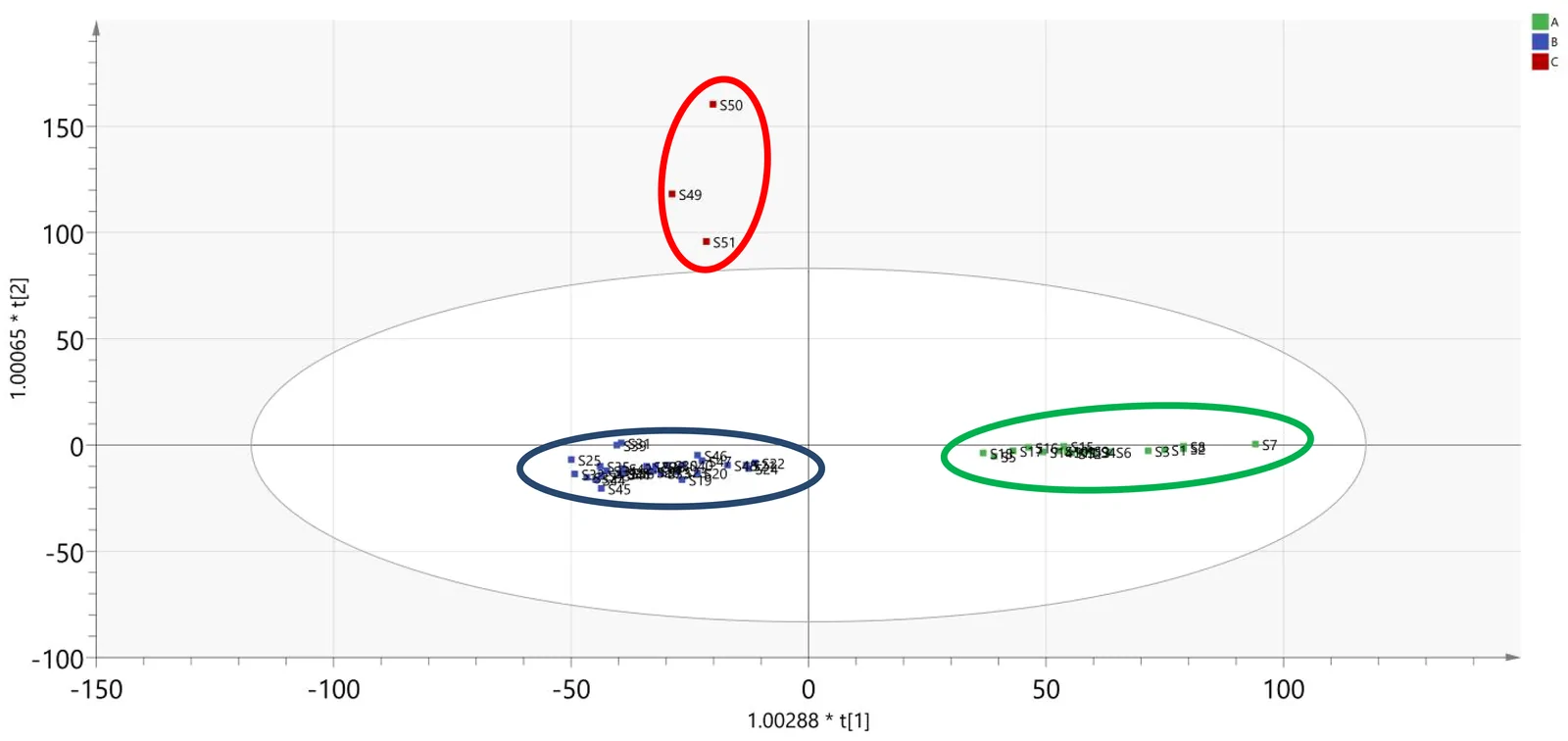
OPLS-DA score plot of 51 batches of R. tomentosum samples. showing three distinct groups (A–C), A: roots, old branches, and young branches (S1–S18); B: branches with leaves, leaves, and fruits (S19–S48); C: flowers (S49–S51). Ellipses represent the 95% confidence intervals.

The OPLS-DA score plot for the 51 batches of *R. tomentosum* is shown in **Figure 6**. The model parameters were as follows: R²X = 0.77, R²Y = 0.935, and Q² = 0.905, indicating that the model explained 77.0% of the variance in X and 93.5% in Y, with excellent predictive ability (Q² = 0.905). These results demonstrated that the OPLS-DA model had high stability, reliability, and predictive performance. As shown in the score plot, the 51 batches were classified into three groups, consistent with the results of cluster analysis (CA) and principal component analysis (PCA).

**Figure 7:**
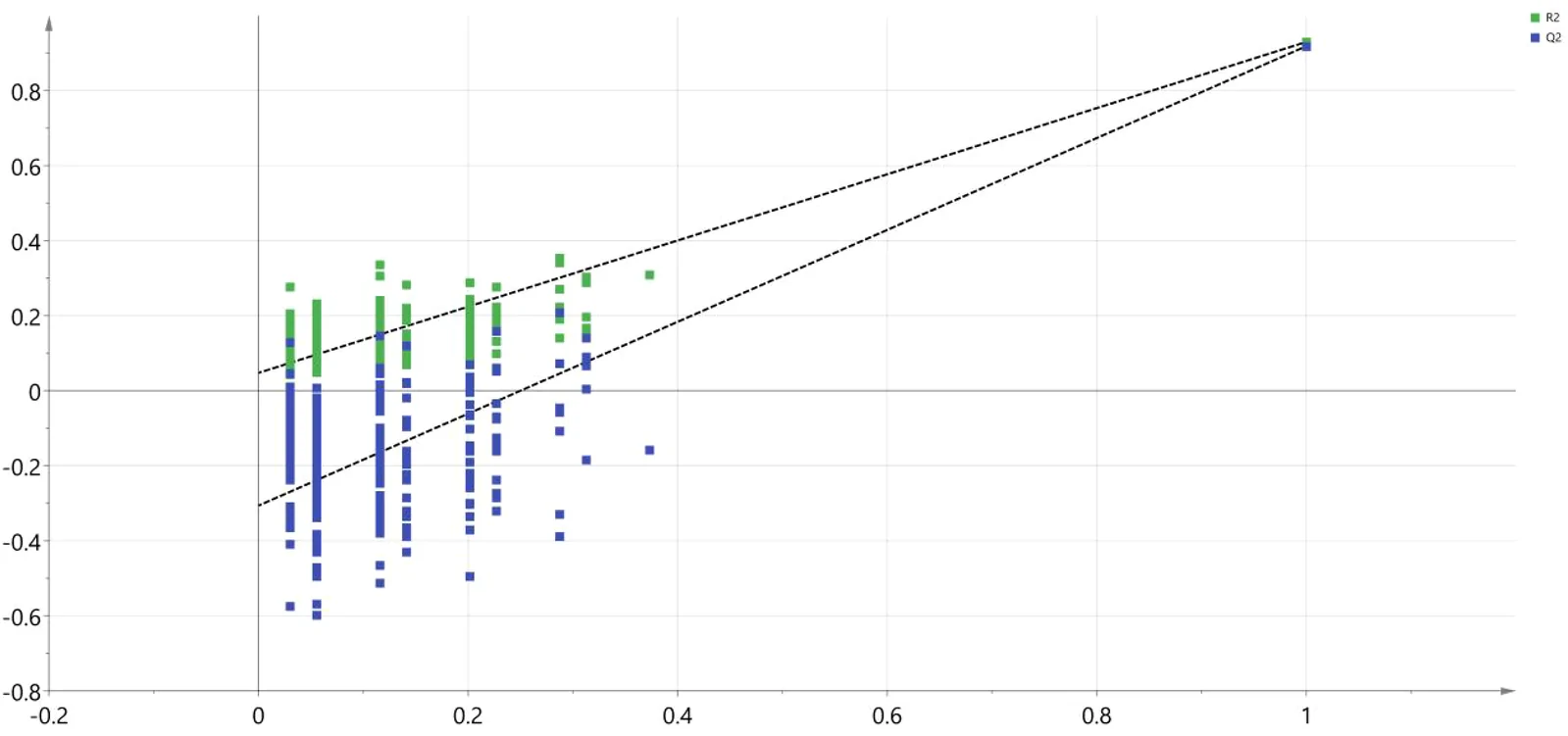
Permutation test (200 permutations) of the OPLS-DA model for 51 batches of *R. tomentosum* samples. The left panel shows the permuted R² and Q² values, while the right panel indicates the original model values.

To evaluate the robustness of the OPLS-DA model and rule out overfitting, model validation was performed using permutation tests with 200 random permutations. The permutation test results are shown in **Figure 7**. The Y-intercept of the R² and Q² regression lines were 0.0474 and −0.306, respectively, both falling within acceptable ranges for a valid model. The original R² and Q² values of the OPLS-DA model (on the right) were both higher than those obtained after random permutations (on the left), and the Q² regression line intersected the Y-axis below zero. These results confirmed the reliability and predictive ability of the established OPLS-DA model, without overfitting.

The 3-class OPLS-DA model was further validated using customized 7-fold cross-validation. The cross-validation results showed good predictive performance, with cumulative R²X = 0.644, R²Y = 0.945, and Q² = 0.855. The 200 permutation tests (**Supplementary Figure 5**) yielded a negative intercept of the Q² regression line (–0.485), and an R² intercept of 0.36, confirming that the model did not suffer from overfitting and that the observed predictive performance was not due to random chance. The small difference between R²Y and Q² (0.09) and the high Q²/R²Y ratio (0.905) further supported the robustness of the model. Collectively, these validation results indicate that the OPLS-DA model is reliable for subsequent differential metabolite screening.

**Figure 8:**
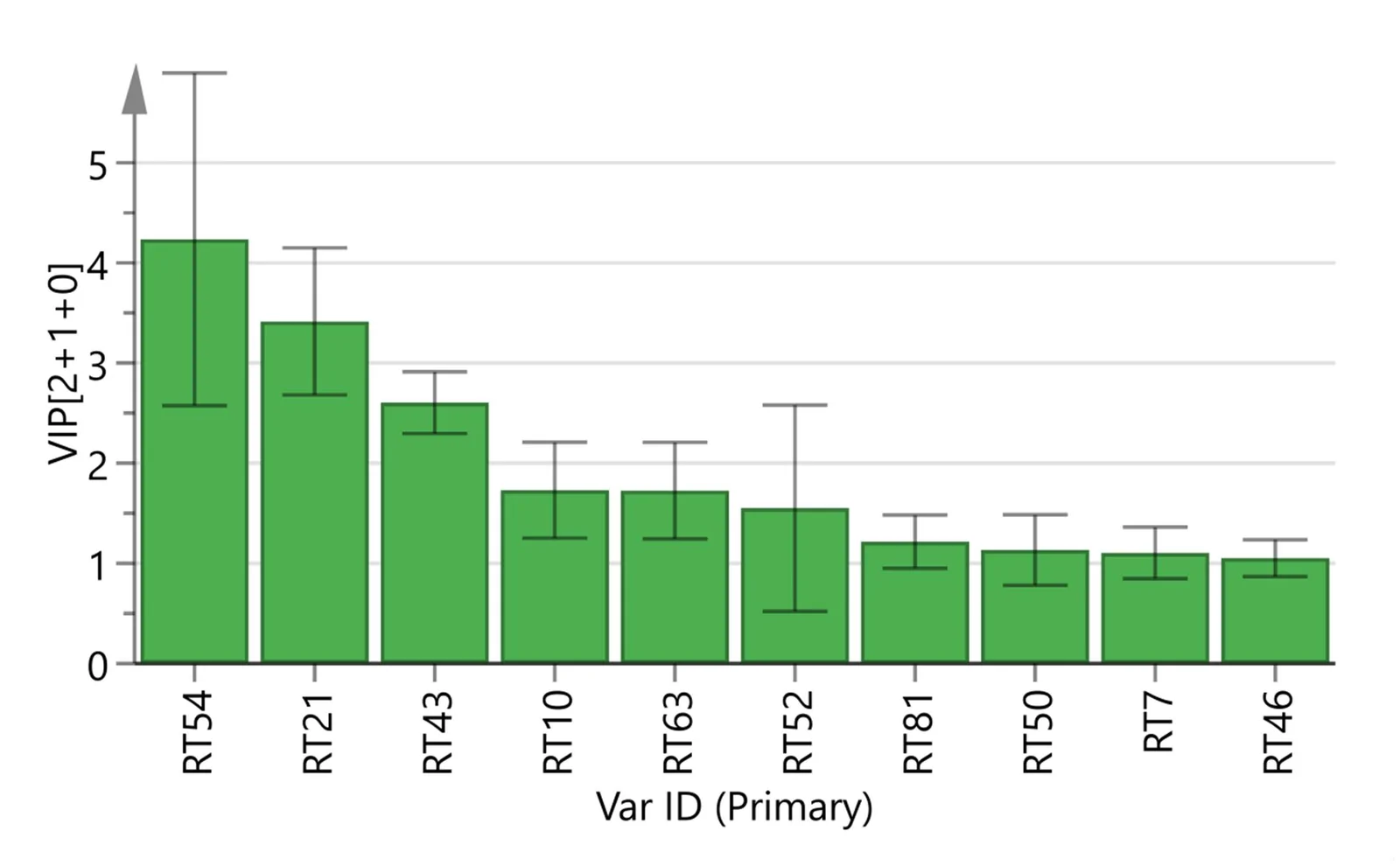
VIP plot of 10 discriminatory components identified from the OPLS-DA model for the 51 batches of *R. tomentosum* samples. Components with VIP values > 1.0 are considered significant contributors to the classification model.

Based on the validated OPLS-DA model, variable importance in projection (VIP) analysis was performed to identify the key discriminatory compounds. VIP values are positively correlated with the degree of influence on intergroup differences. VIP analysis was conducted on the peak areas of the 60 components, and a threshold of VIP > 1.0 was used to screen for potential quality difference markers. Among the 60 components, 10 exhibited VIP values >1.0 and were thus identified as potential differential markers for distinguishing different batches of *R. tomentosum*, as shown in **Table 2** and **Figure 8**, indicating that these 10 components contributed significantly to the discrimination among samples.

**Table 2:** VIP values of the 10 discriminatory components identified from the OPLS-DA model for the 51 batches of *R. tomentosum* samples.

Peak ID	Component name	VIP value	Peak ID	Component name	VIP value
RT54	Quercitrin	4.235	RT52	Guaijaverin	1.550
RT21	Fraxin	3.415	RT81	Quercetin 3-O-(6″-O-p-coumaroyl)-glucoside	1.216
RT43	Hyperoside	2.604	RT50	Avicularin	1.133
RT10	Aesculin	1.730	RT7	Neochlorogenic acid	1.104
RT63	Compound 63 (see Note)	1.726	RT46	Isoquercitrin	1.051

“RT” followed by a number indicates the retention time rank order among the 86 integrated peaks. RT63 corresponds to 2-(3, 4-dihydroxyphenyl)-5, 7-dihydroxy-3-[[6-O-(4-hydroxybenzoyl)-β-D-galactopyranosyl]oxy]-4H-1-benzopyran-4-one.

### 3.6 Method validation results for multi-component quantification

The analytical method was validated for specificity, linearity, sensitivity, precision, repeatability, stability, and accuracy. As shown in **Supplementary Table 6**, all seven compounds exhibited good linearity (R²=0.9997–0.9999) over the tested concentration ranges. The LODs and LOQs ranged from 0.51 to 3.19 ng/mL and 1.68 to 10.53 ng/mL, respectively. The precision RSD values for the peak areas of the seven analytes ranged from 0.10% to 1.37%, repeatability RSD values ranged from 1.10% to 3.72%, and stability RSD values ranged from 0.10% to 2.71% within 24 h, all meeting the acceptance criteria. The average recoveries ranged from 95.83% to 115.35%, with RSD values between 1.20% and 6.36%; the relatively higher RSD for quercitrin (6.36%) was attributed to its low content in the selected samples, as it was predominantly accumulated in flowers and fruits. These results demonstrate that the established method is reliable, precise, and suitable for the quantitative analysis of the seven target compounds in *R. tomentosum*.

### 3.7 Method validation results for metal element quantification

Similarly, the method for metal element quantification was validated for linearity, sensitivity, accuracy, precision, repeatability, and stability. As shown in **Supplementary Table 7**, all elements exhibited excellent linearity with correlation coefficients (R) ≥ 0.9999. The LODs ranged from 0.03817 μg/L (Cu) to 2.809 μg/L (K), and the LOQs ranged from 0.1145 μg/L (Cu) to 8.427 μg/L (K). The average recoveries of the eight elements ranged from 88.10% (Mn) to 101.04% (Fe), with RSD values between 3.45% (Mg) and 8.84% (Zn), all falling within the acceptable ranges (85%–110% for most elements, 80%–115% for Cu and Zn). The precision (repeatability) RSD values were ≤ 0.60% for all elements, and the intermediate precision RSD values ranged from 1.40% to 3.57%. The stability RSD values ranged from 0.74% (Zn) to 1.84% (Ca), indicating minimal fluctuation. These results demonstrate that the method is reliable, precise, and suitable for the quantitative analysis of the eight mineral elements in *R. tomentosum*.

### 3.8 Contents of multi-component, metal element, and anti-BSA denaturation activity in different parts of *R.tomentosum*, and their correlations

#### 3.8.1 Results for multi-component contents, metal element contents, anti-BSA denaturation activity

From the 10 potential quality-discriminating marker components identified by OPLS-DA, seven relatively stable and readily available coumarin and flavonoid glycosides—namely aesculin, fraxin, hyperoside, isoquercitrin, avicularin, guaijaverin, and quercitrin—were selected and quantified by HPLC. The multi-component contents, metal element contents and inhibition rates against BSA denaturation measured in 51 batches from different parts of *R.tomentosum* are summarized in **Table 3** and **Table 4**.

**Table 3:** Multi-component contents and inhibition rate against bovine serum albumin (BSA) denaturation in 51 batches of *R.tomentosum* samples.

Sample no.	Aesculin ± S D (%)	Fraxin ± S D (%)	Hyperoside ± S D (%)	Isoquercitrin ± S D (%)	Avicularin ± S D (%)	Guaijaverin ± S D (%)	Quercitrin ± S D (%)	Inhibition ± S D (%)
S1	0.221 ± 0.003	0.770 ± 0.011	0.001 ± 0.000	0.000 ± 0.000	0.000 ± 0.000	0.000 ± 0.000	0.000 ± 0.000	37.2 ± 0.001
S2	0.222 ± 0.006	0.862 ± 0.023	0.003 ± 0.000	0.005 ± 0.000	0.000 ± 0.000	0.000 ± 0.000	0.000 ± 0.000	22.22 ± 0.001
S3	0.244 ± 0.006	0.790 ± 0.006	0.002 ± 0.000	0.004 ± 0.000	0.000 ± 0.000	0.000 ± 0.000	0.000 ± 0.000	33.15 ± 0.002
S4	0.171 ± 0.002	0.705 ± 0.005	0.001 ± 0.000	0.000 ± 0.000	0.000 ± 0.000	0.000 ± 0.000	0.000 ± 0.000	42.28 ± 0.001
S5	0.127 ± 0.004	0.468 ± 0.013	0.000 ± 0.000	0.000 ± 0.000	0.000 ± 0.000	0.000 ± 0.000	0.000 ± 0.000	28.64 ± 0.002
S6	0.242 ± 0.006	0.634 ± 0.003	0.000 ± 0.000	0.000 ± 0.000	0.000 ± 0.000	0.000 ± 0.000	0.000 ± 0.000	47.63 ± 0.003
S7	0.224 ± 0.003	0.956 ± 0.015	0.003 ± 0.000	0.000 ± 0.000	0.000 ± 0.000	0.000 ± 0.000	0.000 ± 0.000	36.42 ± 0.001
S8	0.232 ± 0.000	0.827 ± 0.014	0.013 ± 0.000	0.009 ± 0.000	0.000 ± 0.000	0.000 ± 0.000	0.000 ± 0.000	23.13 ± 0.001
S9	0.247 ± 0.004	0.605 ± 0.019	0.020 ± 0.000	0.019 ± 0.001	0.000 ± 0.000	0.000 ± 0.000	0.000 ± 0.000	43.38 ± 0.001
S10	0.204 ± 0.001	0.574 ± 0.012	0.012 ± 0.000	0.010 ± 0.000	0.000 ± 0.000	0.000 ± 0.000	0.000 ± 0.000	14.21 ± 0.002
S11	0.195 ± 0.002	0.590 ± 0.010	0.012 ± 0.000	0.013 ± 0.001	0.000 ± 0.000	0.000 ± 0.000	0.000 ± 0.000	36.89 ± 0
S12	0.265 ± 0.005	0.543 ± 0.011	0.012 ± 0.001	0.008 ± 0.000	0.000 ± 0.000	0.000 ± 0.000	0.000 ± 0.000	18.25 ± 0.001
S13	0.165 ± 0.005	0.614 ± 0.017	0.009 ± 0.000	0.011 ± 0.001	0.000 ± 0.000	0.018 ± 0.001	0.000 ± 0.000	38.69 ± 0.001
S14	0.163 ± 0.001	0.616 ± 0.012	0.041 ± 0.000	0.038 ± 0.000	0.004 ± 0.000	0.040 ± 0.001	0.000 ± 0.000	44.22 ± 0.002
S15	0.219 ± 0.005	0.552 ± 0.002	0.060 ± 0.001	0.063 ± 0.001	0.007 ± 0.000	0.027 ± 0.001	0.000 ± 0.000	33.51 ± 0.001
S16	0.203 ± 0.003	0.465 ± 0.010	0.067 ± 0.002	0.058 ± 0.001	0.007 ± 0.000	0.000 ± 0.000	0.000 ± 0.000	10.29 ± 0.001
S17	0.198 ± 0.003	0.554 ± 0.016	0.100 ± 0.001	0.083 ± 0.001	0.014 ± 0.001	0.087 ± 0.002	0.000 ± 0.000	48.32 ± 0.001
S18	0.184 ± 0.003	0.367 ± 0.010	0.086 ± 0.002	0.027 ± 0.000	0.000 ± 0.000	0.000 ± 0.000	0.000 ± 0.000	46.17 ± 0.001
S19	0.056 ± 0.001	0.176 ± 0.006	0.226 ± 0.007	0.082 ± 0.000	0.082 ± 0.005	0.606 ± 0.016	0.013 ± 0.001	68.01 ± 0.002
S20	0.053 ± 0.000	0.149 ± 0.007	0.237 ± 0.004	0.068 ± 0.001	0.055 ± 0.001	0.499 ± 0.009	0.001 ± 0.000	42.57 ± 0.002
S21	0.110 ± 0.002	0.276 ± 0.009	0.197 ± 0.005	0.088 ± 0.002	0.050 ± 0.001	0.268 ± 0.010	0.002 ± 0.000	67.75 ± 0.002
S22	0.072 ± 0.000	0.221 ± 0.007	0.202 ± 0.004	0.053 ± 0.001	0.035 ± 0.000	0.012 ± 0.000	0.001 ± 0.000	61.88 ± 0.001
S23	0.057 ± 0.000	0.181 ± 0.008	0.234 ± 0.008	0.090 ± 0.000	0.056 ± 0.001	0.351 ± 0.006	0.015 ± 0.000	68.58 ± 0.001
S24	0.056 ± 0.002	0.136 ± 0.007	0.135 ± 0.001	0.025 ± 0.000	0.025 ± 0.000	0.000 ± 0.000	0.000 ± 0.000	45.79 ± 0.008
S25	0.033 ± 0.000	0.054 ± 0.001	0.158 ± 0.001	0.041 ± 0.001	0.042 ± 0.000	0.012 ± 0.000	0.030 ± 0.000	83.77 ± 0.001
S26	0.005 ± 0.000	0.015 ± 0.000	0.050 ± 0.001	0.020 ± 0.000	0.017 ± 0.001	0.027 ± 0.002	0.000 ± 0.000	66.08 ± 0.001
S27	0.016 ± 0.000	0.034 ± 0.001	0.095 ± 0.001	0.034 ± 0.000	0.023 ± 0.000	0.000 ± 0.000	0.004 ± 0.000	67.3 ± 0
S28	0.040 ± 0.001	0.064 ± 0.001	0.278 ± 0.008	0.075 ± 0.002	0.058 ± 0.001	0.429 ± 0.012	0.009 ± 0.000	63.51 ± 0.002
S29	0.037 ± 0.001	0.068 ± 0.001	0.234 ± 0.002	0.082 ± 0.002	0.066 ± 0.001	0.458 ± 0.014	0.013 ± 0.000	67.58 ± 0.001
S30	0.057 ± 0.001	0.118 ± 0.000	0.199 ± 0.000	0.055 ± 0.001	0.056 ± 0.000	0.088 ± 0.003	0.003 ± 0.000	76.31 ± 0.002
S31	0.006 ± 0.000	0.014 ± 0.000	0.077 ± 0.001	0.081 ± 0.002	0.054 ± 0.001	0.441 ± 0.002	0.110 ± 0.001	75.3 ± 0.002
S32	0.006 ± 0.000	0.011 ± 0.000	0.144 ± 0.001	0.039 ± 0.001	0.035 ± 0.001	0.000 ± 0.000	0.004 ± 0.000	85.02 ± 0.002
S33	0.011 ± 0.000	0.021 ± 0.000	0.090 ± 0.000	0.018 ± 0.001	0.020 ± 0.000	0.000 ± 0.000	0.000 ± 0.000	52.19 ± 0.001
S34	0.023 ± 0.001	0.051 ± 0.001	0.249 ± 0.007	0.067 ± 0.001	0.067 ± 0.001	0.460 ± 0.006	0.038 ± 0.001	80.68 ± 0.007
S35	0.019 ± 0.000	0.034 ± 0.000	0.154 ± 0.003	0.059 ± 0.001	0.044 ± 0.001	0.109 ± 0.004	0.028 ± 0.001	62.25 ± 0.002
S36	0.005 ± 0.000	0.008 ± 0.000	0.073 ± 0.001	0.040 ± 0.000	0.036 ± 0.001	0.195 ± 0.000	0.007 ± 0.000	60.26 ± 0.004
S37	0.019 ± 0.000	0.021 ± 0.001	0.153 ± 0.004	0.062 ± 0.002	0.049 ± 0.001	0.233 ± 0.004	0.005 ± 0.000	76.99 ± 0.003
S38	0.020 ± 0.000	0.017 ± 0.000	0.179 ± 0.002	0.057 ± 0.001	0.043 ± 0.000	0.140 ± 0.001	0.001 ± 0.000	61.6 ± 0.001
S39	0.035 ± 0.001	0.106 ± 0.000	0.198 ± 0.000	0.078 ± 0.001	0.045 ± 0.001	0.000 ± 0.000	0.083 ± 0.001	86.78 ± 0.002
S40	0.002 ± 0.000	0.007 ± 0.000	0.012 ± 0.000	0.008 ± 0.000	0.007 ± 0.000	0.036 ± 0.001	0.010 ± 0.001	25.59 ± 0.001
S41	0.010 ± 0.000	0.027 ± 0.000	0.090 ± 0.000	0.048 ± 0.001	0.037 ± 0.000	0.263 ± 0.002	0.004 ± 0.000	66.52 ± 0.005
S42	0.047 ± 0.002	0.086 ± 0.002	0.267 ± 0.002	0.056 ± 0.001	0.045 ± 0.000	0.000 ± 0.000	0.005 ± 0.000	54.84 ± 0.002
S43	0.050 ± 0.001	0.059 ± 0.001	0.371 ± 0.001	0.167 ± 0.001	0.055 ± 0.001	0.000 ± 0.000	0.001 ± 0.000	50.95 ± 0.002
S44	0.010 ± 0.001	0.015 ± 0.000	0.390 ± 0.012	0.093 ± 0.001	0.082 ± 0.002	0.789 ± 0.008	0.010 ± 0.000	59.97 ± 0.002
S45	0.035 ± 0.000	0.063 ± 0.002	0.284 ± 0.006	0.094 ± 0.002	0.115 ± 0.002	0.820 ± 0.009	0.004 ± 0.000	88.84 ± 0.004
S46	0.002 ± 0.000	0.007 ± 0.000	0.069 ± 0.000	0.035 ± 0.002	0.013 ± 0.000	0.063 ± 0.001	0.038 ± 0.000	82.54 ± 0.003
S47	0.001 ± 0.000	0.010 ± 0.000	0.037 ± 0.001	0.024 ± 0.000	0.009 ± 0.000	0.068 ± 0.001	0.021 ± 0.000	77.41 ± 0.001
S48	0.002 ± 0.000	0.011 ± 0.000	0.030 ± 0.001	0.011 ± 0.001	0.004 ± 0.000	0.000 ± 0.000	0.005 ± 0.000	79.94 ± 0.003
S49	0.010 ± 0.000	0.051 ± 0.000	0.101 ± 0.002	0.076 ± 0.002	0.034 ± 0.000	0.204 ± 0.002	0.889 ± 0.004	74.33 ± 0.004
S50	0.028 ± 0.000	0.107 ± 0.003	0.147 ± 0.001	0.092 ± 0.001	0.026 ± 0.000	0.000 ± 0.000	1.225 ± 0.019	91.71 ± 0.002
S51	0.031 ± 0.000	0.116 ± 0.003	0.097 ± 0.001	0.038 ± 0.000	0.014 ± 0.001	0.006 ± 0.000	0.771 ± 0.009	86.7 ± 0.003

**Table 4:** Contents of the eight elements in 51 batches of *R.tomentosum* samples.

Sample no.	Al ± S D(mg/g)	Fe ± S D(mg/g)	Cu ± S D(µg/g)	Zn ± S D(mg/g)	Mg ± S D(mg/g)	K ± S D(mg/g)	Ca ± S D(mg/g)	Mn ± S D(mg/g)
S1	0.535 ± 0.023	0.405 ± 0.021	4.827 ± 0.492	0.043 ± 0.000	0.524 ± 0.000	1.556 ± 0.069	2.846 ± 0.179	0.841 ± 0.046
S2	0.496 ± 0.002	0.459 ± 0.007	4.710 ± 0.423	0.047 ± 0.004	0.463 ± 0.009	1.384 ± 0.001	2.433 ± 0.202	1.102 ± 0.011
S3	1.479 ± 0.075	1.141 ± 0.057	4.459 ± 0.323	0.031 ± 0.001	0.560 ± 0.037	1.570 ± 0.097	2.271 ± 0.018	0.925 ± 0.014
S4	0.466 ± 0.024	0.304 ± 0.003	4.288 ± 0.395	0.043 ± 0.003	0.469 ± 0.037	1.555 ± 0.019	2.121 ± 0.177	1.302 ± 0.012
S5	0.211 ± 0.003	0.147 ± 0.004	2.852 ± 0.233	0.026 ± 0.002	0.317 ± 0.008	1.754 ± 0.033	1.066 ± 0.041	0.754 ± 0.030
S6	0.474 ± 0.008	0.368 ± 0.015	3.900 ± 0.019	0.031 ± 0.000	0.514 ± 0.018	2.105 ± 0.046	2.043 ± 0.074	0.973 ± 0.016
S7	0.494 ± 0.038	0.380 ± 0.003	5.289 ± 0.317	0.047 ± 0.003	0.567 ± 0.055	1.549 ± 0.074	3.793 ± 0.352	0.847 ± 0.015
S8	0.432 ± 0.029	0.350 ± 0.011	5.241 ± 0.283	0.045 ± 0.004	0.576 ± 0.031	1.468 ± 0.022	2.943 ± 0.263	1.180 ± 0.004
S9	0.498 ± 0.036	0.384 ± 0.011	3.991 ± 0.227	0.029 ± 0.001	0.533 ± 0.023	1.478 ± 0.045	3.027 ± 0.049	1.042 ± 0.016
S10	0.228 ± 0.008	0.100 ± 0.003	3.551 ± 0.081	0.031 ± 0.001	0.371 ± 0.009	1.196 ± 0.032	2.395 ± 0.101	0.870 ± 0.016
S11	0.141 ± 0.011	0.107 ± 0.005	3.919 ± 0.042	0.033 ± 0.002	0.562 ± 0.039	1.707 ± 0.026	3.143 ± 0.143	1.036 ± 0.027
S12	0.323 ± 0.018	0.249 ± 0.006	4.445 ± 0.103	0.032 ± 0.001	0.613 ± 0.028	1.866 ± 0.054	2.993 ± 0.086	1.108 ± 0.028
S13	0.140 ± 0.012	0.120 ± 0.009	4.123 ± 0.323	0.023 ± 0.003	0.475 ± 0.026	2.200 ± 0.060	2.290 ± 0.153	0.656 ± 0.044
S14	0.142 ± 0.004	0.145 ± 0.016	4.448 ± 0.500	0.029 ± 0.003	0.724 ± 0.064	3.114 ± 0.468	3.096 ± 0.021	1.071 ± 0.025
S15	0.186 ± 0.021	0.147 ± 0.000	4.727 ± 0.279	0.035 ± 0.002	0.823 ± 0.039	2.165 ± 0.134	5.712 ± 0.374	1.542 ± 0.049
S16	0.107 ± 0.002	0.072 ± 0.001	5.433 ± 0.250	0.039 ± 0.001	0.682 ± 0.029	2.095 ± 0.051	3.930 ± 0.048	1.633 ± 0.037
S17	0.090 ± 0.004	0.074 ± 0.002	5.504 ± 0.476	0.039 ± 0.002	1.027 ± 0.054	3.181 ± 0.160	4.765 ± 0.093	1.771 ± 0.044
S18	0.171 ± 0.014	0.129 ± 0.002	4.836 ± 0.152	0.038 ± 0.001	1.244 ± 0.018	4.142 ± 0.058	6.870 ± 0.395	1.692 ± 0.043
S19	0.136 ± 0.012	0.120 ± 0.012	4.277 ± 0.075	0.032 ± 0.001	1.498 ± 0.042	5.375 ± 0.127	6.256 ± 0.167	0.994 ± 0.024
S20	0.270 ± 0.005	0.245 ± 0.002	5.183 ± 0.344	0.046 ± 0.001	1.587 ± 0.059	5.368 ± 0.197	6.209 ± 0.119	1.153 ± 0.020
S21	0.291 ± 0.022	0.225 ± 0.000	4.124 ± 0.078	0.037 ± 0.002	1.404 ± 0.048	4.004 ± 0.076	6.816 ± 0.032	1.719 ± 0.002
S22	0.121 ± 0.009	0.090 ± 0.006	3.776 ± 0.075	0.038 ± 0.000	1.163 ± 0.055	3.313 ± 0.082	7.297 ± 0.103	2.116 ± 0.015
S23	0.099 ± 0.007	0.092 ± 0.007	3.944 ± 0.156	0.035 ± 0.001	1.517 ± 0.022	4.928 ± 0.444	6.492 ± 0.322	1.360 ± 0.065
S24	0.470 ± 0.030	0.354 ± 0.015	5.246 ± 0.366	0.053 ± 0.004	1.725 ± 0.107	4.558 ± 0.277	8.355 ± 0.092	2.101 ± 0.031
S25	0.224 ± 0.008	0.165 ± 0.003	5.347 ± 0.399	0.042 ± 0.004	1.716 ± 0.186	7.510 ± 0.268	5.464 ± 0.250	1.468 ± 0.033
S26	0.084 ± 0.010	0.070 ± 0.001	5.029 ± 0.284	0.032 ± 0.002	1.673 ± 0.132	6.329 ± 0.017	5.621 ± 0.391	1.509 ± 0.006
S27	0.186 ± 0.009	0.154 ± 0.008	4.991 ± 0.238	0.043 ± 0.003	2.122 ± 0.073	5.305 ± 0.173	6.654 ± 0.567	1.709 ± 0.056
S28	0.179 ± 0.015	0.195 ± 0.010	3.769 ± 0.286	0.042 ± 0.004	1.893 ± 0.126	5.634 ± 0.323	5.791 ± 0.368	1.724 ± 0.066
S29	0.105 ± 0.007	0.082 ± 0.002	3.132 ± 0.406	0.031 ± 0.003	1.596 ± 0.164	4.363 ± 0.171	6.139 ± 0.394	1.264 ± 0.029
S30	0.247 ± 0.007	0.186 ± 0.002	3.713 ± 0.116	0.044 ± 0.002	1.315 ± 0.027	3.665 ± 0.074	5.903 ± 0.186	1.459 ± 0.025
S31	0.146 ± 0.007	0.143 ± 0.006	5.340 ± 0.134	0.043 ± 0.001	1.591 ± 0.041	7.098 ± 0.365	6.703 ± 0.177	1.961 ± 0.046
S32	0.135 ± 0.011	0.130 ± 0.008	5.550 ± 0.196	0.037 ± 0.001	1.595 ± 0.090	5.730 ± 0.310	5.999 ± 0.418	1.769 ± 0.083
S33	0.124 ± 0.004	0.107 ± 0.001	4.872 ± 0.083	0.029 ± 0.000	1.961 ± 0.081	4.424 ± 0.146	7.125 ± 0.239	2.190 ± 0.036
S34	0.081 ± 0.005	0.076 ± 0.006	4.166 ± 0.322	0.036 ± 0.001	1.649 ± 0.129	5.294 ± 0.127	6.461 ± 0.120	1.432 ± 0.026
S35	0.288 ± 0.004	0.197 ± 0.005	4.002 ± 0.437	0.037 ± 0.004	1.461 ± 0.123	4.736 ± 0.310	6.412 ± 0.455	2.144 ± 0.099
S36	0.202 ± 0.004	0.192 ± 0.009	4.579 ± 0.299	0.034 ± 0.001	1.431 ± 0.062	5.219 ± 0.141	6.306 ± 0.093	1.523 ± 0.053
S37	0.142 ± 0.013	0.118 ± 0.006	3.222 ± 0.136	0.031 ± 0.001	1.339 ± 0.151	4.459 ± 0.128	7.251 ± 0.259	2.093 ± 0.041
S38	1.715 ± 0.045	1.344 ± 0.028	4.073 ± 0.121	0.035 ± 0.001	1.618 ± 0.107	4.086 ± 0.136	7.380 ± 0.265	1.660 ± 0.059
S39	0.217 ± 0.016	0.143 ± 0.003	4.150 ± 0.452	0.043 ± 0.004	1.202 ± 0.112	5.657 ± 0.146	6.953 ± 0.462	2.589 ± 0.048
S40	0.084 ± 0.009	0.079 ± 0.002	5.281 ± 0.138	0.030 ± 0.001	1.596 ± 0.139	6.296 ± 0.189	6.539 ± 0.375	1.652 ± 0.071
S41	0.105 ± 0.009	0.089 ± 0.002	4.393 ± 0.091	0.030 ± 0.001	1.482 ± 0.080	5.593 ± 0.165	6.174 ± 0.240	1.543 ± 0.059
S42	0.089 ± 0.011	0.062 ± 0.002	3.441 ± 0.075	0.034 ± 0.001	1.321 ± 0.058	4.218 ± 0.195	7.446 ± 0.313	2.391 ± 0.078
S43	0.451 ± 0.002	0.382 ± 0.028	5.211 ± 0.161	0.055 ± 0.002	1.734 ± 0.083	4.676 ± 0.261	8.480 ± 0.351	2.184 ± 0.038
S44	0.216 ± 0.012	0.266 ± 0.002	6.129 ± 0.332	0.050 ± 0.001	2.066 ± 0.214	6.831 ± 0.574	5.972 ± 0.211	1.088 ± 0.089
S45	0.138 ± 0.005	0.129 ± 0.007	4.694 ± 0.192	0.032 ± 0.002	1.677 ± 0.106	6.488 ± 0.070	6.521 ± 0.198	1.047 ± 0.026
S46	0.158 ± 0.010	0.166 ± 0.002	6.172 ± 0.499	0.029 ± 0.003	1.173 ± 0.074	5.785 ± 0.452	4.034 ± 0.183	0.878 ± 0.006
S47	0.104 ± 0.011	0.118 ± 0.004	6.552 ± 0.063	0.028 ± 0.001	1.314 ± 0.086	6.297 ± 0.210	4.593 ± 0.172	1.175 ± 0.033
S48	0.194 ± 0.012	0.169 ± 0.011	5.886 ± 0.268	0.031 ± 0.002	1.175 ± 0.064	3.968 ± 0.172	3.908 ± 0.201	1.299 ± 0.061
S49	0.140 ± 0.012	0.138 ± 0.001	7.002 ± 0.101	0.036 ± 0.002	1.183 ± 0.012	8.225 ± 0.123	5.053 ± 0.265	1.343 ± 0.024
S50	0.199 ± 0.010	0.178 ± 0.004	5.943 ± 0.217	0.038 ± 0.001	1.174 ± 0.034	7.141 ± 0.425	6.329 ± 0.183	2.077 ± 0.008
S51	0.130 ± 0.011	0.134 ± 0.002	7.298 ± 0.310	0.032 ± 0.002	1.495 ± 0.020	6.530 ± 0.080	4.697 ± 0.231	1.397 ± 0.034

As shown in **Table 3** and **4**, the inhibition rates, component contents, and metal element contents varied considerably among different parts of *R. tomentosum*. With the exception of batch S40 (leaves from Mohe collected in July 2025), which exhibited a relatively low inhibition rate of 25.59%, and the branches with leaves of the fine-leaved type from Yichun and Changbai Mountain, the inhibition rates of roots, old branches, and young branches were generally below 50%, lower than those of branches with leaves, leaves, fruits, and flowers. In contrast, flowers and fruits showed high inhibition rates exceeding 74%.

Quercitrin content was notably high in flowers. Aesculin and fraxin were detected in all samples, with higher contents observed in roots, old branches, and young branches. Hyperoside was detected in 50 of the 51 batches across different parts, with the exception of batch S5 (roots), and showed higher contents in branches with leaves and leaves collected in September from different origins. The contents of coumarins (aesculin and fraxin) and flavonoid glycosides (hyperoside, isoquercitrin, avicularin, and guaijaverin) in *R. tomentosum* from the Changbai Mountain origin were generally higher than those from other origins.

Among the eight mineral elements determined, K and Ca were present at the highest levels, followed by Mn and Mg. A strongly synergistic accumulation pattern was observed among K, Ca, and Mg. Likewise, Al and Fe exhibited a highly synergistic accumulation trend, though with considerable variations across samples. Specifically, batches S3 and S38 contained markedly higher levels of Al (1.479 mg/g and 1.715 mg/g, respectively) and Fe (1.141 mg/g and 1.344 mg/g, respectively) than the other batches, whereas batch S42 displayed the lowest Fe content (0.062 mg/g) and a relatively low Al content (0.089 mg/g). The overall contents of Al and Fe were lower than those of K, Ca, Mn, and Mg, while Zn and Cu were found at even lower concentrations with relatively small inter-sample differences.

#### 3.8.2 Correlation analysis results

**Figure 9:**
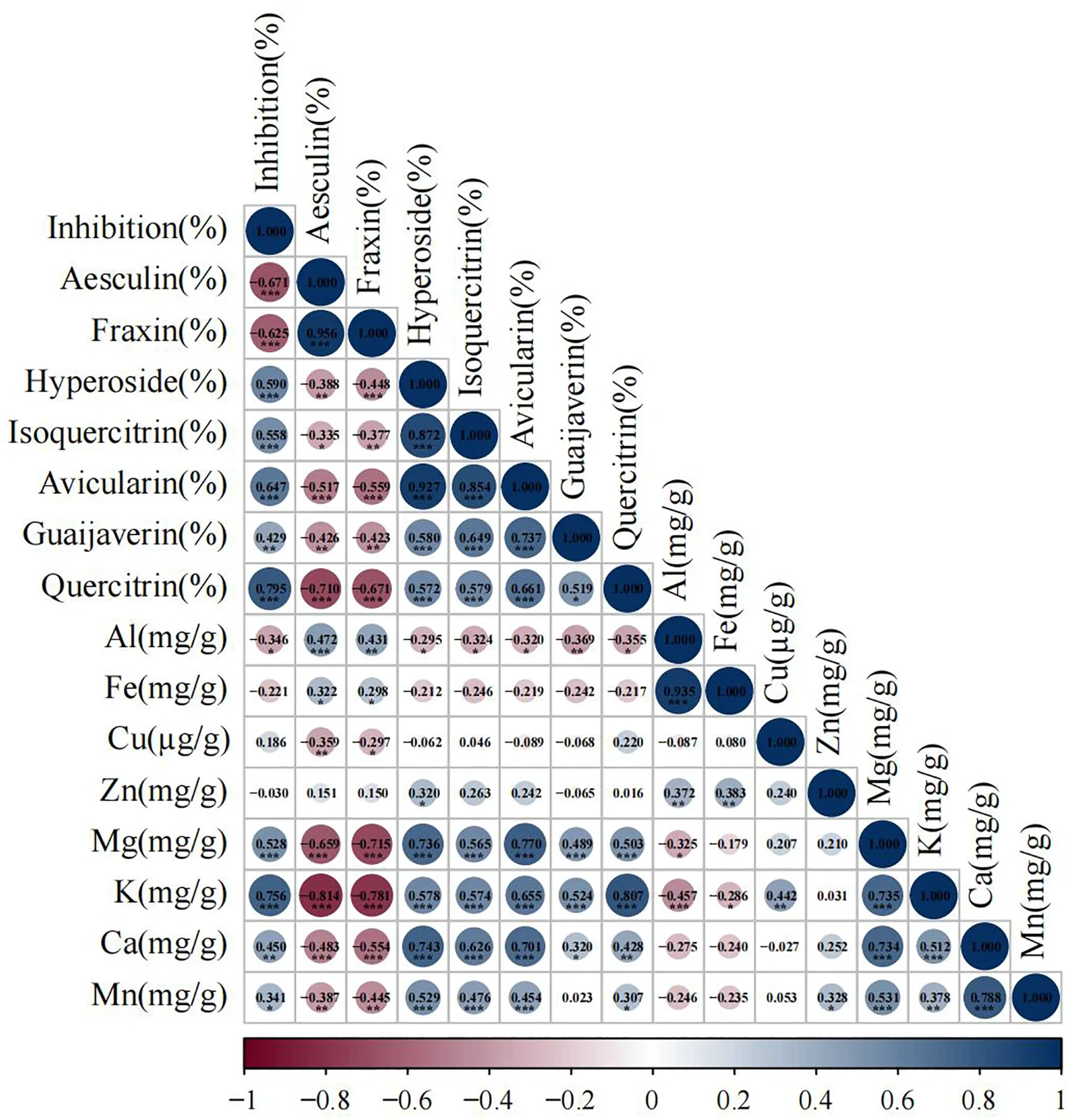
Correlation analysis of multi-component contents, metal element contents, and inhibition rate against bovine serum albumin (BSA) denaturation in 51 batches of *R. tomentosum* samples.The heatmap shows Spearman correlation coefficients. Blue and red colors indicate positive and negative correlations, respectively. *p < 0.05, **p < 0.01, ***p < 0.001.

The correlation analysis results among metal element contents, multi -components contents, and anti-BSA denaturation activity in different parts of *R. tomentosum* are shown in **Figure 9**.

The two coumarins (aesculin and fraxin) were significantly positively correlated with each other (r=0.956), and the five flavonoid glycosides (hyperoside, isoquercitrin, avicularin, guaijaverin, and quercitrin) also exhibited significant positive intercorrelations, with correlation coefficients ranging from 0.572 to 0.927, These results suggest synergistic accumulation of compounds within each class. However, a significant negative correlation was observed between the coumarins and the flavonoid glycosides, indicating a competitive relationship between these two classes of compounds.

Significant positive correlations were observed between Al and Fe (*r* = 0.935), as well as between Al and Cu. Mn, Mg, K, and Ca were significantly positively correlated with each other, with correlation coefficients ranging from 0.378 to 0.788. However, K was significantly negatively correlated with Al and Fe, whereas Zn showed significant positive correlations with Al and Fe. With respect to the bioactive constituents, Al and Fe were significantly positively correlated with the coumarins (aesculin and fraxin) but significantly negatively correlated with the flavonoid glycosides (isoquercitrin, avicularin, guaijaverin, and quercitrin). Mg, K, Ca, and Mn exhibited significant negative correlations with coumarins, yet significant positive correlations with flavonoid glycosides. Additionally, Cu displayed significant negative correlations with the coumarins (aesculin and fraxin).

Regarding the anti-BSA denaturation inhibition rate, aesculin and fraxin exhibited significant negative correlations, while hyperoside, isoquercitrin, avicularin, guaijaverin, and quercitrin showed significant positive correlations. These results suggest that flavonoid glycosides may be the principal bioactive components responsible for the anti-inflammatory activity of *R. tomentosum*, whereas the coumarins showed limited or even opposing contributions to the activity under the present experimental conditions.

### 3.9 Anti-BSA denaturation (anti-inflammatory) results of multiple components and vitamin C

**Figure 10:**
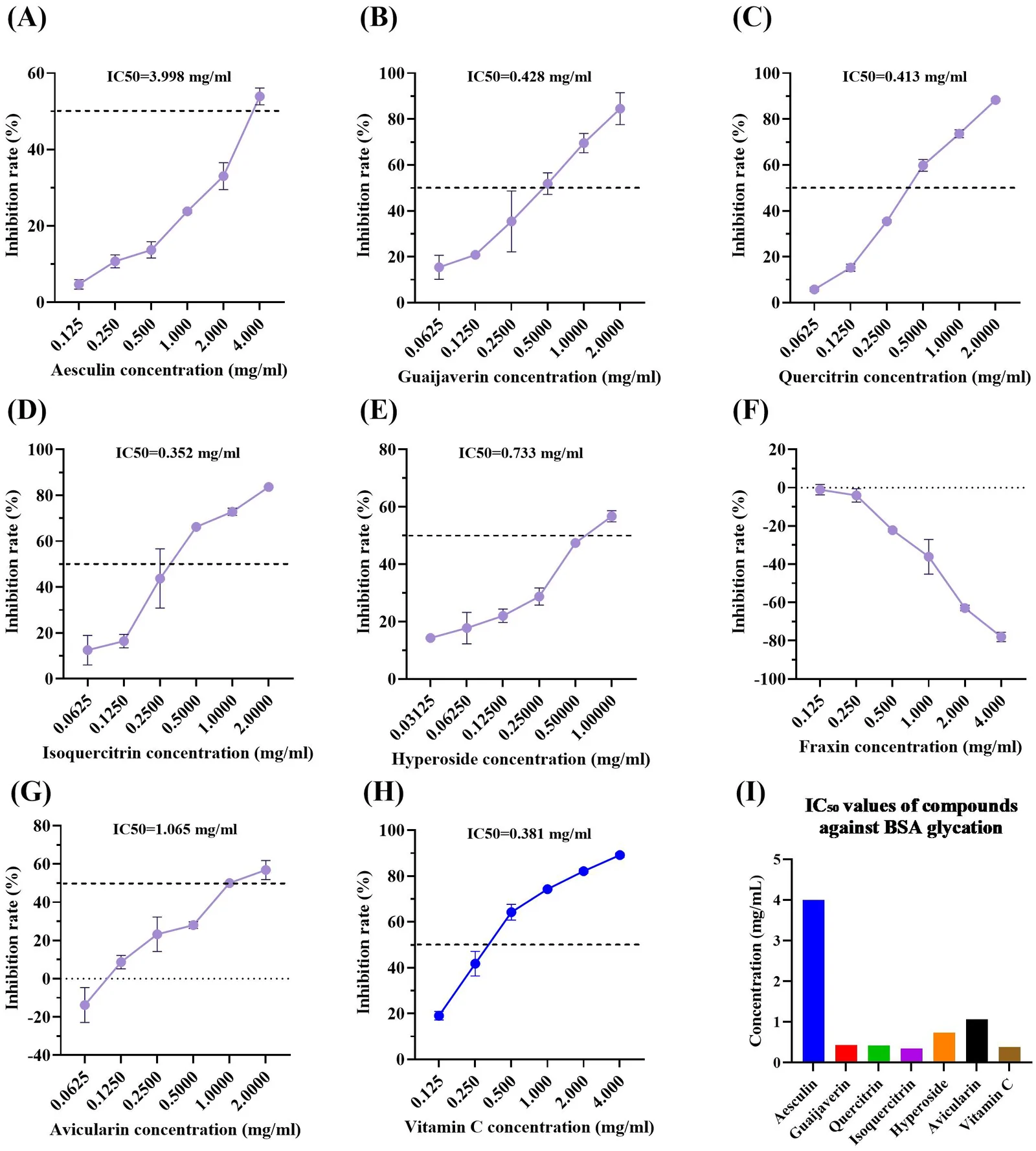
Dose–response curves and IC_50_ comparison of seven compounds and vitamin C against-BSA denaturation. Data are presented as mean ± SD (n = 3), Vitamin C was used as a positive control. Panels **(A–H)** show the dose–response curves of aesculin, guaijaverin, quercitrin, isoquercitrin, hyperoside, fraxin, avicularin, and vitamin C, respectively. Panel **(I)** shows the comparison of IC_50_ values.Under the experimental conditions of this study, all five flavonoid glycosides exhibited anti-BSA denaturation activities that were partially superior to or comparable to that of vitamin C. Among them, quercitrin (IC_50_ = 0.413 mg/mL) and isoquercitrin (IC_50_ = 0.352 mg/mL) showed IC_50_ values comparable to that of vitamin C (0.381 mg/mL), indicating that they are potent anti-protein denaturation agents. In contrast, the coumarins displayed virtually no anti-BSA denaturation activity: aesculin showded extremely weak activity (IC_50_ = 3.998 mg/mL), while fraxin even exhibited negative inhibition rates at high concentrations. These findings are consistent with the previous correlation analysis, which revealed that coumarins were significantly negatively correlated with the inhibition rate, whereas flavonoid glycosides were significantly positively correlated with the inhibition rate.

The dose–response curves and IC_50_ values of the seven compounds and vitamin C are presented in **Figure 10**.

The differences in activity among the flavonoid glycosides may be attributed to the types of sugar moieties and their linkage positions. Specifically, compounds substituted with glucosyl (isoquercitrin) and rhamnosyl (quercitrin) groups exhibited the strongest activities, while those with arabinosyl (guaijaverin and avicularin) substitutions showed relatively weaker activities. Collectively, these results suggest that flavonoid glycosides are likely the principal bioactive components responsible for the anti-BSA denaturation activity of *R. tomentosum*.

## 4 Discussion

### 4.1 *R. tomentosum* may serve as a candidate woody species for future mechanism studies on coumarin-mediated iron absorption

Recent studies have revealed that coumarins, as secondary metabolites, play a key role in promoting iron solubilization and/or uptake in non-graminaceous species (Tsai and Schmidt, 2017). Among them, fraxetin and sideretin are the primary coumarins involved in iron acquisition. In the present study, we detected three coumarins—aesculin, fraxin, and fraxetin—across all parts of *R. tomentosum* from various geographical origins (as confirmed by reference standards in Section 3.4). While sideretin was not detected in our samples, the presence of fraxetin is consistent with its reported role in iron mobilization. Coumarins are synthesized in the roots, where they also influence the root-associated microbiota. Under conditions of iron deficiency, cold stress, or pathogen infection, coumarins are translocated from the roots to the shoots and accumulate there (Robe et al., 2021). The results of the present study demonstrated that the coumarins aesculin, fraxetin, and fraxin were distributed across all different parts of *R. tomentosum* from various geographical origins, indicating high mobility. *R. tomentosum* is a circum-Arctic dwarf shrub; to adapt to cold environments, coumarins are likely synthesized in the roots and subsequently translocated and accumulated in old and tender branches. Consequently, coumarin contents were higher in the roots, old branches, and young branches than in other parts.

*In vitro* assays and chemical complementation experiments have shown that coumarin-mediated ferric reduction is effective only under acidic conditions (Paffrath et al., 2024). In the present study, iron content in different parts of *R. tomentosum* showed significant positive correlations with aesculin and fraxin, with correlation coefficients of 0.322 and 0.298, respectively. Iron was predominantly accumulated in the roots across different geographical origins, indicating a positive correlation between coumarin secretion/synthesis and iron accumulation. All *R. tomentosum* samples collected in this study originated from acidic soils (Tang et al., 2020; Zhou et al., 2023; Yin et al., 2025). These findings are consistent with molecular studies on coumarin-mediated iron uptake in *Arabidopsis* (Paffrath et al., 2024), suggesting that *R. tomentosum* may serve as a candidate woody species for future mechanistic studies on coumarin-mediated iron mobilization. However, it should be emphasized that this inference is primarily derived from correlation analyses of metabolomics data and does not establish causality. Direct experimental validation—such as root exudate analysis under varying Fe concentrations, iron uptake assays using purified coumarin compounds, or targeted gene expression studies of coumarin biosynthetic genes (*F6’H1, S8H, CYP82C4*) and transporter genes (*PDR9*)—is required to confirm the specific roles of coumarins in iron acquisition in *R. tomentosum*.

### 4.2 High co-accumulation of Al and Fe and their synergistic effects with Mg and K on secondary metabolites in *R. tomentosum*

Studies have shown that under acidic growth conditions, Fe and Al often co-exist at high levels and may jointly influence the chemical composition and quality-related traits of tea leaves (Li et al., 2026). In the present study, Al and Fe contents showed a significant strong positive correlation across different parts of *R. tomentosum* (r = 0.935). Consistent with Fe, Al content was also higher in roots, indicating that in acidic soils, Fe and Al are synchronously absorbed at high levels by the root system. Leaves collected from the same geographical origin, year, and month as the corresponding roots also contained high levels of Fe and Al, as observed in *Ledum palustre* var. *dilatatum* and *L. palustre* var. *palustre* from Changbai Mountain, *L. palustre* var. *palustre* from Genhe (Greater Khingan Mountains), and *L. palustre* var. *palustre* from Yichun (Lesser Khingan Mountains). The Fe and Al contents ranged from 138 to 1, 715 mg/kg and from 129 to 1, 344 mg/kg, respectively. In contrast, samples from Tahe and Mohe (Greater Khingan Mountains) showed lower contents, which may be related to soil acidity. Among the six geographical origins, three exhibited high Fe and Al contents in leaves, suggesting that *R. tomentosum* may be one of the aluminum-tolerant species within the genus *Rhododendron* (Zhang et al., 2024; Li et al., 2025), capable of accumulating high concentrations of Al while regulating Fe uptake.

Flavonoids, a class of compounds that includes flavones, are synthesized exclusively in plants (Bozzo and Unterlander, 2021). They are generally present in terminal tissues such as flowers and fruits but are also subject to turnover within the plant. In the present study, flavonoid glycosides such as hyperoside and isoquercitrin were detected not only in aerial parts but also in the roots of the dwarf shrub *R. tomentosum*, indicating a certain degree of mobility across different plant parts. In terms of the contents of flavonoid glycosides, leaves harvested in June and September contained relatively higher levels, while those collected in July and August had comparatively lower levels. This seasonal variation may be explained by the redirection of flavonoid biosynthesis toward flowers and fruits during the latter period.

Recent studies have shown that Al promotes the accumulation of flavone/flavonol glycosides (Lai et al., 2025; Jiang et al., 2023; Kováčik et al., 2012) but reduces the levels of coumarin-related compounds (Kováčik et al., 2012). In the present study, however, Al content showed significant negative correlations with flavonoid glycosides (r = −0.295 to −0.369) and significant positive correlations with coumarins (r = 0.431 to 0.472) in different parts of *R. tomentosum*. These results appear to differ from previous reports on the effects of Al alone. This discrepancy may be attributable to the high co-accumulation of Fe and Al in our samples (r = 0.935), potentially leading to synergistic or antagonistic interactions that are not captured in single-element studies. We tentatively propose that the co-occurrence of Fe and Al may preferentially drive the phenylpropanoid flux toward the coumarin branch by consuming the common precursor 4-coumaroyl-CoA, thereby inhibiting metabolic flux toward the flavonoid glycoside branch (Vogt, 2010).

In contrast, Mg and K showed significant positive correlations with flavonoid glycosides (r = 0.489 to 0.807) and significant negative correlations with coumarins (r = −0.659 to −0.814). It has been reported that Mg supply levels can significantly influence the differential expression of flavonoids and coumarins in plants, and the underlying mechanism may be related to its role in affecting photosynthetic efficiency and serving as a cofactor for key enzymes in the phenylpropanoid pathway (Jin et al., 2023). Additionally, K deficiency has been shown to reduce leaf net photosynthetic rate and decrease the activity of 4-coumarate-CoA ligase (4CL), a key enzyme, ultimately leading to reduced contents of flavonoids and chlorogenic acid (Liu et al., 2011). These findings are consistent with our observations, further supporting the speculation that Mg and K may promote flavonoid glycoside accumulation while suppressing coumarin levels, opposite to the proposed Al-Fe synergistic effect.

However, the proposed synergistic mechanism of Al and Fe, as well as the opposing regulatory roles of Mg and K, are currently based solely on correlation evidence and require further experimental validation through controlled treatments with varying element concentrations, combined with transcriptomic or genetic approaches. It should be emphasized that these hypotheses should be interpreted with caution and warrant direct testing in future studies using controlled experimental systems.

### 4.3 Isoquercitrin, quercitrin, guaijaverin, hyperoside, and avicularin may be major potential active components for anti-BSA activity

In this study, seven relatively stable and readily available coumarin and flavonoid glycoside compounds—namely aesculin, fraxin, hyperoside, isoquercitrin, avicularin, guaijaverin, and quercitrin—were selected from ten potential quality-discriminating characteristic components of different *R. tomentosum* samples for content correlation analysis. The results revealed that within each class, coumarins and flavonoid glycosides exhibited significant positive correlations, with correlation coefficients ranging from 0.519 to 0.956. In contrast, significant negative correlations were observed between the two classes, with correlation coefficients ranging from 0.355 to 0.710. Correlation analysis between the inhibition rate against BSA denaturation and the contents of the extracts from different parts of *R. tomentosum* showed that the inhibition rate was significantly negatively correlated with the contents of coumarins (correlation coefficients of −0.625 and −0.671, respectively), while significantly positively correlated with the contents of flavonoid glycosides (correlation coefficients ranging from 0.429 to 0.795). These findings suggested that coumarins and flavonoid glycosides may exert different effects on the inhibition of BSA denaturation.

Further experiments were conducted by testing the seven compounds and the positive control (vitamin C) at various concentrations against BSA denaturation. The results showed that fraxin exhibited a negative inhibition rate across the concentration range of 0.125 mg/mL to 4 mg/mL, with the inhibition rate decreasing as the concentration increased. Aesculin showed an IC_50_ value of 3.998 mg/mL. The IC_50_ values of isoquercitrin, vitamin C, quercitrin, guaijaverin, hyperoside, and avicularin increased in the following order: isoquercitrin (0.352 mg/mL) < vitamin C (0.381 mg/mL) < quercitrin (0.413 mg/mL) < guaijaverin (0.428 mg/mL) < hyperoside (0.733 mg/mL) < avicularin (1.065 mg/mL). with isoquercitrin and quercitrin exhibiting IC_50_ values close to that of vitamin C (within a 10% difference). These results were highly consistent with the positive and negative correlation patterns observed between the inhibition rates of extracts from different parts of *R. tomentosum* and the contents of the respective compounds. Therefore, we speculate that isoquercitrin, quercitrin, guaijaverin, hyperoside, and avicularin may be the major potential active components contributing to the anti-BSA denaturation activity of the extracts from different parts of *R. tomentosum*.

However, the inhibition rates exhibited by different parts of *R. tomentosum* and their correlations with flavonoid glycosides were neither simply the sum of the inhibition rates of each potential active component nor did they show a consistent correlation with the inhibition rates of these seven compounds individually. This may be attributed to the complexity of the plant’s chemical composition and the potential interference among its various internal components.

### 4.4 Synergistic identification of former *Ledum* species using DNA barcoding (ITS sequences) and flavonoid profiles

*R. tomentosum* was formerly classified under the genus *Ledum* but is now placed within subsection *Ledum* of subgenus *Rhododendron*. This subsection comprises 4–9 species (Kron, 1997; Kihlman, 2004), including *R. subulatum* (Harmaja, 2002), *R. diversipilosum* (Harmaja, 1999), *R. tomentosum*, *R. tolmachevii*, *R. hypoleucum*, *R. subarcticum*, *R. neoglandulosum* (Harmaja, 1991), *R. groenlandicum* (Harmaja, 1990), and *R. columbianum* (Singh et al., 2021), with *R. columbianum* possibly being a natural hybrid (Kihlman, 2004). Plants of subsection *Ledum* are circum-Arctic in distribution, exhibiting continuous morphological variation across geographical ranges, which makes species identification challenging, particularly when samples are in the form of dried powder.

In 1990, Kron and Judd (1990) and Harmaja (1990) proposed the transfer of the genus *Ledum* into *Rhododendron* and revised the nomenclature of the plants within subsection *Ledum*. Subsequently, Lantai and Kihlman (1995), Kron (1997); Goetsch et al., 2005, and Hart et al., 2017 analyzed *Ledum* and related species in the same subsection from DNA molecular perspectives, including chromosomes, *matK* sequences, ITS sequences, *RPB2* gene sequences, nuclear genes, and chloroplasts, all providing strong evidence supporting the inclusion of former *Ledum* species into *Rhododendron*. These studies mainly focused on subsection *Ledum* plants from North America and Europe, as well as Chinese *Ledum palustre* var. *palustre*. However, no DNA barcoding study on Chinese *Ledum palustre* var. *dilatatum* has been reported to date, despite DNA barcoding having been widely used for plant identification (Graper et al., 2021; Cetiz et al., 2023).

As mentioned earlier, transitions were observed at positions 84, 94, and 176 of the ITS sequences of *L. palustre* var. *palustre* and *L. palustre* var. *dilatatum*. In the phylogenetic tree, *L. palustre* var. *dilatatum* clustered into a small branch with KY356325.1 R.tomentosum and MT779112.1 R.tomentosum, while *L. palustre* var. *palustre* (except for KY356326.1 *R. columbianum*) clustered into a small branch with species of subsection *Ledum* and *R. ferrugineum* (subsection *Rhododendron*), with relatively low bootstrap support (51%).

*L. palustre* var. *dilatatum* leaves and branches contained high levels of peak RT83 (identified as 3-[(3, 6-di-O-acetyl-β-D-galactopyranosyl)oxy]-2-(3, 4-dihydroxyphenyl)-5, 7-dihydroxy-4H-1-benzopyran-4-one; **Supplementary Figure 1**-**S4**), which was not detected in *L. palustre* var. *palustre* across different geographical origins and plant parts, suggesting that this compound could serve as a flavonoid marker to distinguish the two varieties.

This study proposes a synergistic approach combining DNA barcoding and chemical composition to identify species within subsection *Ledum*, aiming to address the long-standing challenge of species delimitation in this group. This study addresses the research gap regarding the ITS sequences and flavonoid profiles of Chinese *L. palustre* var. *dilatatum* and *L. palustre* var. *palustre*. However, a limitation of this study is that *L. palustre* var. *dilatatum* (broad-leaved type) samples were collected from only one origin (Changbai Mountain); thus, more samples from additional origins are needed for validation. Future studies should include a larger number of broad-leaved type samples from multiple geographical locations to confirm the reliability of the ITS sequence and flavonoid markers proposed in this study.

### 4.5 Limitations and future perspectives

It should be acknowledged that the sample size of the flower group (n = 3) in this study was considerably smaller than that of the leaf group (n = 21). This imbalance may have potentially influenced the model performance. Although the customized 7-fold cross-validation (Q² = 0.855) and permutation tests (Q² intercept = –0.49) confirmed the model’s predictive reliability and ruled out overfitting, the small sample size of the flower group still limits the representativeness of the metabolic profile for this tissue type. Therefore, interpretations of metabolic differences involving the flower group should be made with caution. Future studies with expanded sample sizes for flowers and other underrepresented tissues are warranted to further validate the present findings and enhance the robustness of our conclusions.

## 5 Conclusion

Based on the above results and discussion, this study provides novel insights into the intrinsic relationships among “plant part – chemical composition (secondary metabolites and metal elements) – bioactivity” in Chinese *R.tomentosum* from the perspectives of ITS sequences, metal elements in different plant parts, secondary metabolites, and anti-BSA denaturation activity. The findings are summarized as follows:

Coumarins were distributed across different parts of *R. tomentosum* from various geographical origins, with higher contents in roots, old branches, and young branches. Their contents showed significant positive correlations with iron content, suggesting that *R. tomentosum* may serve as a candidate woody species for future mechanistic studies on coumarin-mediated iron mobilization. However, it should be emphasized that this inference is based on correlation analyses, and direct experimental validation—such as root exudate analysis, iron uptake assays, and targeted gene expression studies—is required to establish causality.

Aluminum (Al) and Fe showed significant positive correlations across different parts of *R. tomentosum* from various origins, and both were significantly positively correlated with coumarins. In contrast, Al showed significant negative correlations with flavonoid glycosides. These results indicate that Al and Fe are highly co-accumulated and may synergistically influence the accumulation of secondary metabolites in different parts of *R. tomentosum*. Nevertheless, this proposed synergistic mechanism is currently supported only by correlation evidence and warrants further experimental validation through controlled treatments with varying Fe/Al concentrations, combined with transcriptomic or genetic approaches.

Based on correlation analyses between BSA denaturation inhibition rates of different plant parts and single-compound relatively contents, as well as dose-response experiments, hyperoside, avicularin, guaijaverin, isoquercitrin, and quercitrin were identified as the major potential bioactive components responsible for the anti-BSA denaturation activity of *R. tomentosum.* It should be noted that the anti-inflammatory activity was evaluated solely by the BSA denaturation inhibition assay; additional assays (e.g., NO production inhibition in LPS-stimulated macrophages) are needed to further confirm the anti-inflammatory potential of these compounds.

The presence or absence of the compound 3-[(3, 6-di-O-acetyl-β-D-galactopyranosyl)oxy]-2-(3, 4-dihydroxyphenyl)-5, 7-dihydroxy-4H-1-benzopyran-4-one, together with the transitions at positions 84, 94, and 176 of the ITS sequences, represent the main differences between the leaves of *Ledum palustre* var. *dilatatum* and *L. palustre* var. *palustre*. The combination of these two features may enable the discrimination of Chinese *L. palustre* var. *dilatatum* and *L. palustre* var. *palustre*. This synergistic identification strategy provides a solution to the challenging taxonomic delimitation of species within subsection *Ledum* and directly fills the research gap regarding the ITS sequences and flavonoid profiles of Chinese *L. palustre* var. *dilatatum* and *L. palustre* var. *palustre*.

In summary, while the present study generates testable hypotheses regarding coumarin-iron interactions, Al-Fe synergistic effects, and bioactive components, these findings should be considered hypothesis-generating rather than definitive. Future mechanistic studies integrating transcriptomics, proteomics, and targeted metabolomics, combined with controlled experiments and functional assays, are essential to validate the proposed mechanisms and to fully elucidate the ecological significance of metal–metabolite interactions in this species. These results may facilitate the artificial cultivation, selective breeding, and comprehensive utilization of *R. tomentosum* in future practices.

## Data Availability

The datasets presented in this study can be found in online repositories. The names of the repository/repositories and accession number(s) can be found in the article/supplementary material.
