# Supplementary material for: Correlation analysis of secondary metabolites, metal elements, and anti-BSA denaturation activity in different parts of Rhododendron tomentosum from China
Source: Front Plant Sci. 2026 Aug 14;17:1900896. doi: 10.3389/fpls.2026.1900896 (PMC13522219; doi:10.3389/fpls.2026.1900896)
Supplement: Supplementary file 1 [file DataSheet1.docx]

**Supplementary Table S1.** **Eighty-six peak areas of 51 batches of *Rhododendron tomentosum* from different origins.**

| **Primary ID** | **RT1** | **RT2** | **RT3** | **RT4** | **RT5** | **RT6** | **RT7** | **RT8** | **RT9** | **RT10** | **RT11** | **RT12** | **RT13** | **RT14** | **RT15** | **RT16** | **RT17** | **RT18** |
| --- | --- | --- | --- | --- | --- | --- | --- | --- | --- | --- | --- | --- | --- | --- | --- | --- | --- | --- |
| S1 | 0.000 | 0.000 | 0.000 | 0.000 | 0.000 | 0.000 | 0.000 | 0.000 | 0.000 | 512.835 | 0.000 | 13.979 | 0.000 | 0.000 | 75.851 | 0.000 | 0.000 | 0.000 |
| S2 | 0.000 | 0.000 | 0.000 | 0.000 | 0.000 | 0.000 | 0.000 | 0.000 | 0.000 | 501.328 | 0.000 | 14.007 | 0.000 | 0.000 | 87.032 | 0.000 | 0.000 | 0.000 |
| S3 | 0.000 | 0.000 | 0.000 | 0.000 | 0.000 | 0.000 | 0.000 | 0.000 | 0.000 | 510.153 | 0.000 | 11.334 | 0.000 | 0.000 | 58.198 | 0.000 | 0.000 | 0.000 |
| S4 | 0.000 | 0.000 | 0.000 | 0.000 | 0.000 | 0.000 | 0.000 | 0.000 | 0.000 | 387.045 | 0.000 | 0.000 | 0.000 | 0.000 | 72.191 | 0.000 | 0.000 | 0.000 |
| S5 | 0.000 | 0.000 | 0.000 | 0.000 | 0.000 | 0.000 | 0.000 | 0.000 | 0.000 | 286.111 | 0.000 | 0.000 | 0.000 | 0.000 | 61.841 | 0.000 | 0.000 | 0.000 |
| S6 | 0.000 | 0.000 | 0.000 | 0.000 | 0.000 | 0.000 | 0.000 | 0.000 | 12.087 | 501.261 | 0.000 | 17.162 | 0.000 | 0.000 | 65.534 | 0.000 | 0.000 | 0.000 |
| S7 | 0.000 | 0.000 | 0.000 | 0.000 | 0.000 | 0.000 | 0.000 | 0.000 | 0.000 | 577.540 | 0.000 | 14.093 | 0.000 | 0.000 | 144.724 | 0.000 | 0.000 | 0.000 |
| S8 | 0.000 | 0.000 | 0.000 | 0.000 | 0.000 | 0.000 | 0.000 | 0.000 | 0.000 | 533.227 | 0.000 | 11.947 | 0.000 | 0.000 | 102.308 | 0.000 | 0.000 | 0.000 |
| S9 | 0.000 | 0.000 | 0.000 | 0.000 | 0.000 | 0.000 | 0.000 | 0.000 | 0.000 | 521.647 | 0.000 | 11.900 | 0.000 | 0.000 | 79.495 | 0.000 | 0.000 | 0.000 |
| S10 | 0.000 | 0.000 | 0.000 | 0.000 | 0.000 | 0.000 | 0.000 | 0.000 | 0.000 | 438.924 | 0.000 | 10.461 | 0.000 | 0.000 | 97.181 | 0.000 | 0.000 | 0.000 |
| S11 | 0.000 | 0.000 | 0.000 | 0.000 | 0.000 | 0.000 | 0.000 | 0.000 | 0.000 | 394.747 | 0.000 | 10.038 | 0.000 | 0.000 | 95.978 | 0.000 | 0.000 | 0.000 |
| S12 | 0.000 | 0.000 | 0.000 | 0.000 | 0.000 | 0.000 | 0.000 | 0.000 | 0.000 | 566.872 | 0.000 | 15.254 | 0.000 | 0.000 | 69.935 | 0.000 | 0.000 | 0.000 |
| S13 | 0.000 | 0.000 | 0.000 | 0.000 | 0.000 | 0.000 | 0.000 | 0.000 | 0.000 | 389.300 | 0.000 | 0.000 | 0.000 | 0.000 | 144.528 | 0.000 | 0.000 | 0.000 |
| S14 | 0.000 | 0.000 | 0.000 | 0.000 | 0.000 | 0.000 | 0.000 | 0.000 | 0.000 | 364.553 | 0.000 | 0.000 | 12.538 | 13.517 | 178.276 | 0.000 | 0.000 | 0.000 |
| S15 | 0.000 | 0.000 | 0.000 | 0.000 | 0.000 | 0.000 | 0.000 | 0.000 | 0.000 | 479.475 | 0.000 | 0.000 | 14.159 | 35.159 | 156.384 | 0.000 | 0.000 | 0.000 |
| S16 | 0.000 | 0.000 | 0.000 | 0.000 | 0.000 | 0.000 | 0.000 | 0.000 | 0.000 | 431.122 | 0.000 | 0.000 | 10.382 | 0.000 | 144.188 | 0.000 | 0.000 | 0.000 |
| S17 | 10.924 | 10.200 | 0.000 | 0.000 | 0.000 | 0.000 | 0.000 | 0.000 | 0.000 | 419.724 | 0.000 | 0.000 | 19.534 | 0.000 | 145.409 | 0.000 | 0.000 | 0.000 |
| S18 | 0.000 | 0.000 | 0.000 | 0.000 | 0.000 | 0.000 | 0.000 | 0.000 | 0.000 | 404.041 | 0.000 | 10.683 | 0.000 | 0.000 | 69.842 | 0.000 | 0.000 | 0.000 |
| S19 | 0.000 | 0.000 | 0.000 | 0.000 | 0.000 | 0.000 | 205.289 | 0.000 | 14.584 | 123.358 | 0.000 | 0.000 | 13.765 | 0.000 | 195.858 | 27.521 | 21.879 | 0.000 |
| S20 | 0.000 | 0.000 | 0.000 | 0.000 | 0.000 | 17.879 | 101.397 | 0.000 | 11.031 | 111.463 | 0.000 | 0.000 | 37.212 | 0.000 | 211.716 | 19.719 | 0.000 | 0.000 |
| S21 | 0.000 | 20.896 | 0.000 | 0.000 | 0.000 | 10.527 | 179.323 | 0.000 | 0.000 | 235.672 | 0.000 | 0.000 | 50.013 | 45.994 | 231.368 | 0.000 | 0.000 | 0.000 |
| S22 | 18.682 | 14.021 | 0.000 | 0.000 | 0.000 | 20.798 | 319.613 | 0.000 | 0.000 | 169.571 | 0.000 | 11.021 | 105.700 | 34.832 | 241.106 | 0.000 | 0.000 | 0.000 |
| S23 | 10.314 | 15.761 | 0.000 | 0.000 | 18.700 | 0.000 | 275.348 | 0.000 | 13.255 | 120.314 | 0.000 | 0.000 | 38.975 | 0.000 | 246.922 | 12.893 | 0.000 | 0.000 |
| S24 | 0.000 | 0.000 | 0.000 | 0.000 | 0.000 | 13.313 | 58.925 | 0.000 | 0.000 | 120.896 | 0.000 | 0.000 | 19.121 | 0.000 | 131.146 | 0.000 | 0.000 | 0.000 |
| S25 | 11.827 | 0.000 | 0.000 | 0.000 | 0.000 | 0.000 | 169.507 | 0.000 | 0.000 | 76.749 | 0.000 | 0.000 | 71.940 | 13.776 | 292.111 | 12.565 | 0.000 | 10.890 |
| S26 | 0.000 | 0.000 | 0.000 | 0.000 | 0.000 | 0.000 | 23.130 | 0.000 | 0.000 | 16.725 | 0.000 | 0.000 | 10.096 | 0.000 | 55.642 | 0.000 | 0.000 | 0.000 |
| S27 | 0.000 | 0.000 | 0.000 | 0.000 | 0.000 | 0.000 | 28.011 | 0.000 | 0.000 | 38.456 | 0.000 | 0.000 | 29.873 | 0.000 | 72.141 | 0.000 | 0.000 | 0.000 |
| S28 | 0.000 | 0.000 | 0.000 | 0.000 | 11.798 | 0.000 | 506.538 | 0.000 | 25.609 | 89.338 | 0.000 | 0.000 | 47.306 | 42.113 | 602.297 | 26.042 | 14.250 | 0.000 |
| S29 | 0.000 | 0.000 | 0.000 | 0.000 | 10.783 | 0.000 | 442.109 | 0.000 | 21.310 | 81.863 | 0.000 | 0.000 | 47.946 | 47.179 | 337.440 | 21.563 | 14.343 | 0.000 |
| S30 | 0.000 | 0.000 | 0.000 | 0.000 | 0.000 | 0.000 | 272.127 | 0.000 | 19.536 | 129.762 | 0.000 | 0.000 | 75.012 | 46.520 | 433.390 | 30.431 | 11.222 | 0.000 |
| S31 | 0.000 | 0.000 | 0.000 | 0.000 | 0.000 | 0.000 | 88.765 | 0.000 | 0.000 | 17.654 | 0.000 | 0.000 | 10.367 | 0.000 | 120.083 | 21.396 | 18.526 | 15.261 |
| S32 | 0.000 | 0.000 | 0.000 | 0.000 | 0.000 | 0.000 | 32.759 | 0.000 | 0.000 | 16.627 | 0.000 | 0.000 | 14.238 | 0.000 | 53.347 | 0.000 | 0.000 | 0.000 |
| S33 | 0.000 | 0.000 | 0.000 | 0.000 | 0.000 | 0.000 | 29.864 | 0.000 | 0.000 | 23.724 | 0.000 | 0.000 | 30.875 | 0.000 | 56.224 | 0.000 | 0.000 | 0.000 |
| S34 | 0.000 | 11.146 | 0.000 | 0.000 | 17.876 | 0.000 | 296.785 | 0.000 | 13.112 | 40.082 | 0.000 | 0.000 | 46.389 | 0.000 | 239.646 | 10.157 | 0.000 | 0.000 |
| S35 | 13.946 | 10.844 | 0.000 | 0.000 | 0.000 | 0.000 | 97.458 | 0.000 | 0.000 | 45.487 | 0.000 | 0.000 | 14.436 | 0.000 | 116.649 | 0.000 | 0.000 | 0.000 |
| S36 | 0.000 | 0.000 | 0.000 | 0.000 | 0.000 | 0.000 | 38.958 | 0.000 | 0.000 | 14.699 | 0.000 | 0.000 | 0.000 | 0.000 | 53.113 | 0.000 | 0.000 | 0.000 |
| S37 | 10.205 | 0.000 | 0.000 | 0.000 | 0.000 | 11.651 | 74.752 | 0.000 | 0.000 | 44.803 | 0.000 | 0.000 | 24.186 | 0.000 | 121.034 | 0.000 | 0.000 | 0.000 |
| S38 | 0.000 | 13.585 | 0.000 | 0.000 | 0.000 | 0.000 | 184.099 | 0.000 | 0.000 | 46.775 | 0.000 | 0.000 | 36.166 | 47.110 | 174.571 | 0.000 | 0.000 | 0.000 |
| S39 | 18.703 | 22.386 | 0.000 | 0.000 | 0.000 | 0.000 | 141.308 | 0.000 | 0.000 | 110.574 | 0.000 | 0.000 | 58.095 | 0.000 | 207.512 | 0.000 | 0.000 | 0.000 |
| S40 | 0.000 | 0.000 | 0.000 | 0.000 | 0.000 | 0.000 | 0.000 | 0.000 | 0.000 | 10.080 | 0.000 | 0.000 | 0.000 | 0.000 | 13.086 | 0.000 | 0.000 | 0.000 |
| S41 | 0.000 | 0.000 | 0.000 | 0.000 | 0.000 | 0.000 | 42.030 | 0.000 | 0.000 | 27.420 | 0.000 | 0.000 | 10.799 | 0.000 | 74.842 | 0.000 | 0.000 | 0.000 |
| S42 | 19.243 | 20.215 | 0.000 | 0.000 | 0.000 | 0.000 | 341.373 | 0.000 | 0.000 | 100.599 | 0.000 | 12.888 | 125.680 | 70.145 | 323.639 | 0.000 | 0.000 | 0.000 |
| S43 | 0.000 | 21.149 | 11.928 | 13.821 | 14.812 | 0.000 | 301.950 | 10.337 | 0.000 | 126.805 | 26.370 | 41.063 | 265.915 | 104.316 | 218.651 | 47.324 | 24.386 | 0.000 |
| S44 | 0.000 | 17.366 | 0.000 | 0.000 | 0.000 | 0.000 | 201.266 | 0.000 | 28.869 | 25.298 | 0.000 | 0.000 | 39.261 | 0.000 | 343.732 | 30.171 | 0.000 | 0.000 |
| S45 | 0.000 | 29.560 | 0.000 | 0.000 | 0.000 | 0.000 | 291.273 | 0.000 | 21.340 | 77.917 | 0.000 | 0.000 | 21.281 | 42.555 | 221.103 | 35.208 | 24.982 | 0.000 |
| S46 | 0.000 | 0.000 | 0.000 | 0.000 | 0.000 | 0.000 | 11.537 | 0.000 | 0.000 | 8.754 | 0.000 | 0.000 | 0.000 | 0.000 | 46.549 | 0.000 | 0.000 | 0.000 |
| S47 | 0.000 | 0.000 | 0.000 | 0.000 | 0.000 | 0.000 | 0.000 | 0.000 | 0.000 | 6.285 | 0.000 | 0.000 | 0.000 | 0.000 | 47.215 | 0.000 | 0.000 | 0.000 |
| S48 | 0.000 | 0.000 | 0.000 | 0.000 | 0.000 | 0.000 | 0.000 | 0.000 | 0.000 | 8.978 | 0.000 | 0.000 | 0.000 | 0.000 | 24.833 | 0.000 | 0.000 | 0.000 |
| S49 | 0.000 | 0.000 | 0.000 | 0.000 | 0.000 | 0.000 | 17.151 | 0.000 | 0.000 | 27.157 | 0.000 | 0.000 | 0.000 | 0.000 | 196.404 | 0.000 | 0.000 | 0.000 |
| S50 | 0.000 | 0.000 | 0.000 | 0.000 | 0.000 | 0.000 | 34.371 | 0.000 | 0.000 | 64.469 | 0.000 | 15.818 | 36.607 | 20.043 | 290.771 | 0.000 | 0.000 | 0.000 |
| S51 | 0.000 | 0.000 | 0.000 | 0.000 | 0.000 | 0.000 | 20.462 | 0.000 | 0.000 | 65.725 | 0.000 | 0.000 | 41.992 | 0.000 | 213.337 | 0.000 | 0.000 | 0.000 |

| **Primary ID** | **RT19** | **RT20** | **RT21** | **RT22** | **RT23** | **RT24** | **RT25** | **RT26** | **RT27** | **RT28** | **RT29** | **RT30** | **RT31** | **RT32** | **RT33** | **RT34** | **RT35** | **RT36** |
| --- | --- | --- | --- | --- | --- | --- | --- | --- | --- | --- | --- | --- | --- | --- | --- | --- | --- | --- |
| S1 | 24.431 | 0.000 | 2073.640 | 0.000 | 0.000 | 0.000 | 12.407 | 0.000 | 64.689 | 0.000 | 0.000 | 0.000 | 0.000 | 0.000 | 81.929 | 0.000 | 0.000 | 0.000 |
| S2 | 30.176 | 0.000 | 2225.270 | 0.000 | 0.000 | 0.000 | 10.500 | 0.000 | 87.562 | 0.000 | 0.000 | 0.000 | 0.000 | 0.000 | 34.029 | 0.000 | 0.000 | 0.000 |
| S3 | 17.095 | 0.000 | 1996.920 | 0.000 | 0.000 | 0.000 | 13.278 | 0.000 | 63.533 | 0.000 | 0.000 | 0.000 | 0.000 | 0.000 | 34.911 | 0.000 | 0.000 | 0.000 |
| S4 | 39.570 | 0.000 | 1767.280 | 0.000 | 0.000 | 0.000 | 12.198 | 0.000 | 148.204 | 0.000 | 0.000 | 0.000 | 0.000 | 0.000 | 48.693 | 0.000 | 0.000 | 0.000 |
| S5 | 23.486 | 0.000 | 1279.870 | 0.000 | 0.000 | 0.000 | 0.000 | 0.000 | 112.938 | 0.000 | 0.000 | 0.000 | 0.000 | 0.000 | 32.184 | 0.000 | 0.000 | 0.000 |
| S6 | 25.289 | 0.000 | 1753.810 | 0.000 | 0.000 | 0.000 | 13.799 | 0.000 | 83.573 | 0.000 | 0.000 | 0.000 | 0.000 | 0.000 | 44.209 | 0.000 | 0.000 | 0.000 |
| S7 | 43.392 | 0.000 | 2512.590 | 0.000 | 0.000 | 0.000 | 13.470 | 0.000 | 130.775 | 0.000 | 0.000 | 0.000 | 0.000 | 0.000 | 124.095 | 0.000 | 0.000 | 0.000 |
| S8 | 83.950 | 0.000 | 2127.790 | 0.000 | 0.000 | 0.000 | 0.000 | 0.000 | 243.871 | 0.000 | 0.000 | 0.000 | 0.000 | 0.000 | 44.100 | 0.000 | 0.000 | 0.000 |
| S9 | 74.086 | 0.000 | 1537.510 | 0.000 | 0.000 | 0.000 | 12.737 | 0.000 | 211.266 | 0.000 | 0.000 | 0.000 | 0.000 | 0.000 | 47.547 | 0.000 | 0.000 | 0.000 |
| S10 | 131.885 | 0.000 | 1367.950 | 0.000 | 0.000 | 0.000 | 0.000 | 0.000 | 363.859 | 0.000 | 0.000 | 0.000 | 0.000 | 0.000 | 65.993 | 0.000 | 0.000 | 0.000 |
| S11 | 66.873 | 0.000 | 1529.920 | 0.000 | 0.000 | 0.000 | 0.000 | 0.000 | 227.280 | 0.000 | 0.000 | 0.000 | 0.000 | 0.000 | 88.076 | 0.000 | 0.000 | 0.000 |
| S12 | 58.744 | 0.000 | 1411.310 | 0.000 | 0.000 | 0.000 | 11.352 | 0.000 | 133.922 | 0.000 | 0.000 | 0.000 | 0.000 | 0.000 | 52.564 | 0.000 | 0.000 | 0.000 |
| S13 | 26.893 | 0.000 | 1663.690 | 0.000 | 0.000 | 0.000 | 11.351 | 0.000 | 87.722 | 0.000 | 0.000 | 0.000 | 0.000 | 0.000 | 117.930 | 0.000 | 0.000 | 0.000 |
| S14 | 26.371 | 0.000 | 1562.970 | 0.000 | 0.000 | 0.000 | 0.000 | 0.000 | 116.025 | 0.000 | 0.000 | 0.000 | 0.000 | 0.000 | 50.046 | 0.000 | 0.000 | 0.000 |
| S15 | 88.699 | 0.000 | 1457.080 | 0.000 | 0.000 | 0.000 | 12.454 | 0.000 | 415.012 | 0.000 | 14.598 | 18.193 | 0.000 | 0.000 | 51.265 | 0.000 | 0.000 | 0.000 |
| S16 | 136.153 | 0.000 | 1251.150 | 0.000 | 0.000 | 0.000 | 11.182 | 0.000 | 451.235 | 0.000 | 13.868 | 0.000 | 0.000 | 0.000 | 45.387 | 0.000 | 0.000 | 0.000 |
| S17 | 60.932 | 0.000 | 1412.600 | 0.000 | 0.000 | 0.000 | 12.390 | 0.000 | 199.957 | 0.000 | 20.125 | 0.000 | 0.000 | 0.000 | 56.582 | 0.000 | 0.000 | 0.000 |
| S18 | 130.438 | 0.000 | 983.372 | 0.000 | 0.000 | 0.000 | 0.000 | 0.000 | 352.364 | 0.000 | 0.000 | 0.000 | 0.000 | 0.000 | 38.139 | 0.000 | 0.000 | 0.000 |
| S19 | 50.798 | 0.000 | 459.652 | 118.713 | 0.000 | 0.000 | 0.000 | 0.000 | 84.581 | 0.000 | 126.218 | 0.000 | 0.000 | 0.000 | 20.276 | 0.000 | 0.000 | 0.000 |
| S20 | 52.935 | 0.000 | 407.257 | 79.447 | 0.000 | 0.000 | 11.437 | 0.000 | 154.456 | 0.000 | 182.407 | 0.000 | 0.000 | 0.000 | 10.307 | 0.000 | 0.000 | 0.000 |
| S21 | 53.551 | 0.000 | 716.569 | 25.394 | 0.000 | 0.000 | 10.895 | 14.513 | 409.067 | 0.000 | 184.440 | 0.000 | 0.000 | 25.132 | 19.859 | 0.000 | 0.000 | 18.188 |
| S22 | 83.513 | 0.000 | 654.641 | 0.000 | 0.000 | 0.000 | 16.203 | 10.393 | 490.463 | 0.000 | 181.808 | 0.000 | 0.000 | 15.314 | 15.493 | 0.000 | 0.000 | 0.000 |
| S23 | 41.776 | 0.000 | 469.104 | 54.351 | 0.000 | 0.000 | 10.005 | 0.000 | 80.113 | 0.000 | 101.849 | 0.000 | 0.000 | 0.000 | 10.886 | 0.000 | 0.000 | 0.000 |
| S24 | 80.344 | 0.000 | 355.974 | 40.569 | 0.000 | 0.000 | 0.000 | 0.000 | 168.234 | 0.000 | 80.810 | 0.000 | 0.000 | 0.000 | 12.360 | 0.000 | 0.000 | 0.000 |
| S25 | 16.586 | 0.000 | 166.023 | 27.863 | 0.000 | 0.000 | 14.660 | 21.457 | 108.838 | 0.000 | 239.882 | 0.000 | 0.000 | 0.000 | 0.000 | 11.900 | 0.000 | 0.000 |
| S26 | 48.045 | 0.000 | 62.726 | 18.553 | 0.000 | 0.000 | 0.000 | 0.000 | 53.504 | 0.000 | 76.157 | 0.000 | 0.000 | 0.000 | 0.000 | 0.000 | 13.324 | 0.000 |
| S27 | 56.613 | 0.000 | 110.069 | 16.345 | 0.000 | 0.000 | 0.000 | 0.000 | 72.765 | 0.000 | 103.231 | 0.000 | 0.000 | 0.000 | 0.000 | 0.000 | 13.643 | 0.000 |
| S28 | 0.000 | 0.000 | 202.013 | 43.093 | 12.084 | 10.926 | 0.000 | 20.239 | 781.264 | 0.000 | 132.299 | 0.000 | 38.369 | 73.337 | 0.000 | 0.000 | 0.000 | 67.155 |
| S29 | 16.027 | 0.000 | 205.088 | 53.867 | 11.342 | 0.000 | 10.765 | 27.891 | 804.978 | 0.000 | 273.851 | 43.555 | 30.646 | 102.862 | 0.000 | 0.000 | 0.000 | 97.053 |
| S30 | 14.800 | 0.000 | 329.751 | 41.681 | 15.654 | 0.000 | 16.017 | 40.668 | 749.906 | 0.000 | 333.775 | 0.000 | 0.000 | 94.074 | 0.000 | 0.000 | 0.000 | 0.000 |
| S31 | 17.044 | 0.000 | 58.700 | 16.052 | 0.000 | 0.000 | 0.000 | 0.000 | 78.090 | 0.000 | 169.940 | 0.000 | 0.000 | 0.000 | 0.000 | 0.000 | 0.000 | 29.192 |
| S32 | 23.653 | 0.000 | 55.667 | 0.000 | 0.000 | 0.000 | 0.000 | 0.000 | 107.166 | 0.000 | 32.008 | 0.000 | 0.000 | 0.000 | 0.000 | 0.000 | 0.000 | 0.000 |
| S33 | 92.632 | 0.000 | 77.769 | 20.690 | 0.000 | 0.000 | 0.000 | 0.000 | 65.038 | 0.000 | 96.062 | 0.000 | 0.000 | 0.000 | 0.000 | 0.000 | 12.517 | 0.000 |
| S34 | 71.330 | 0.000 | 87.658 | 42.129 | 0.000 | 0.000 | 0.000 | 0.000 | 145.523 | 0.000 | 105.667 | 0.000 | 0.000 | 0.000 | 0.000 | 0.000 | 0.000 | 0.000 |
| S35 | 48.041 | 0.000 | 112.467 | 0.000 | 0.000 | 0.000 | 0.000 | 0.000 | 80.124 | 0.000 | 152.577 | 0.000 | 0.000 | 0.000 | 0.000 | 0.000 | 0.000 | 0.000 |
| S36 | 50.135 | 0.000 | 41.768 | 15.253 | 0.000 | 0.000 | 0.000 | 0.000 | 46.619 | 0.000 | 77.030 | 0.000 | 0.000 | 0.000 | 0.000 | 0.000 | 17.229 | 0.000 |
| S37 | 44.805 | 0.000 | 75.823 | 17.894 | 0.000 | 0.000 | 0.000 | 0.000 | 71.033 | 0.000 | 159.204 | 0.000 | 0.000 | 0.000 | 0.000 | 0.000 | 10.479 | 0.000 |
| S38 | 32.087 | 0.000 | 78.264 | 19.852 | 0.000 | 0.000 | 0.000 | 17.207 | 265.841 | 0.000 | 137.593 | 0.000 | 0.000 | 19.024 | 0.000 | 0.000 | 0.000 | 0.000 |
| S39 | 64.641 | 0.000 | 285.251 | 0.000 | 0.000 | 0.000 | 0.000 | 0.000 | 153.305 | 0.000 | 225.545 | 0.000 | 0.000 | 0.000 | 0.000 | 0.000 | 10.017 | 0.000 |
| S40 | 32.228 | 0.000 | 38.868 | 0.000 | 0.000 | 0.000 | 0.000 | 0.000 | 37.762 | 0.000 | 0.000 | 0.000 | 0.000 | 0.000 | 0.000 | 18.648 | 0.000 | 0.000 |
| S41 | 48.852 | 0.000 | 94.872 | 32.788 | 0.000 | 0.000 | 0.000 | 0.000 | 58.579 | 0.000 | 84.659 | 0.000 | 0.000 | 0.000 | 0.000 | 0.000 | 10.813 | 0.000 |
| S42 | 44.626 | 0.000 | 212.284 | 25.153 | 0.000 | 0.000 | 23.884 | 14.202 | 445.350 | 0.000 | 263.852 | 0.000 | 0.000 | 31.118 | 0.000 | 0.000 | 0.000 | 0.000 |
| S43 | 44.268 | 11.837 | 182.377 | 76.455 | 0.000 | 0.000 | 15.584 | 25.051 | 208.025 | 0.000 | 0.000 | 0.000 | 0.000 | 0.000 | 0.000 | 0.000 | 0.000 | 0.000 |
| S44 | 0.000 | 0.000 | 62.388 | 129.291 | 0.000 | 0.000 | 15.558 | 16.482 | 90.351 | 0.000 | 318.209 | 0.000 | 0.000 | 0.000 | 0.000 | 0.000 | 0.000 | 0.000 |
| S45 | 22.046 | 0.000 | 195.894 | 144.571 | 0.000 | 0.000 | 0.000 | 0.000 | 152.039 | 0.000 | 176.523 | 0.000 | 0.000 | 19.381 | 0.000 | 0.000 | 0.000 | 42.951 |
| S46 | 20.179 | 0.000 | 43.933 | 0.000 | 0.000 | 0.000 | 0.000 | 0.000 | 188.522 | 0.000 | 17.492 | 0.000 | 0.000 | 0.000 | 0.000 | 0.000 | 0.000 | 0.000 |
| S47 | 18.026 | 0.000 | 45.411 | 10.528 | 0.000 | 0.000 | 0.000 | 0.000 | 90.211 | 0.000 | 14.089 | 0.000 | 0.000 | 0.000 | 0.000 | 0.000 | 11.304 | 0.000 |
| S48 | 28.847 | 0.000 | 46.963 | 0.000 | 0.000 | 0.000 | 0.000 | 0.000 | 129.332 | 0.000 | 0.000 | 0.000 | 0.000 | 0.000 | 0.000 | 0.000 | 0.000 | 0.000 |
| S49 | 34.057 | 0.000 | 154.570 | 0.000 | 0.000 | 0.000 | 0.000 | 0.000 | 188.297 | 15.548 | 66.850 | 0.000 | 0.000 | 0.000 | 0.000 | 0.000 | 0.000 | 0.000 |
| S50 | 48.739 | 0.000 | 310.868 | 0.000 | 0.000 | 0.000 | 0.000 | 20.912 | 478.694 | 17.288 | 182.914 | 0.000 | 0.000 | 23.538 | 0.000 | 0.000 | 12.457 | 0.000 |
| S51 | 46.641 | 0.000 | 332.820 | 0.000 | 11.283 | 0.000 | 0.000 | 20.002 | 169.670 | 0.000 | 146.240 | 0.000 | 0.000 | 0.000 | 0.000 | 0.000 | 13.295 | 0.000 |

| **Primary ID** | **RT37** | **RT38** | **RT39** | **RT40** | **RT41** | **RT42** | **RT43** | **RT44** | **RT45** | **RT46** | **RT47** | **RT48** | **RT49** | **RT50** | **RT51** | **RT52** | **RT53** | **RT54** |
| --- | --- | --- | --- | --- | --- | --- | --- | --- | --- | --- | --- | --- | --- | --- | --- | --- | --- | --- |
| S1 | 0.000 | 0.000 | 0.000 | 113.442 | 0.000 | 0.000 | 16.462 | 0.000 | 0.000 | 0.000 | 0.000 | 0.000 | 0.000 | 0.000 | 0.000 | 0.000 | 0.000 | 0.000 |
| S2 | 0.000 | 0.000 | 0.000 | 134.059 | 0.000 | 0.000 | 29.126 | 0.000 | 0.000 | 13.664 | 0.000 | 0.000 | 0.000 | 0.000 | 0.000 | 0.000 | 0.000 | 0.000 |
| S3 | 0.000 | 0.000 | 0.000 | 136.521 | 0.000 | 0.000 | 21.003 | 0.000 | 0.000 | 16.117 | 0.000 | 0.000 | 0.000 | 0.000 | 0.000 | 0.000 | 0.000 | 0.000 |
| S4 | 0.000 | 0.000 | 0.000 | 91.323 | 0.000 | 0.000 | 15.398 | 0.000 | 0.000 | 0.000 | 0.000 | 0.000 | 0.000 | 0.000 | 0.000 | 0.000 | 0.000 | 0.000 |
| S5 | 0.000 | 0.000 | 0.000 | 72.266 | 0.000 | 0.000 | 0.000 | 0.000 | 0.000 | 0.000 | 0.000 | 0.000 | 0.000 | 0.000 | 0.000 | 0.000 | 0.000 | 0.000 |
| S6 | 0.000 | 0.000 | 0.000 | 108.970 | 0.000 | 0.000 | 14.589 | 0.000 | 0.000 | 0.000 | 0.000 | 0.000 | 0.000 | 0.000 | 0.000 | 0.000 | 0.000 | 0.000 |
| S7 | 0.000 | 0.000 | 0.000 | 113.204 | 0.000 | 0.000 | 30.468 | 0.000 | 0.000 | 0.000 | 0.000 | 0.000 | 0.000 | 0.000 | 0.000 | 0.000 | 0.000 | 0.000 |
| S8 | 0.000 | 0.000 | 0.000 | 125.111 | 0.000 | 0.000 | 97.336 | 0.000 | 0.000 | 32.055 | 0.000 | 0.000 | 0.000 | 0.000 | 0.000 | 0.000 | 0.000 | 0.000 |
| S9 | 0.000 | 0.000 | 0.000 | 115.354 | 0.000 | 0.000 | 131.327 | 0.000 | 0.000 | 62.478 | 0.000 | 0.000 | 0.000 | 0.000 | 0.000 | 0.000 | 0.000 | 0.000 |
| S10 | 0.000 | 0.000 | 0.000 | 84.238 | 0.000 | 0.000 | 80.867 | 0.000 | 0.000 | 28.376 | 0.000 | 0.000 | 0.000 | 0.000 | 0.000 | 0.000 | 0.000 | 0.000 |
| S11 | 0.000 | 0.000 | 0.000 | 79.364 | 0.000 | 0.000 | 78.060 | 0.000 | 0.000 | 37.739 | 0.000 | 0.000 | 0.000 | 0.000 | 0.000 | 0.000 | 0.000 | 0.000 |
| S12 | 0.000 | 0.000 | 0.000 | 108.773 | 0.000 | 0.000 | 88.778 | 0.000 | 0.000 | 32.849 | 0.000 | 0.000 | 0.000 | 0.000 | 0.000 | 0.000 | 0.000 | 0.000 |
| S13 | 0.000 | 0.000 | 0.000 | 62.448 | 0.000 | 0.000 | 67.594 | 0.000 | 0.000 | 32.667 | 0.000 | 0.000 | 0.000 | 0.000 | 0.000 | 36.083 | 0.000 | 0.000 |
| S14 | 0.000 | 0.000 | 0.000 | 45.213 | 0.000 | 0.000 | 273.903 | 0.000 | 0.000 | 147.312 | 0.000 | 0.000 | 12.012 | 11.709 | 0.000 | 84.629 | 0.000 | 0.000 |
| S15 | 0.000 | 0.000 | 0.000 | 80.424 | 0.000 | 0.000 | 395.159 | 0.000 | 0.000 | 245.869 | 0.000 | 0.000 | 0.000 | 24.496 | 0.000 | 55.287 | 0.000 | 0.000 |
| S16 | 0.000 | 0.000 | 0.000 | 80.948 | 0.000 | 0.000 | 377.451 | 0.000 | 0.000 | 219.555 | 0.000 | 0.000 | 0.000 | 25.308 | 0.000 | 50.871 | 0.000 | 0.000 |
| S17 | 0.000 | 0.000 | 0.000 | 64.042 | 0.000 | 0.000 | 574.616 | 0.000 | 0.000 | 291.650 | 0.000 | 0.000 | 18.939 | 53.587 | 0.000 | 160.922 | 0.000 | 0.000 |
| S18 | 0.000 | 0.000 | 0.000 | 69.150 | 0.000 | 0.000 | 153.114 | 0.000 | 0.000 | 97.047 | 0.000 | 0.000 | 0.000 | 0.000 | 0.000 | 0.000 | 0.000 | 0.000 |
| S19 | 0.000 | 0.000 | 32.622 | 17.024 | 0.000 | 0.000 | 1329.780 | 0.000 | 0.000 | 277.658 | 0.000 | 0.000 | 136.279 | 321.963 | 0.000 | 1134.070 | 0.000 | 53.769 |
| S20 | 0.000 | 0.000 | 23.282 | 14.846 | 0.000 | 0.000 | 1483.920 | 110.535 | 0.000 | 281.055 | 0.000 | 0.000 | 265.005 | 222.521 | 0.000 | 959.658 | 0.000 | 17.419 |
| S21 | 0.000 | 0.000 | 34.931 | 29.521 | 0.000 | 98.408 | 1230.080 | 117.804 | 0.000 | 325.904 | 0.000 | 0.000 | 82.850 | 200.916 | 0.000 | 523.816 | 0.000 | 16.153 |
| S22 | 0.000 | 0.000 | 22.889 | 23.984 | 0.000 | 95.576 | 1251.030 | 177.841 | 0.000 | 194.902 | 0.000 | 0.000 | 0.000 | 143.654 | 23.133 | 19.616 | 0.000 | 14.374 |
| S23 | 0.000 | 0.000 | 51.532 | 0.000 | 0.000 | 10.301 | 1362.540 | 70.702 | 0.000 | 308.259 | 0.000 | 0.000 | 103.767 | 213.587 | 0.000 | 648.456 | 0.000 | 68.780 |
| S24 | 0.000 | 0.000 | 14.702 | 14.368 | 0.000 | 0.000 | 764.846 | 42.474 | 0.000 | 78.185 | 0.000 | 0.000 | 0.000 | 94.612 | 0.000 | 0.000 | 0.000 | 0.000 |
| S25 | 0.000 | 12.469 | 26.821 | 0.000 | 0.000 | 94.004 | 964.829 | 148.199 | 0.000 | 149.010 | 0.000 | 0.000 | 0.000 | 164.098 | 31.761 | 24.455 | 0.000 | 136.120 |
| S26 | 0.000 | 0.000 | 0.000 | 0.000 | 0.000 | 54.609 | 304.213 | 123.384 | 0.000 | 68.432 | 0.000 | 0.000 | 0.000 | 63.398 | 0.000 | 48.115 | 0.000 | 0.000 |
| S27 | 0.000 | 0.000 | 0.000 | 0.000 | 0.000 | 45.335 | 583.907 | 134.548 | 0.000 | 121.557 | 0.000 | 0.000 | 0.000 | 91.786 | 0.000 | 0.000 | 0.000 | 25.459 |
| S28 | 0.000 | 0.000 | 36.278 | 0.000 | 0.000 | 82.988 | 1808.620 | 100.361 | 22.730 | 271.400 | 45.660 | 237.313 | 0.000 | 252.580 | 0.000 | 829.082 | 0.000 | 47.381 |
| S29 | 0.000 | 0.000 | 53.144 | 0.000 | 0.000 | 207.474 | 1358.590 | 101.938 | 21.864 | 284.905 | 50.902 | 184.617 | 0.000 | 249.428 | 25.662 | 831.563 | 0.000 | 60.378 |
| S30 | 12.351 | 0.000 | 34.203 | 0.000 | 0.000 | 228.498 | 1157.490 | 143.652 | 0.000 | 193.873 | 34.390 | 50.590 | 0.000 | 210.446 | 12.018 | 159.553 | 0.000 | 24.539 |
| S31 | 0.000 | 0.000 | 42.581 | 0.000 | 0.000 | 31.354 | 497.688 | 46.456 | 0.000 | 294.802 | 0.000 | 322.928 | 0.000 | 219.390 | 26.115 | 867.792 | 0.000 | 481.303 |
| S32 | 0.000 | 0.000 | 32.014 | 0.000 | 0.000 | 95.068 | 930.309 | 190.690 | 0.000 | 143.910 | 0.000 | 0.000 | 0.000 | 146.485 | 0.000 | 0.000 | 0.000 | 26.840 |
| S33 | 0.000 | 0.000 | 12.368 | 0.000 | 0.000 | 0.000 | 557.873 | 62.495 | 0.000 | 65.626 | 0.000 | 0.000 | 0.000 | 77.550 | 0.000 | 0.000 | 0.000 | 10.442 |
| S34 | 0.000 | 0.000 | 33.638 | 0.000 | 0.000 | 16.272 | 1362.080 | 72.367 | 0.000 | 190.763 | 0.000 | 134.167 | 0.000 | 243.167 | 0.000 | 813.138 | 0.000 | 163.828 |
| S35 | 0.000 | 0.000 | 46.657 | 0.000 | 0.000 | 64.813 | 983.965 | 108.610 | 0.000 | 224.772 | 0.000 | 21.151 | 0.000 | 184.856 | 31.308 | 219.126 | 0.000 | 133.901 |
| S36 | 0.000 | 0.000 | 30.745 | 0.000 | 0.000 | 16.694 | 465.084 | 47.240 |  | 150.728 | 0.000 | 59.672 | 0.000 | 145.175 | 0.000 | 388.790 | 0.000 | 40.851 |
| S37 | 0.000 | 0.000 | 32.768 | 0.000 | 0.000 | 76.044 | 958.882 | 128.331 | 0.000 | 228.604 | 0.000 | 60.069 | 0.000 | 195.818 | 26.405 | 447.954 | 0.000 | 27.852 |
| S38 | 11.753 | 0.000 | 38.754 | 0.000 | 12.189 | 104.626 | 1078.800 | 131.928 | 0.000 | 206.228 | 0.000 | 45.270 | 0.000 | 169.839 | 16.745 | 266.714 | 0.000 | 16.801 |
| S39 | 15.955 | 0.000 | 45.981 | 0.000 | 14.948 | 120.638 | 1100.210 | 211.171 | 0.000 | 282.356 | 0.000 | 0.000 | 0.000 | 180.488 | 23.999 | 0.000 | 0.000 | 330.941 |
| S40 | 0.000 | 0.000 | 0.000 | 0.000 | 0.000 | 0.000 | 67.662 | 0.000 | 0.000 | 24.651 | 0.000 | 0.000 | 0.000 | 21.677 | 0.000 | 72.057 | 0.000 | 48.903 |
| S41 | 0.000 | 0.000 | 22.791 | 0.000 | 0.000 | 25.122 | 533.187 | 71.774 | 0.000 | 173.789 | 0.000 | 80.704 | 0.000 | 155.851 | 0.000 | 517.168 | 0.000 | 27.098 |
| S42 | 18.513 | 0.000 | 32.210 | 0.000 | 0.000 | 178.425 | 1459.410 | 220.105 | 0.000 | 198.893 | 0.000 | 0.000 | 0.000 | 185.694 | 23.396 | 0.000 | 0.000 | 27.910 |
| S43 | 0.000 | 0.000 | 13.131 | 0.000 | 0.000 | 13.334 | 2177.110 | 0.000 | 0.000 | 601.136 | 0.000 | 0.000 | 0.000 | 217.635 | 0.000 | 0.000 | 0.000 | 12.101 |
| S44 | 0.000 | 0.000 | 29.473 | 0.000 | 0.000 | 0.000 | 2270.310 | 151.345 | 0.000 | 334.154 | 0.000 | 400.045 | 0.000 | 315.118 | 16.285 | 1366.260 | 0.000 | 49.196 |
| S45 | 13.333 | 0.000 | 46.182 | 0.000 | 0.000 | 49.958 | 1612.560 | 0.000 | 0.000 | 318.486 | 41.034 | 195.326 | 0.000 | 424.701 | 18.451 | 1378.610 | 0.000 | 27.670 |
| S46 | 0.000 | 0.000 | 0.000 | 0.000 | 0.000 | 0.000 | 490.806 | 30.330 | 0.000 | 134.599 | 0.000 | 11.614 | 0.000 | 51.905 | 0.000 | 123.352 | 0.000 | 174.098 |
| S47 | 0.000 | 0.000 | 0.000 | 0.000 | 0.000 | 0.000 | 250.896 | 21.477 | 0.000 | 84.321 | 0.000 | 10.928 | 0.000 | 34.192 | 0.000 | 126.664 | 0.000 | 99.599 |
| S48 | 0.000 | 0.000 | 0.000 | 0.000 | 0.000 | 0.000 | 186.447 | 21.798 | 0.000 | 37.862 | 0.000 | 0.000 | 0.000 | 15.886 | 0.000 | 0.000 | 0.000 | 31.566 |
| S49 | 13.262 | 0.000 | 19.733 | 0.000 | 31.857 | 35.566 | 739.456 | 31.568 | 0.000 | 304.610 | 0.000 | 51.078 | 0.000 | 132.649 | 0.000 | 404.397 | 0.000 | 3944.680 |
| S50 | 0.000 | 0.000 | 16.634 | 0.000 | 28.072 | 110.830 | 839.428 | 159.256 | 0.000 | 330.236 | 0.000 | 0.000 | 0.000 | 111.409 | 19.955 | 0.000 | 19.223 | 5219.320 |
| S51 | 0.000 | 0.000 | 0.000 | 0.000 | 17.729 | 48.916 | 632.084 | 120.612 | 0.000 | 236.661 | 0.000 | 0.000 | 0.000 | 82.457 | 0.000 | 0.000 | 0.000 | 3234.060 |

| **Primary ID** | **RT55** | **RT56** | **RT57** | **RT58** | **RT59** | **RT60** | **RT61** | **RT62** | **RT63** | **RT64** | **RT65** | **RT66** | **RT67** | **RT68** | **RT69** | **RT70** | **RT71** | **RT72** |
| --- | --- | --- | --- | --- | --- | --- | --- | --- | --- | --- | --- | --- | --- | --- | --- | --- | --- | --- |
| S1 | 0.000 | 0.000 | 0.000 | 0.000 | 0.000 | 0.000 | 0.000 | 0.000 | 0.000 | 0.000 | 0.000 | 0.000 | 0.000 | 0.000 | 0.000 | 0.000 | 0.000 | 0.000 |
| S2 | 0.000 | 0.000 | 0.000 | 0.000 | 0.000 | 0.000 | 0.000 | 0.000 | 0.000 | 0.000 | 0.000 | 0.000 | 0.000 | 0.000 | 0.000 | 0.000 | 0.000 | 0.000 |
| S3 | 0.000 | 0.000 | 0.000 | 0.000 | 0.000 | 0.000 | 0.000 | 0.000 | 0.000 | 0.000 | 0.000 | 0.000 | 0.000 | 0.000 | 0.000 | 0.000 | 0.000 | 0.000 |
| S4 | 0.000 | 0.000 | 0.000 | 0.000 | 0.000 | 0.000 | 0.000 | 0.000 | 0.000 | 0.000 | 0.000 | 0.000 | 0.000 | 0.000 | 0.000 | 0.000 | 0.000 | 0.000 |
| S5 | 0.000 | 0.000 | 0.000 | 0.000 | 0.000 | 0.000 | 0.000 | 0.000 | 0.000 | 0.000 | 0.000 | 0.000 | 0.000 | 0.000 | 0.000 | 0.000 | 0.000 | 0.000 |
| S6 | 0.000 | 0.000 | 0.000 | 0.000 | 0.000 | 0.000 | 0.000 | 0.000 | 0.000 | 0.000 | 0.000 | 0.000 | 0.000 | 0.000 | 0.000 | 0.000 | 0.000 | 0.000 |
| S7 | 0.000 | 0.000 | 0.000 | 0.000 | 0.000 | 0.000 | 0.000 | 0.000 | 0.000 | 0.000 | 0.000 | 0.000 | 0.000 | 0.000 | 0.000 | 0.000 | 0.000 | 0.000 |
| S8 | 0.000 | 0.000 | 0.000 | 0.000 | 0.000 | 0.000 | 0.000 | 0.000 | 0.000 | 0.000 | 0.000 | 0.000 | 0.000 | 0.000 | 0.000 | 0.000 | 0.000 | 0.000 |
| S9 | 0.000 | 0.000 | 0.000 | 10.972 | 0.000 | 0.000 | 0.000 | 0.000 | 0.000 | 0.000 | 0.000 | 0.000 | 0.000 | 0.000 | 0.000 | 0.000 | 0.000 | 0.000 |
| S10 | 0.000 | 0.000 | 0.000 | 0.000 | 0.000 | 0.000 | 0.000 | 0.000 | 0.000 | 0.000 | 0.000 | 0.000 | 0.000 | 0.000 | 0.000 | 0.000 | 0.000 | 0.000 |
| S11 | 0.000 | 0.000 | 0.000 | 0.000 | 0.000 | 0.000 | 0.000 | 0.000 | 0.000 | 0.000 | 0.000 | 0.000 | 0.000 | 0.000 | 0.000 | 0.000 | 0.000 | 0.000 |
| S12 | 0.000 | 0.000 | 0.000 | 0.000 | 0.000 | 0.000 | 0.000 | 0.000 | 0.000 | 0.000 | 0.000 | 0.000 | 0.000 | 0.000 | 0.000 | 0.000 | 0.000 | 0.000 |
| S13 | 0.000 | 0.000 | 0.000 | 0.000 | 0.000 | 0.000 | 0.000 | 0.000 | 0.000 | 0.000 | 0.000 | 0.000 | 0.000 | 0.000 | 0.000 | 0.000 | 0.000 | 0.000 |
| S14 | 0.000 | 0.000 | 0.000 | 0.000 | 0.000 | 0.000 | 0.000 | 0.000 | 26.971 | 0.000 | 0.000 | 0.000 | 0.000 | 0.000 | 0.000 | 0.000 | 0.000 | 0.000 |
| S15 | 0.000 | 19.268 | 0.000 | 0.000 | 0.000 | 0.000 | 0.000 | 0.000 | 22.819 | 0.000 | 0.000 | 0.000 | 0.000 | 0.000 | 0.000 | 0.000 | 0.000 | 0.000 |
| S16 | 0.000 | 19.956 | 0.000 | 0.000 | 0.000 | 0.000 | 0.000 | 0.000 | 27.964 | 0.000 | 0.000 | 0.000 | 0.000 | 0.000 | 0.000 | 0.000 | 0.000 | 0.000 |
| S17 | 0.000 | 33.717 | 0.000 | 0.000 | 0.000 | 0.000 | 0.000 | 0.000 | 57.107 | 0.000 | 0.000 | 0.000 | 0.000 | 70.673 | 0.000 | 0.000 | 0.000 | 0.000 |
| S18 | 0.000 | 13.522 | 0.000 | 0.000 | 0.000 | 0.000 | 0.000 | 0.000 | 0.000 | 0.000 | 0.000 | 0.000 | 0.000 | 0.000 | 0.000 | 0.000 | 0.000 | 0.000 |
| S19 | 0.000 | 0.000 | 28.370 | 0.000 | 0.000 | 38.936 | 93.222 | 0.000 | 467.589 | 0.000 | 0.000 | 0.000 | 56.341 | 514.750 | 20.284 | 34.193 | 0.000 | 0.000 |
| S20 | 0.000 | 43.807 | 0.000 | 0.000 | 23.667 | 86.079 | 31.010 | 0.000 | 209.922 | 0.000 | 0.000 | 0.000 | 12.618 | 0.000 | 0.000 | 0.000 | 27.233 | 0.000 |
| S21 | 20.904 | 105.852 | 0.000 | 0.000 | 10.101 | 56.729 | 49.480 | 0.000 | 560.505 | 0.000 | 0.000 | 0.000 | 20.789 | 165.382 | 12.181 | 0.000 | 63.000 | 23.862 |
| S22 | 18.738 | 36.382 | 0.000 | 0.000 | 0.000 | 20.677 | 55.140 | 0.000 | 322.686 | 0.000 | 0.000 | 0.000 | 25.972 | 0.000 | 0.000 | 0.000 | 76.723 | 34.517 |
| S23 | 23.283 | 69.754 | 0.000 | 0.000 | 0.000 | 0.000 | 35.438 | 0.000 | 474.872 | 0.000 | 0.000 | 0.000 | 23.932 | 193.850 | 14.594 | 0.000 | 56.874 | 36.100 |
| S24 | 0.000 | 0.000 | 0.000 | 0.000 | 0.000 | 0.000 | 17.595 | 0.000 | 247.286 | 0.000 | 0.000 | 0.000 | 0.000 | 0.000 | 0.000 | 0.000 | 16.296 | 0.000 |
| S25 | 31.550 | 335.433 | 0.000 | 0.000 | 0.000 | 16.500 | 72.090 | 0.000 | 609.996 | 36.076 | 72.839 | 0.000 | 51.953 | 363.782 | 52.324 | 0.000 | 124.584 | 50.506 |
| S26 | 0.000 | 91.930 | 0.000 | 0.000 | 0.000 | 0.000 | 33.378 | 0.000 | 403.137 | 0.000 | 0.000 | 0.000 | 0.000 | 59.804 | 0.000 | 0.000 | 18.734 | 0.000 |
| S27 | 0.000 | 51.807 | 0.000 | 0.000 | 0.000 | 0.000 | 52.682 | 0.000 | 369.075 | 0.000 | 0.000 | 0.000 | 0.000 | 36.224 | 0.000 | 0.000 | 56.501 | 28.083 |
| S28 | 44.219 | 91.722 | 0.000 | 0.000 | 0.000 | 143.449 | 51.091 | 23.743 | 451.528 | 0.000 | 0.000 | 0.000 | 82.445 | 326.146 | 10.281 | 0.000 | 159.226 | 52.230 |
| S29 | 29.449 | 129.919 | 18.411 | 0.000 | 0.000 | 152.590 | 48.458 | 36.979 | 573.409 | 0.000 | 0.000 | 0.000 | 38.824 | 252.010 | 0.000 | 29.717 | 83.401 | 38.427 |
| S30 | 50.757 | 24.001 | 11.420 | 0.000 | 0.000 | 201.697 | 51.564 | 27.033 | 651.010 | 0.000 | 0.000 | 15.358 | 53.118 | 161.558 | 31.087 | 0.000 | 111.548 | 42.279 |
| S31 | 0.000 | 129.369 | 0.000 | 0.000 | 14.253 | 0.000 | 24.023 | 0.000 | 466.206 | 14.422 | 30.245 | 0.000 | 63.937 | 203.255 | 28.934 | 33.161 | 0.000 | 0.000 |
| S32 | 0.000 | 113.825 | 0.000 | 0.000 | 13.193 | 27.171 | 95.457 | 0.000 | 592.614 | 0.000 | 0.000 | 0.000 | 18.837 | 0.000 | 0.000 | 0.000 | 67.217 | 0.000 |
| S33 | 0.000 | 38.999 | 0.000 | 0.000 | 0.000 | 0.000 | 35.739 | 0.000 | 354.415 | 0.000 | 0.000 | 0.000 | 0.000 | 67.998 | 0.000 | 0.000 | 41.214 | 23.847 |
| S34 | 13.941 | 45.827 | 0.000 | 0.000 | 0.000 | 0.000 | 36.400 | 0.000 | 494.292 | 0.000 | 0.000 | 0.000 | 64.324 | 327.797 | 18.723 | 18.595 | 73.867 | 37.367 |
| S35 | 0.000 | 86.947 | 0.000 | 0.000 | 0.000 | 11.844 | 68.361 | 0.000 | 473.892 | 0.000 | 0.000 | 0.000 | 42.086 | 52.003 | 0.000 | 0.000 | 102.242 | 24.001 |
| S36 | 0.000 | 137.323 | 0.000 | 0.000 | 0.000 | 0.000 | 42.941 | 0.000 | 420.577 | 16.024 | 0.000 | 0.000 | 17.012 | 233.800 | 0.000 | 0.000 | 73.111 | 0.000 |
| S37 | 0.000 | 71.063 | 0.000 | 0.000 | 11.342 | 0.000 | 61.765 | 0.000 | 576.241 | 0.000 | 0.000 | 0.000 | 23.770 | 154.103 | 0.000 | 0.000 | 98.844 | 0.000 |
| S38 | 15.890 | 45.193 | 0.000 | 0.000 | 14.708 | 37.488 | 46.533 | 0.000 | 491.767 | 0.000 | 0.000 | 0.000 | 20.842 | 68.478 | 0.000 | 0.000 | 59.642 | 0.000 |
| S39 | 42.160 | 172.045 | 0.000 | 0.000 | 11.745 | 12.552 | 60.081 | 0.000 | 581.018 | 0.000 | 0.000 | 0.000 | 31.632 | 0.000 | 0.000 | 0.000 | 99.698 | 40.362 |
| S40 | 0.000 | 142.715 | 0.000 | 0.000 | 0.000 | 0.000 | 0.000 | 0.000 | 292.543 | 22.680 | 0.000 | 0.000 | 0.000 | 106.919 | 0.000 | 0.000 | 0.000 | 0.000 |
| S41 | 0.000 | 155.121 | 0.000 | 0.000 | 0.000 | 0.000 | 41.106 | 0.000 | 544.946 | 0.000 | 0.000 | 0.000 | 0.000 | 339.162 | 0.000 | 92.343 | 0.000 | 18.277 |
| S42 | 38.319 | 39.975 | 31.177 | 0.000 | 0.000 | 63.376 | 57.671 | 0.000 | 536.832 | 0.000 | 0.000 | 0.000 | 47.808 | 25.727 | 0.000 | 0.000 | 103.631 | 50.780 |
| S43 | 23.504 | 18.812 | 0.000 | 0.000 | 0.000 | 41.962 | 123.266 | 0.000 | 589.609 | 0.000 | 0.000 | 0.000 | 39.601 | 0.000 | 0.000 | 0.000 | 73.305 | 45.452 |
| S44 | 0.000 | 19.871 | 0.000 | 0.000 | 15.645 | 109.795 | 62.633 | 0.000 | 236.186 | 0.000 | 0.000 | 0.000 | 39.447 | 0.000 | 0.000 | 0.000 | 43.243 | 0.000 |
| S45 | 0.000 | 19.599 | 0.000 | 0.000 | 0.000 | 106.135 | 98.220 | 0.000 | 592.031 | 0.000 | 0.000 | 0.000 | 82.603 | 715.604 | 28.627 | 59.895 | 0.000 | 0.000 |
| S46 | 0.000 | 51.288 | 0.000 | 0.000 | 0.000 | 0.000 | 0.000 | 0.000 | 74.429 | 0.000 | 0.000 | 0.000 | 0.000 | 46.756 | 0.000 | 0.000 | 0.000 | 0.000 |
| S47 | 0.000 | 70.482 | 0.000 | 0.000 | 0.000 | 0.000 | 0.000 | 0.000 | 92.477 | 0.000 | 0.000 | 0.000 | 0.000 | 80.094 | 0.000 | 17.766 | 0.000 | 0.000 |
| S48 | 0.000 | 0.000 | 0.000 | 0.000 | 0.000 | 0.000 | 0.000 | 0.000 | 19.771 | 0.000 | 0.000 | 0.000 | 0.000 | 0.000 | 0.000 | 0.000 | 0.000 | 0.000 |
| S49 | 0.000 | 321.692 | 0.000 | 0.000 | 0.000 | 0.000 | 0.000 | 0.000 | 250.672 | 0.000 | 0.000 | 22.697 | 93.898 | 148.355 | 0.000 | 0.000 | 0.000 | 55.525 |
| S50 | 0.000 | 211.754 | 20.760 | 0.000 | 0.000 | 28.645 | 16.502 | 0.000 | 282.279 | 0.000 | 0.000 | 0.000 | 125.670 | 34.386 | 0.000 | 0.000 | 0.000 | 38.942 |
| S51 | 0.000 | 207.254 | 0.000 | 0.000 | 0.000 | 0.000 | 12.021 | 0.000 | 273.763 | 20.141 | 0.000 | 0.000 | 69.952 | 43.278 | 0.000 | 0.000 | 0.000 | 47.108 |

| **Primary ID** | **RT73** | **RT74** | **RT75** | **RT76** | **RT77** | **RT78** | **RT79** | **RT80** | **RT81** | **RT82** | **RT83** | **RT84** | **RT85** | **RT86** |
| --- | --- | --- | --- | --- | --- | --- | --- | --- | --- | --- | --- | --- | --- | --- |
| S1 | 0.000 | 0.000 | 0.000 | 0.000 | 0.000 | 0.000 | 0.000 | 0.000 | 0.000 | 0.000 | 0.000 | 0.000 | 0.000 | 0.000 |
| S2 | 0.000 | 0.000 | 0.000 | 0.000 | 0.000 | 0.000 | 0.000 | 0.000 | 0.000 | 0.000 | 0.000 | 0.000 | 0.000 | 0.000 |
| S3 | 0.000 | 0.000 | 0.000 | 0.000 | 0.000 | 0.000 | 0.000 | 0.000 | 0.000 | 0.000 | 0.000 | 0.000 | 0.000 | 0.000 |
| S4 | 0.000 | 0.000 | 0.000 | 0.000 | 0.000 | 0.000 | 0.000 | 0.000 | 0.000 | 0.000 | 0.000 | 0.000 | 0.000 | 0.000 |
| S5 | 0.000 | 0.000 | 0.000 | 0.000 | 0.000 | 0.000 | 0.000 | 0.000 | 0.000 | 0.000 | 0.000 | 0.000 | 0.000 | 0.000 |
| S6 | 0.000 | 0.000 | 0.000 | 0.000 | 0.000 | 0.000 | 0.000 | 0.000 | 0.000 | 0.000 | 0.000 | 0.000 | 0.000 | 0.000 |
| S7 | 0.000 | 0.000 | 0.000 | 0.000 | 0.000 | 0.000 | 0.000 | 0.000 | 0.000 | 0.000 | 0.000 | 0.000 | 0.000 | 0.000 |
| S8 | 0.000 | 0.000 | 0.000 | 0.000 | 0.000 | 0.000 | 0.000 | 0.000 | 0.000 | 0.000 | 0.000 | 0.000 | 0.000 | 0.000 |
| S9 | 0.000 | 0.000 | 0.000 | 0.000 | 0.000 | 0.000 | 0.000 | 0.000 | 0.000 | 0.000 | 0.000 | 0.000 | 0.000 | 0.000 |
| S10 | 0.000 | 0.000 | 0.000 | 0.000 | 0.000 | 0.000 | 0.000 | 0.000 | 0.000 | 0.000 | 0.000 | 0.000 | 0.000 | 0.000 |
| S11 | 0.000 | 0.000 | 0.000 | 0.000 | 0.000 | 0.000 | 0.000 | 0.000 | 0.000 | 0.000 | 0.000 | 0.000 | 0.000 | 0.000 |
| S12 | 0.000 | 0.000 | 0.000 | 0.000 | 0.000 | 0.000 | 0.000 | 0.000 | 0.000 | 0.000 | 0.000 | 0.000 | 0.000 | 0.000 |
| S13 | 0.000 | 0.000 | 0.000 | 0.000 | 0.000 | 0.000 | 0.000 | 0.000 | 0.000 | 0.000 | 0.000 | 0.000 | 0.000 | 0.000 |
| S14 | 0.000 | 0.000 | 0.000 | 0.000 | 0.000 | 0.000 | 0.000 | 0.000 | 0.000 | 0.000 | 0.000 | 0.000 | 0.000 | 0.000 |
| S15 | 0.000 | 0.000 | 0.000 | 0.000 | 18.477 | 0.000 | 0.000 | 0.000 | 0.000 | 0.000 | 0.000 | 0.000 | 0.000 | 0.000 |
| S16 | 0.000 | 0.000 | 0.000 | 0.000 | 10.345 | 0.000 | 0.000 | 0.000 | 0.000 | 0.000 | 0.000 | 0.000 | 0.000 | 0.000 |
| S17 | 0.000 | 0.000 | 0.000 | 0.000 | 26.967 | 0.000 | 0.000 | 0.000 | 20.645 | 20.453 | 0.000 | 0.000 | 0.000 | 0.000 |
| S18 | 0.000 | 0.000 | 0.000 | 0.000 | 0.000 | 0.000 | 0.000 | 0.000 | 0.000 | 0.000 | 0.000 | 0.000 | 0.000 | 0.000 |
| S19 | 0.000 | 0.000 | 16.678 | 0.000 | 0.000 | 0.000 | 0.000 | 0.000 | 178.312 | 152.395 | 358.130 | 18.035 | 0.000 | 0.000 |
| S20 | 0.000 | 0.000 | 0.000 | 0.000 | 62.888 | 0.000 | 0.000 | 0.000 | 109.410 | 110.052 | 0.000 | 0.000 | 0.000 | 0.000 |
| S21 | 0.000 | 0.000 | 0.000 | 0.000 | 12.392 | 13.127 | 0.000 | 0.000 | 221.341 | 98.922 | 0.000 | 16.076 | 0.000 | 0.000 |
| S22 | 0.000 | 0.000 | 0.000 | 0.000 | 24.479 | 0.000 | 0.000 | 0.000 | 213.785 | 124.886 | 0.000 | 15.485 | 0.000 | 0.000 |
| S23 | 0.000 | 0.000 | 0.000 | 0.000 | 16.569 | 12.183 | 0.000 | 0.000 | 197.336 | 138.190 | 0.000 | 21.637 | 0.000 | 0.000 |
| S24 | 0.000 | 0.000 | 0.000 | 0.000 | 26.247 | 0.000 | 0.000 | 0.000 | 57.944 | 51.684 | 0.000 | 0.000 | 0.000 | 0.000 |
| S25 | 0.000 | 0.000 | 0.000 | 0.000 | 0.000 | 0.000 | 0.000 | 0.000 | 367.056 | 175.091 | 0.000 | 36.855 | 0.000 | 0.000 |
| S26 | 0.000 | 0.000 | 0.000 | 0.000 | 0.000 | 0.000 | 0.000 | 0.000 | 152.987 | 48.417 | 0.000 | 0.000 | 0.000 | 0.000 |
| S27 | 0.000 | 0.000 | 0.000 | 0.000 | 0.000 | 0.000 | 0.000 | 0.000 | 151.754 | 68.169 | 0.000 | 0.000 | 0.000 | 0.000 |
| S28 | 0.000 | 0.000 | 0.000 | 0.000 | 0.000 | 17.949 | 0.000 | 0.000 | 392.698 | 149.150 | 0.000 | 34.845 | 0.000 | 0.000 |
| S29 | 0.000 | 0.000 | 0.000 | 0.000 | 0.000 | 17.853 | 0.000 | 0.000 | 294.516 | 132.536 | 0.000 | 29.353 | 0.000 | 0.000 |
| S30 | 0.000 | 0.000 | 0.000 | 0.000 | 0.000 | 24.148 | 27.737 | 0.000 | 409.650 | 117.800 | 0.000 | 26.255 | 0.000 | 0.000 |
| S31 | 0.000 | 0.000 | 0.000 | 0.000 | 34.989 | 13.878 | 0.000 | 11.101 | 184.278 | 157.266 | 0.000 | 16.501 | 0.000 | 0.000 |
| S32 | 0.000 | 0.000 | 0.000 | 0.000 | 71.759 | 15.846 | 0.000 | 0.000 | 312.990 | 105.728 | 0.000 | 32.592 | 0.000 | 0.000 |
| S33 | 0.000 | 0.000 | 0.000 | 0.000 | 0.000 | 0.000 | 0.000 | 0.000 | 129.551 | 67.821 | 0.000 | 0.000 | 0.000 | 0.000 |
| S34 | 0.000 | 0.000 | 0.000 | 0.000 | 26.069 | 0.000 | 0.000 | 0.000 | 176.324 | 141.160 | 0.000 | 24.975 | 0.000 | 0.000 |
| S35 | 0.000 | 0.000 | 0.000 | 0.000 | 29.313 | 0.000 | 0.000 | 0.000 | 313.460 | 163.960 | 0.000 | 29.462 | 0.000 | 0.000 |
| S36 | 0.000 | 0.000 | 0.000 | 0.000 | 26.441 | 0.000 | 0.000 | 0.000 | 183.788 | 79.048 | 0.000 | 14.400 | 0.000 | 0.000 |
| S37 | 0.000 | 0.000 | 0.000 | 0.000 | 16.729 | 12.618 | 0.000 | 0.000 | 266.329 | 125.612 | 0.000 | 26.280 | 0.000 | 0.000 |
| S38 | 0.000 | 0.000 | 0.000 | 0.000 | 12.729 | 14.657 | 0.000 | 0.000 | 214.363 | 120.557 | 0.000 | 14.930 | 0.000 | 0.000 |
| S39 | 0.000 | 0.000 | 0.000 | 0.000 | 11.456 | 15.711 | 0.000 | 0.000 | 309.241 | 103.246 | 0.000 | 25.932 | 0.000 | 0.000 |
| S40 | 0.000 | 0.000 | 0.000 | 0.000 | 34.763 | 0.000 | 0.000 | 0.000 | 52.344 | 22.209 | 0.000 | 0.000 | 0.000 | 0.000 |
| S41 | 0.000 | 0.000 | 0.000 | 0.000 | 14.856 | 0.000 | 0.000 | 0.000 | 175.277 | 69.328 | 0.000 | 13.791 | 0.000 | 0.000 |
| S42 | 0.000 | 0.000 | 0.000 | 0.000 | 0.000 | 13.886 | 0.000 | 19.031 | 325.341 | 158.246 | 0.000 | 28.668 | 0.000 | 0.000 |
| S43 | 0.000 | 0.000 | 0.000 | 0.000 | 0.000 | 65.193 | 0.000 | 0.000 | 564.309 | 170.649 | 0.000 | 55.057 | 0.000 | 0.000 |
| S44 | 0.000 | 0.000 | 0.000 | 0.000 | 0.000 | 0.000 | 0.000 | 0.000 | 174.334 | 181.510 | 0.000 | 21.020 | 0.000 | 0.000 |
| S45 | 19.539 | 34.041 | 0.000 | 11.777 | 0.000 | 0.000 | 0.000 | 0.000 | 199.917 | 183.442 | 503.343 | 21.344 | 0.000 | 0.000 |
| S46 | 0.000 | 0.000 | 0.000 | 0.000 | 79.384 | 0.000 | 0.000 | 0.000 | 78.891 | 57.278 | 0.000 | 0.000 | 0.000 | 0.000 |
| S47 | 0.000 | 0.000 | 0.000 | 0.000 | 49.097 | 0.000 | 0.000 | 0.000 | 56.508 | 27.305 | 0.000 | 0.000 | 0.000 | 0.000 |
| S48 | 0.000 | 0.000 | 0.000 | 0.000 | 39.442 | 0.000 | 0.000 | 0.000 | 15.291 | 14.447 | 0.000 | 0.000 | 0.000 | 0.000 |
| S49 | 0.000 | 0.000 | 0.000 | 0.000 | 62.417 | 0.000 | 0.000 | 0.000 | 143.181 | 72.154 | 0.000 | 0.000 | 0.000 | 0.000 |
| S50 | 0.000 | 0.000 | 0.000 | 0.000 | 25.314 | 14.997 | 0.000 | 0.000 | 179.545 | 71.041 | 0.000 | 0.000 | 39.048 | 52.237 |
| S51 | 0.000 | 0.000 | 0.000 | 0.000 | 31.435 | 0.000 | 0.000 | 0.000 | 156.763 | 61.608 | 0.000 | 0.000 | 0.000 | 24.154 |

Note: “RT” followed by a number indicates the retention time and its rank order among the retention times of 86 peak areas. Peak areas are expressed in arbitrary units (mAU·s). “0” indicates that the peak was not detected or its area was below the integration threshold. Samples S1–S51 correspond to different plant parts and geographical origins as described in Table 1.

## **Supplementary Table S2. Assignment of cross-validation groups for each sample.**

| **Batch No.** | **Part** | **Group** | **CV_Fold** | **Batch No.** | **Part** | **Group** | **CV_Fold** |
| --- | --- | --- | --- | --- | --- | --- | --- |
| S1 | Root | Group1 | 1 | S27 | Leaf | Group2 | 3 |
| S2 | Root | Group1 | 2 | S28 | Leaf | Group2 | 4 |
| S3 | Root | Group1 | 3 | S29 | Leaf | Group2 | 5 |
| S4 | Root | Group1 | 4 | S30 | Leaf | Group2 | 6 |
| S5 | Root | Group1 | 5 | S31 | Leaf | Group2 | 7 |
| S6 | Root | Group1 | 7 | S32 | Leaf | Group2 | 1 |
| S7 | Old branch | Group1 | 1 | S33 | Leaf | Group2 | 2 |
| S8 | Old branch | Group1 | 2 | S34 | Leaf | Group2 | 3 |
| S9 | Old branch | Group1 | 3 | S35 | Leaf | Group2 | 4 |
| S10 | Old branch | Group1 | 4 | S36 | Leaf | Group2 | 5 |
| S11 | Old branch | Group1 | 5 | S37 | Leaf | Group2 | 6 |
| S12 | Old branch | Group1 | 6 | S38 | Leaf | Group2 | 7 |
| S13 | Young branch | Group1 | 1 | S39 | Leaf | Group2 | 1 |
| S14 | Young branch | Group1 | 2 | S40 | Leaf | Group2 | 2 |
| S15 | Young branch | Group1 | 3 | S41 | Leaf | Group2 | 3 |
| S16 | Young branch | Group1 | 4 | S42 | Leaf | Group2 | 4 |
| S17 | Young branch | Group1 | 5 | S43 | Leaf | Group2 | 5 |
| S18 | Young branch | Group1 | 7 | S44 | Leaf | Group2 | 6 |
| S19 | Young branch and leaf | Group2 | 1 | S45 | Leaf | Group2 | 7 |
| S20 | Young branch and leaf | Group2 | 2 | S46 | Fruit | Group2 | 1 |
| S21 | Young branch and leaf | Group2 | 3 | S47 | Fruit | Group2 | 2 |
| S22 | Young branch and leaf | Group2 | 4 | S48 | Fruit | Group2 | 3 |
| S23 | Young branch and leaf | Group2 | 5 | S49 | Flower | Group3 | 0 |
| S24 | Young branch and leaf | Group2 | 7 | S50 | Flower | Group3 | 0 |
| S25 | Leaf | Group2 | 1 | S51 | Flower | Group3 | 0 |
| S26 | Leaf | Group2 | 2 |  |  |  |  |

Note: CV_Fold = 0 indicates samples permanently assigned to the training set in each iteration (flower group, n = 3, due to limited sample size). CV_Fold = 1–7 indicates the fold in which the sample served as the test set. Group 1: root, old branch, young branch; Group 2: young branch and leaf, leaf, fruit; Group 3: flower. This stratified 7-fold cross-validation strategy was designed to evaluate the predictive performance of the OPLS-DA model while accommodating the limited sample size of the flower group.

*t*/min

A

*t*/min

B

*t*/min

C

RT83

D

*t*/min

RT83

E

*t*/min

F

*t*/min

G

*t*/min

## **Supplementary Figure S1.**

## HPLC fingerprints of 51 batches of R. tomentosum samples, with time (min) on the x-axis and signal intensity (mAU) on the y-axis. A: Roots (batches S1–S6) and their reference chromatogram; B: Old branches (batches S7–S12) and their reference chromatogram; C: Young branches (batches S13–S18) and their reference chromatogram; D: Branches and leaves (batches S19–S24) and their reference chromatogram; E: Leaves (batches S25–S45) and their reference chromatogram; F: Fruits (batches S46–S48) and their reference chromatogram; G: Flowers (batches S49–S51) and their reference chromatogram. The reference chromatogram for each group was generated by averaging the chromatograms of all samples within that group.

## **Supplementary Table S3.** Similarity evaluation of 51 batches of *R. tomentosum* sample.

| **Batch No.** | **Similarity** | **Group** | **Batch No.** | **Similarity** | **Group** | **Batch No.** | **Similarity** | **Group** |
| --- | --- | --- | --- | --- | --- | --- | --- | --- |
| S1 | 1 | A | S18 | 0.981 | C | S35 | 0.99 | E |
| S2 | 1 | A | S19 | 0.989 | D | S36 | 0.941 | E |
| S3 | 1 | A | S20 | 0.979 | D | S37 | 0.985 | E |
| S4 | 0.999 | A | S21 | 0.983 | D | S38 | 0.997 | E |
| S5 | 0.999 | A | S22 | 0.979 | D | S39 | 0.989 | E |
| S6 | 0.999 | A | S23 | 0.99 | D | S40 | 0.615 | E |
| S7 | 0.995 | B | S24 | 0.99 | D | S41 | 0.931 | E |
| S8 | 0.999 | B | S25 | 0.964 | E | S42 | 0.989 | E |
| S9 | 0.998 | B | S26 | 0.889 | E | S43 | 0.974 | E |
| S10 | 0.99 | B | S27 | 0.987 | E | S44 | 0.93 | E |
| S11 | 0.999 | B | S28 | 0.958 | E | S45 | 0.983 | E |
| S12 | 0.994 | B | S29 | 0.956 | E | S46 | 0.996 | F |
| S13 | 0.97 | C | S30 | 0.939 | E | S47 | 0.965 | F |
| S14 | 0.989 | C | S31 | 0.926 | E | S48 | 0.929 | F |
| S15 | 0.995 | C | S32 | 0.977 | E | S49 | 0.999 | G |
| S16 | 0.986 | C | S33 | 0.982 | E | S50 | 0.999 | G |
| S17 | 0.979 | C | S34 | 0.986 | E | S51 | 0.999 | G |

Note: A: root; B: old branch; C: young branch; D: young branch and leaf; E: leaf; F: fruit; G: flower.

## **Supplementary Table S4. Similarity of the mean reference chromatogram to individual chromatograms within each plant part group, and the percentage of non-common peak areas.**

| **Group** | **Similarity** | **Non-common peak area** | **Total peak area** | **Percentage of non-common peak area (%)** |
| --- | --- | --- | --- | --- |
| A: Root | 0.976 | 182.1 | 2644.88 | 6.88 |
| B: Old branch | 0.989 | 347.98 | 2918.08 | 11.92 |
| C: Young branch | 0.997 | 880.16 | 3093.5 | 28.45 |
| D: Young branch and leaf | 0.967 | 2912.17 | 4010.42 | 72.62 |
| E: Leaf | 0.662 | 2419.47 | 3027.2 | 79.92 |
| F: Fruit | 0.544 | 980.35 | 1209.34 | 81.06 |
| G: Flower | 0.792 | 6665.62 | 7496.55 | 88.92 |

Note: Similarity values represent the mean similarity between the group reference chromatogram and each individual chromatogram within the same group. Non-common peak area refers to the total area of peaks that are not present in all chromatograms within the same group. The percentage of non-common peak area was calculated as (non-common peak area / total peak area) × 100%.

## **Supplementary Figure S2.**

## UHPLC-HRMS base peak ion (BPC) chromatograms and UHPLC-UV chromatograms of R. tomentosum samples S45 and S44, with time (min) on the x-axis and ion intensity on the y-axis. A: S44 UHPLC-HRMS BPC, negative ion mode; B: S44 UHPLC-HRMS BPC, positive ion mode; C: S44 UHPLC-UV chromatogram at 360 nm; D: S45 UHPLC-HRMS BPC, negative ion mode; E: S45 UHPLC-HRMS BPC, positive ion mode; F: S45 UHPLC-UV chromatogram at 360 nm. Peak 63 corresponds to the same compound as peak RT83 in **Supplementary Figure S1**.

## **Supplementary Table S5. Identification results of components (predominantly flavonoids) in *R. tomentosum* samples.**

| Peak No. | Retention time (min) | Adduct ion | Observed m/z | Theoretical m/z | Error (ppm) | Molecular formula | Exact mass | Compound name | Fragment ions (MS/MS) | Identification approach |
| --- | --- | --- | --- | --- | --- | --- | --- | --- | --- | --- |
| 1 | 2.56 | [M-H]^-^ | 191.0570 | 191.0561 | 4.7 | C_7_H_12_O_6_ | 192.06 | Quinic acid | 127.0397;109.0290;93.0345;85.0296 | HRMSB |
| 2 | 2.80 | [M-H]^-^ | 337.0781 | 337.0776 | 1.5 | C_12_H_18_O_11_ | 338.08 | 2-O-β-D-Glucopyranosyl-L-ascorbic acid | 337.0774;277.0559;174.0164;113.9945 | MSFP |
| 3 | 3.54 | [M-H]^-^ | 191.0199 | 191.0197 | 1.0 | C_6_H_8_O_7_ | 192.03 | Citric acid | 111.0094;87.0098;85.0307;67.0200 | HRMSB |
| 4 | 3.98 | [M+H]^+^ | 268.1052 | 268.1040 | 4.5 | C_10_H_13_N_5_O_4_ | 267.10 | Adenosine | 268.1004;136.0623;119.0356 | HRMSB |
| 5 | 4.62 | [M-H]^-^ | 331.0668 | 331.0665 | 0.9 | C_13_H_16_O_10_ | 332.07 | 1-O-Galloyl-β-D-glucose | 331.0668;271.0458;169.0135;151.0032;125.0239 | HRMSB (Pawlowska et al., 2022) |
| 6 | 5.87 | [M-H]^-^ | 331.0670 | 331.0665 | 1.5 | C_13_H_16_O_10_ | 332.07 | 6-O-Galloyl-D-glucose | 331.0666;313.0549;168.0061;149.9954;125.0239 | MSFP |
| 7 | 6.25 | [M-H]^-^ | 865.1978 | 865.1985 | -0.8 | C_45_H_38_O_18_ | 866.21 | Procyanidin C1 | 695.1397;575.1190;407.0754;287.0546;125.0229 | HRMSB |
| 8 | 6.85 | [M-H]^-^ | 315.0732 | 315.0722 | 3.2 | C_13_H_16_O_9_ | 316.08 | Protocatechuic acid 3-O-glucoside | 315.0697;152.0117;108.0224 | MSFP |
| 9 | 7.09 | [M-H]^-^ | 575.1191 | 575.1195 | -0.7 | C_30_H_24_O_12_ | 576.13 | Procyanidin A4 | 575.1238;449.0896;407.0794;309.0406;125.0235 | MSFP |
| 10 | 7.56 | [M-H]^-^ | 315.1082 | 315.1085 | -1.0 | C_14_H_20_O_8_ | 316.12 | Hydroxytyrosol 1-O-glucoside | 153.0555;123.0451 | MSFP |
| 11 | 7.71 | [M-H]^-^ | 305.0663 | 305.0667 | -1.3 | C_15_H_14_O_7_ | 306.07 | Gallocatechin | 305.0650;219.0660;179.0338;137.0241;125.0239 | HRMSB (Jaiswal et al., 2012) |
| 12 | 7.89 | [M-H]^-^ | 299.0768 | 299.0772 | -1.3 | C_13_H_16_O_8_ | 300.08 | p-Hydroxybenzoic acid glucoside | 299.0741;137.0239;59.0131 | MSFP |
| 13 | 8.10 | [M-H]^-^ | 451.1248 | 451.1246 | 0.4 | C_21_H_24_O_11_ | 452.13 | Catechin 3-O-glucoside | 451.1222;289.0715;245.0815;203.0708;137.0242 | MSFP |
| 14 | 8.86 | [M-H]^-^ | 353.0893 | 353.0878 | 4.2 | C_16_H_18_O_9_ | 354.10 | Neochlorogenic acid | 353.0828;191.0563;179.0351;135.0459 | HRMSB |
| 15 | 9.21 | [M-H]^-^ | 451.1262 | 451.1246 | 3.5 | C_21_H_24_O_11_ | 452.13 | Catechin 7-O-glucoside | 451.1224;289.0722;245.0815;203.0722;109.0294 | MSFP |
| 16 | 9.81 | [M-H]^-^ | 341.0866 | 341.0878 | -3.5 | C_15_H_18_O_9_ | 342.10 | Caffeic acid 4-O-β-D-glucopyranoside | 179.0344;161.0239;133.0291 | MSFP |
| 17 | 10.30 | [M-H]^-^ | 339.0736 | 339.0716 | 5.9 | C_15_H_16_O_9_ | 340.08 | Aesculin | 339.0716;177.0194;133.0288;105.0336 | HRMSB (Razgonova  et al., 2023) |
| 18 | 10.65 | [M-H]^-^ | 577.1365 | 577.1352 | 2.3 | C_30_H_26_O_12_ | 578.14 | Procyanidin B3 | 451.1386;407.0788;289.0724;125.0234 | MSFP (Jaiswal et al., 2012) |
| 19 | 10.75 | [M-H]^-^ | 359.1343 | 359.1348 | -1.4 | C_16_H_24_O_9_ | 360.14 | Piperoside | 359.1308;197.0813;182.0578;137.0239 | MSFP |
| 20 | 11.44 | [M-H]^-^ | 305.0653 | 305.0667 | -4.6 | C_15_H_14_O_7_ | 306.07 | Epigallocatechin | 305.0638;219.0650;179.0347;137.0232;125.0237 | HRMSB (Jaiswal et al., 2012) |
| 21 | 11.99 | [M-H]^-^ | 577.1363 | 577.1352 | 1.9 | C_30_H_26_O_12_ | 578.14 | Procyanidin B5 | 577.1334;407.0766;289.0714;125.0240 | HRMSB |
| 22 | 12.78 | [M-H]^-^ | 341.0889 | 341.0878 | 3.2 | C_15_H_18_O_9_ | 342.10 | Caffeic acid-3-O-β-D-glucoside | 179.0345;161.0220;135.0443 | MSFP |
| 23 | 13.43 | [M-H]^-^ | 289.0715 | 289.0718 | -1.0 | C_15_H_14_O_6_ | 290.08 | (+)-Catechin | 289.0715;245.0817;203.0707;123.0443;109.0286 | HRMSB |
| 24 | 13.43 | [M-H]^-^ | 353.0879 | 353.0878 | 0.3 | C_16_H_18_O_9_ | 354.10 | Chlorogenic acid | 191.0558;161.0232;127.0391 | HRMSB |
| 25 | 14.44 | [M-H]^-^ | 353.0874 | 353.0878 | -1.1 | C_16_H_18_O_9_ | 354.10 | Cryptochlorogenic acid | 191.0551;179.0340;173.0451;135.0443 | HRMSB |
| 26 | 15.71 | [M-H]^-^ | 177.0190 | 177.0188 | 1.1 | C_9_H_6_O_4_ | 178.03 | Esculetin | 177.0189;133.0296;105.0350;89.0402 | HRMSB (Wang et al., 2018) |
| 27 | 15.71 | [M-H]^-^ | 577.1349 | 577.1352 | -0.5 | C_30_H_26_O_12_ | 578.14 | Procyanidin B4 | 577.1305;407.0750;289.0706;125.0245 | MSFP |
| 28 | 16.38 | [M-H]^-^ | 369.0838 | 369.0822 | 4.3 | C_16_H_18_O_10_ | 370.09 | Fraxin | 369.0801;207.0296;192.0063;163.0035 | HRMSB |
| 29 | 16.85 | [M-H]^-^ | 355.1031 | 355.1035 | -1.1 | C_16_H_20_O_9_ | 356.11 | Feruloyl-β-D-glucopyranoside | 193.0496;175.0396;160.0161;132.0212 | MSFP |
| 30 | 19.97 | [M-H]^-^ | 577.1355 | 577.1352 | 0.5 | C_30_H_26_O_12_ | 578.14 | Procyanidin B2 | 577.1339;407.0776;289.0716;125.0242 | HRMSB |
| 31 | 20.41 | [M-H]^-^ | 289.0728 | 289.0718 | 3.5 | C_15_H_14_O_6_ | 290.08 | (-)-Epicatechin | 289.0707;245.0812;203.0704;123.0446;109.0291 | HRMSB |
| 32 | 20.79 | [M-H]^-^ | 329.0872 | 329.0878 | -1.8 | C_14_H_18_O_9_ | 330.10 | Myrciaphenone A | 329.0834;191.0343;167.0347;123.0453 | MSFP (Ye et al., 2020) |
| 33 | 21.79 | [M+H]^+^ | 209.0459 | 209.0450 | 4.3 | C_10_H_8_O_5_ | 208.04 | Fraxetin | 209.0459;194.0223;166.0273;149.0245;92.0268 | HRMSB |
| 34 | 23.76 | [M-H]^-^ | 465.1048 | 465.1039 | 1.9 | C_21_H_22_O_12_ | 466.11 | Taxifolin 3-O-glucoside | 465.1013;285.0398;151.0037;125.0242 | MSFP |
| 35 | 26.64 | [M-H]^-^ | 465.1019 | 465.1039 | -4.3 | C_21_H_22_O_12_ | 466.11 | Taxifolin 7-O-glucoside | 465.0977;285.0386;151.0034;125.0243 | MSFP |
| 36 | 27.98 | [M-H]^-^ | 483.1139 | 483.1144 | -1.0 | C_21_H_24_O_13_ | 484.12 | 6-[[6-O-(4-carboxy-3-hydroxy-3-methyl-1-oxobutyl)-β-D-glucopyranosyl]oxy]-7-hydroxy-2H-1-Benzopyran-2-one | 483.1127;381.0816;339.0711;177.0187 | MSFP (Dubois et al., 1990) |
| 37 | 32.85 | [M-H]^-^ | 575.1192 | 575.1195 | -0.5 | C_30_H_24_O_12_ | 576.13 | Procyanidin A1 | 575.1177;539.0963;449.0861;285.0395;125.0236 | MSFP |
| 38 | 34.01 | [M-H]^-^ | 449.1058 | 449.1089 | -6.9 | C_21_H_22_O_11_ | 450.12 | Kaempferol hexoside | 449.1058;287.0540;269.0444;180.00855;151.0032 | MSFP (Bekkai et al., 2022) |
| 39 | 35.25 | [M-H]^-^ | 465.1024 | 465.1039 | -3.2 | C_21_H_22_O_12_ | 466.11 | Taxifolin 3'-O-glucoside | 465.0981;285.0383;151.0035;125.0242 | MSFP (Bennini et al., 1993) |
| 40 | 37.39 | [M+Cl]^-^ | 425.1012 | 425.1004 | 1.9 | C_20_H_22_O_8_ | 390.13 | Resveratrol 3-O-β-D-glucoside | 389.1215;327.1345;227.0704;185.0598 | HRMSB |
| 41 | 43.15 | [M-H]^-^ | 463.0908 | 463.0882 | 5.6 | C_21_H_20_O_12_ | 464.10 | Hyperoside | 463.0881;300.0275;271.0245;255.0292;151.0026 | HRMSB |
| 42 | 45.32 | [M-H]^-^ | 463.0889 | 463.0882 | 1.5 | C_21_H_20_O_12_ | 464.10 | Procyanidin A2 | 463.0870;300.0267;271.0239;255.0289;151.0028 | HRMSB |
| 43 | 45.40 | [M-H]^-^ | 575.1199 | 575.1195 | 0.7 | C_30_H_24_O_12_ | 576.13 | Isoquercitrin | 575.1184;539.0963;449.0863;285.0395;125.0240 | MSFP |
| 44 | 48.82 | [M-H]^-^ | 433.0770 | 433.0776 | -1.4 | C_20_H_18_O_11_ | 434.08 | Quercetin 3-O-arabinoside | 433.0761;300.0265;271.0239;255.0291;151.0024 | MSFP (Saleem et al., 2010) |
| 45 | 51.07 | [M-H]^-^ | 433.0780 | 433.0776 | 0.9 | C_20_H_18_O_11_ | 434.08 | Guaijaverin | 433.0774;300.0273;271.0243;255.0290;151.0023 | HRMSB (Simom et al., 1993) |
| 46 | 51.81 | [M-H]^-^ | 447.0918 | 447.0933 | -3.4 | C_21_H_20_O_11_ | 448.10 | Kaempferol 7-O-β-D-glucoside | 447.0924;284.0319;255.0292;227.0341;151.0021 | HRMSB |
| 47 | 54.43 | [M-H]^-^ | 433.0794 | 433.0776 | 4.2 | C_20_H_18_O_11_ | 434.08 | Avicularin | 433.0740;300.0264;271.0237;255.0287;151.0036 | HRMSB (Lhuillier et al., 2007) |
| 48 | 56.62 | [M-H]^-^ | 447.0933 | 447.0933 | 0.0 | C_21_H_20_O_11_ | 448.10 | Quercitrin | 447.0917;300.0265;271.0236;255.0290;151.0028 | HRMSB |
| 49 | 58.29 | [M-H]^-^ | 449.0712 | 449.0725 | -2.9 | C_20_H_18_O_12_ | 450.08 | Myricetin 3-O-arabinoside | 317.0296;178.9980;151.0034;137.0236 | MSFP |
| 50 | 63.79 | [M-H]^-^ | 505.0961 | 505.0988 | -5.3 | C_23_H_22_O_13_ | 506.11 | Quercetin 3-O-(2-O-acetyl-β-D-galactopyranoside) | 505.0972;300.0270;271.0241;255.0289;151.0028 | MSFP |
| 51 | 66.27 | [M-H]^-^ | 693.2019 | 693.2031 | -1.7 | C_32_H_38_O_17_ | 694.21 | 3,6'-diferuloylsucrose | 693.2010;517.1541;337.0914;175.0395 | MSFP |
| 52 | 67.57 | [M-H]^-^ | 583.1102 | 583.1093 | 1.5 | C_28_H_24_O_14_ | 584.12 | 2-(3,4-Dihydroxyphenyl)-5,7-dihydroxy-3-[[6-O-(4-hydroxybenzoyl)-β-D-galactopyranosyl]oxy]-4H-1-benzopyran-4-one | 583.1127;463.0893;300.0281;151.0025 | MSFP |
| 53 | 70.56 | [M-H]^-^ | 625.1177 | 625.1199 | -3.5 | C_30_H_26_O_15_ | 626.13 | Quercetin 3-O-(6''-caffeoyl)-β-D-glucopyranoside | 625.1183;463.0862;301.0334;151.0024 | MSFP |
| 54 | 71.39 | [M-H]^-^ | 475.0886 | 475.0882 | 0.8 | C_22_H_20_O_12_ | 476.10 | 5,7,8-Dihydroxy-6-methoxyflavone 7-O-glucuronide | 475.0869;300.0267;271.0243;151.0028 | MSFP |
| 55 | 71.64 | [M-H]^-^ | 505.1004 | 505.0988 | 3.2 | C_23_H_22_O_13_ | 506.11 | Quercetin 3-O-(6-O-acetyl-β-D-glucopyranoside) | 505.0987;300.0254;271.0226;255.0268;150.9999 | MSFP (Louis et al., 2010) |
| 56 | 73.18 | [M-H]^-^ | 505.0986 | 505.0988 | -0.4 | C_23_H_22_O_13_ | 506.11 | Quercetin 3-O-(2''-acetyl)-β-D-glucopyranoside | 505.0986;300.0277;271.0245;255.0295;151.0025 | MSFP |
| 57 | 73.76 | [M-H]^-^ | 577.1597 | 577.1622 | -4.3 | C_27_H_30_O_14_ | 578.16 | 2-(3,4-Dihydroxyphenyl)-3,5,7-trihydroxy-6-[[6-O-(1-oxohexyl)-β-D-glucopyranosyl]oxy]-4H-1-benzopyran-4-one | 577.1597;463.0898;300.0280;271.0245;151.0020 | MSFP |
| 58 | 77.61 | [M-H]^-^ | 475.0875 | 475.0882 | -1.5 | C_22_H_20_O_12_ | 476.10 | 5,6,7-Trihydroxy-8-methoxyflavone 7-O-glucuronide | 475.0869;300.0270;271.0240;151.0023 | MSFP |
| 59 | 79.74 | [M-H]^-^ | 475.0866 | 475.0882 | -3.4 | C_22_H_20_O_12_ | 476.10 | 3-[(5-O-Acetyl-α-L-arabinofuranosyl)oxy]-2-(3,4-dihydroxyphenyl)-5,7-dihydroxy-4H-1-benzopyran-4-one | 475.0866;300.0268;271.0236;151.0020 | MSFP |
| 60 | 84.14 | [M-H]^-^ | 609.1225 | 609.1250 | -4.1 | C_30_H_26_O_14_ | 610.13 | Quercetin 3-O-(6-p-coumaroyl)-β-galactoside | 609.1199;463.0843;300.0259;151.0032 | MSFP |
| 61 | 86.04 | [M-H]^-^ | 609.1246 | 609.1250 | -0.7 | C_30_H_26_O_14_ | 610.13 | Quercetin 3-O-(6"-O-p-coumaroyl)-glucoside | 609.1216;463.0856;300.0264;163.0384;151.0030 | MSFP |
| 62 | 87.63 | [M-H]^-^ | 639.1374 | 639.1355 | 3.0 | C_31_H_28_O_15_ | 640.14 | Quercetin 3-O-(6''-feruloyl)-β-D-galactopyranoside | 639.1350;463.0873;300.0271;193.0480;151.0028 | MSFP |
| 63 | 90.55 | [M-H]^-^ | 547.1088 | 547.1093 | -0.9 | C_25_H_24_O_14_ | 548.12 | 3-[(3,6-di-O-acetyl-β-D-galactopyranosyl)oxy]-2-(3,4-dihydroxyphenyl)-5,7-dihydroxy-4H-1-benzopyran-4-one | 547.1090;300.0274;271.0243;151.0028 | MSFP (Chosson et al., 1998) |
| 64 | 91.79 | [M-H]^-^ | 609.1229 | 609.1250 | -3.4 | C_30_H_26_O_14_ | 610.13 | Quercetin 3-O-(3'-O-p-coumaroyl)-glucoside | 609.1229;463.0867;300.0266;163.0025;151.0025 | MSFP |
| 65 | 104.68 | [M-H]^-^ | 283.0614 | 283.0612 | 0.7 | C_16_H_12_O_5_ | 284.07 | Galangin 3-methyl ether | 283.0593;268.0368;240.0413;211.0392 | HRMSB (Wollenweber et al., 1984) |

Note: HRMSB: identified by high-resolution mass spectral database matching; MSFP: identified by MS fragmentation pattern matching. References in parentheses indicate literature-based confirmation of the compound identification.

**Supplementary Figure S3.**

Representative mass spectra of the compound corresponding to peak 63 (RT83) , with m/z on the x-axis and ion intensity on the y-axis. A: Full-scan MS spectrum (negative ion mode) at retention time 90.554 min showing the deprotonated molecular ion at *m/z* 547.1088; B: Product ion spectrum (MS/MS) of the precursor ion at *m/z* 547.1 (collision-induced dissociation, CID), showing characteristic fragment ions including *m/z* 151.0029, 199.0382, 271.0241, 300.0272, 325.0332, 445.0754, and 505.0972.

## **Supplementary Figure S4. HPLC chromatograms of *R. tomentosum* samples and mixed reference standards.**

## A: *L. palustre* var. *dilatatum* (batch S45); B: *L. palustre* var. *palustre* (batch S44); C: Mixed reference standards. Peaks are labeled as follows: RT7, neochlorogenic acid; RT10, aesculin; RT15, chlorogenic acid; RT19, esculetin; RT21, fraxin; RT27, fraxetin; RT40, polydatin; RT43, hyperoside; RT44, rutin; RT46, isoquercitrin; RT49, quercetin 3-O-arabinoside; RT50, avicularin; RT52, guaijaverin; RT54, quercitrin; RT63, 2-(3,4-dihydroxyphenyl)-5,7-dihydroxy-3-[[6-O-(4-hydroxybenzoyl)-β-D-galactopyranosyl]oxy]-4H-1-benzopyran-4-one; RT81, quercetin 3-O-(6''-O-p-coumaroyl)-glucoside; RT82, quercetin-3-O-(6''-feruloyl)-β-D-galactopyranoside; RT83, 3-[(3,6-di-O-acetyl-β-D-galactopyranosyl)oxy]-2-(3,4-dihydroxyphenyl)-5,7-dihydroxy-4H-1-benzopyran-4-one.

## **Supplementary Figure S5.**

## Permutation test plot (n = 200) for the 3-class OPLS-DA model.The permutation test was performed to evaluate the robustness of the OPLS-DA model and to exclude the possibility of overfitting. Blue circles represent R² values; green squares represent Q² values obtained from 200 random permutations of class labels. The regression lines indicate the trends of R² and Q² as a function of the correlation between the original and permuted class assignments. The Q² regression line exhibited a negative y-intercept (–0.485), confirming that the model’s predictive performance was not due to random chance. Arrows indicate the R² and Q² values of the original model (R² = 0.945, Q² = 0.855).

**Supplementary Table S6. Method validation parameters for the seven bioactive compounds.**

| **Compound** | **Regression equation** | **R²** | **Linear range (ng/mL)** | **LOD (ng/mL)** | **LOQ (ng/mL)** | **Precision RSD (%)** | **Repeatability RSD (%)** | **Stability RSD (%)** | **Recovery (%)** | **Recovery RSD (%)** |
| --- | --- | --- | --- | --- | --- | --- | --- | --- | --- | --- |
| Aesculin | y = 8541.1x + 4496 | 0.9999 | 2.59–83.02 | 0.78 | 2.59 | 0.40 | 1.41 | 0.36 | 106.03 | 1.90 |
| Fraxin | y = 10389x + 23531 | 0.9999 | 10.53–337.18 | 3.19 | 10.53 | 0.21 | 1.15 | 0.39 | 115.35 | 1.20 |
| Hyperoside | y = 24069x + 10924 | 0.9999 | 3.25–104 | 0.98 | 3.25 | 0.1 | 1.37 | 0.10 | 95.87 | 4.40 |
| Isoquercitrin | y = 14480x − 2272.8 | 0.9999 | 1.68–54 | 0.51 | 1.68 | 0.68 | 1.69 | 0.68 | 95.83 | 4.10 |
| Avicularin | y = 15816x − 1207.9 | 0.9999 | 2.84–91.14 | 0.86 | 2.84 | 0.57 | 2.51 | 0.57 | 108.25 | 5.82 |
| Guaijaverin | y = 7533.2x − 391.27 | 0.9997 | 4.29–137.58 | 1.3 | 4.29 | 0.27 | 3.72 | 0.27 | 100.46 | 5.25 |
| Quercitrin | y = 16831x + 9833 | 0.9999 | 8.46–270.8 | 2.56 | 8.46 | 1.37 | 1.10 | 2.71 | 100.18 | 6.36 |

Note: Precision was assessed by six consecutive injections of the reference standard solution; repeatability was assessed by six independently prepared sample solutions; stability was assessed at 0, 2, 4, 6, 8, 12, and 24 h; recovery was assessed by spiked recovery experiments at three concentration levels with three replicates each (n = 9).

**Supplementary Table S7. Method validation parameters for the eight elements.**

| **Element** | **Calibration curve** | **R** | **LOD (μg/L)** | **LOQ (μg/L)** | **Recovery (%)** | **Recovery RSD (%)** | **Repeatability RSD (%)** | **Intermediate precision RSD (%)** | **Stability RSD (%)** |
| --- | --- | --- | --- | --- | --- | --- | --- | --- | --- |
| Mg | y = 2.7495x − 9.1459×10⁻⁴ | 0.9999 | 0.2859 | 0.8577 | 93.31 | 3.45 | 0.42 | 1.97 | 1.12 |
| Al | y = 0.0015x + 8.1815×10⁻⁴ | 0.9999 | 0.2785 | 0.8355 | 92.62 | 5.49 | 0.51 | 1.70 | 0.76 |
| K | y = 3.8628x − 0.0055 | 0.9999 | 2.809 | 8.427 | 88.81 | 4.56 | 0.46 | 2.39 | 0.78 |
| Ca | y = 0.0101x − 1.4947×10⁻⁵ | 0.9999 | 0.0403 | 0.1209 | 99.07 | 4.29 | 0.46 | 1.40 | 1.84 |
| Mn | y = 0.0239x − 0.0119 | 1.0000 | 0.1109 | 0.3327 | 88.10 | 4.48 | 0.39 | 2.35 | 0.97 |
| Fe | y = 0.0312x − 0.0052 | 0.9999 | 0.1207 | 0.3621 | 101.04 | 4.21 | 0.55 | 1.89 | 1.30 |
| Cu | y = 0.0545x − 6.7523×10⁻⁵ | 1.0000 | 0.03817 | 0.1145 | 90.78 | 5.48 | 0.60 | 2.38 | 0.84 |
| Zn | y = 0.0104x + 6.5574×10⁻⁴ | 0.9999 | 0.1737 | 0.5211 | 91.53 | 8.84 | 0.46 | 3.57 | 0.74 |

Note: Repeatability was assessed by analyzing six independently prepared sample solutions; intermediate precision was assessed on three consecutive days; stability was assessed at 0, 2, 4, 6, 8, 12, and 24 h. All validation parameters met the acceptance criteria, confirming the method’s suitability for the quantification of elements in *R. tomentosum*. Acceptance criteria: recovery within 85%–110% (or 80%–115% for Cu and Zn); RSD ≤ 5% for precision/ repeatability, ≤ 10% for recovery.

## Supplementary references

Bekkai, D., Oulad El Majdoub, Y., Bekkai, H., Cacciola, F., Miceli, N., and Taviano, M. F., et al. (2022). Determination of the Phenolic Profile by Liquid Chromatography, Evaluation of Antioxidant Activity and Toxicity of Moroccan *Erica multiflora*, *Erica scoparia*, and *Calluna vulgaris* (Ericaceae). *Molecules* 27, 3979. doi: 10.3390/molecules27133979

Bennini, B., Chulia, A. J., and Kaouadji, M. (1993). (2R,3R)-Dihydroflavonol aglycone and glycosides from *Erica cinerea*. *Phytochemistry* 33, 1233–1236. doi: 10.1016/0031-9422(93)85055-v

Chosson, E., Chaboud, A., Chulia, A. J., and Raynaud, J. (1998). Dihydroflavonol glycosides from *Rhododendron ferrugineum*. *Phytochemistry* 49, 1431–1433. doi: 10.1016/S0031-9422(98)00080-6

Dubois, M.-A., Wierer, M., and Wagner, H. (1990). Palustroside, a coumarin glucoside ester from *Ledum palustre*. *Phytochemistry* 29, 3369–3371. doi: 10.1016/0031-9422(90)80221-2

Jaiswal, R., Jayasinghe, L., and Kuhnert, N. (2012). Identification and characterization of proanthocyanidins of 16 members of the *Rhododendron* genus (Ericaceae) by tandem LC-MS. ***J.*** *Mass Spectrom.* 47, 502–515. doi: 10.1002/jms.2954

Lhuillier, A., Fabre, N., Moyano, F., Martins, N., Claparols, C., and Fourasté, I., et al. (2007). Comparison of flavonoid profiles of *Agauria salicifolia* (Ericaceae) by liquid chromatography-UV diode array detection-electrospray ionisation mass spectrometry. *J. Chromatogr. A* 1160, 13–22. doi: 10.1016/j.chroma.2007.03.038

Louis, A., Petereit, F., Lechtenberg, M., Deters, A,. and Hensel, A. (2010). Phytochemical characterization of *Rhododendron ferrugineum* and in vitro assessment of an aqueous extract on cell toxicity. *Planta Med.* 76, 1550–1557. doi: 10.1055/s-0029-1241016

Pawlowska, A. M., De Leo, M., and Braca, A. (2006). Phenolics of *Arbutus unedo* L. (Ericaceae) Fruits: Identification of Anthocyanins and Gallic Acid Derivatives. *J. Agric. Food Chem.* 54, 10234–10238. doi: 10.1021/jf062230o

Razgonova, M. P., Zakharenko, A. M., and Golokhvast, K. S. (2023). Investigation of the supercritical CO₂ extracts of wild *Ledum palustre* L. (*Rhododendron tomentosum* Harmaja) and identification of its metabolites by tandem mass spectrometry. *Russ. J. Bioorg. Chem.* 49, 1645–1657. doi: 10.1134/S1068162023070889

Saleem, A., Harris, C. S., Asim, M., Cuerrier, A., Martineau, L., and Haddad, P. S., et al. (2010). A RP-HPLC-DAD-APCI/MSD method for the characterisation of medicinal Ericaceae used by the Eeyou Istchee Cree First Nations. *Phytochem. Anal.* 21, 328–339. doi: 10.1002/pca.1203

Simon, A., Chulia, A. J., Kaouadji, M., P. Allais, D., and Delage, C. (1993). Phytochemistry of the Ericaceae. Part 3. Further flavonoid glycosides from *Calluna vulgaris*. *Phytochemistry* 32, 1045–1049. doi: 10.1016/0031-9422(93)85253-n

Wang, Z., Zhu, W., Liu, H., Wu, G., Song, M., and Yang, B., et al. (2018). Simultaneous determination of aesculin, aesculetin, fraxetin, fraxin and polydatin in beagle dog plasma by UPLC-ESI-MS/MS and its application in a pharmacokinetic study after oral administration extracts of *Ledum palustre* L. *Molecules* 23, 2285. doi: 10.3390/molecules23092285

Wollenweber, E., and Kohorst, G. (1984). Novel epicuticular leaf flavonoids from *Kalmia* and *Gaultheria* (Ericaceae). *Z. Naturforsch. C* 39C, 710–713. doi: 10.1515/znc-1984-7-804

Ye, C., Jin, M., Li, R., Sun J., Wang R., and Wang J., et al. (2020). Phytochemical and chemotaxonomic study on the leaves of *Rhododendron dauricum* L. *Biochem. Syst. Ecol.* 90, 104038. doi: 10.1016/j.bse.2020.104038
